# Mental healthcare in primary and community-based settings: evidence beyond the WHO Mental Health Gap Action Programme (mhGAP) Intervention Guide

**DOI:** 10.1136/ebmental-2021-300401

**Published:** 2022-04-26

**Authors:** Roxanne C Keynejad, Jessica Spagnolo, Graham Thornicroft

**Affiliations:** 1 Health Service and Population Research, King's College London Institute of Psychiatry Psychology and Neuroscience, London, UK; 2 Département des sciences de la santé communautaire, Université de Sherbrooke, Sherbrooke, Canada; 3 Centre de recherche Charles-Le Moyne, Campus de Longueuil, Université de Sherbrooke, Sherbrooke, Canada

**Keywords:** adult psychiatry, child & adolescent psychiatry, depression & mood disorders, anxiety disorders, schizophrenia & psychotic disorders

## Abstract

**Objectives:**

The WHO’s Mental Health Gap Action Programme Intervention Guide (mhGAP-IG) has been widely used in low and middle-income countries. We reviewed literature describing interventions and training programmes beyond the mhGAP-IG, in primary healthcare (PHC) and community-based healthcare (CBH).

**Design:**

We searched studies excluded from our updated mhGAP-IG systematic review, and included in other relevant systematic reviews, for evidence and experience of initiatives integrating mental health into PHC and CBH. Our 24 November 2020 mhGAP-IG search encompassed MEDLINE, Embase, PsycINFO, Web of Knowledge, Scopus, CINAHL, LILACS, ScieELO, Cochrane, PubMed databases, 3ie and Google Scholar. Although heterogeneity prevented meta-analysis, we descriptively summarised the evidence-base.

**Results:**

Out of 1827 results, we identified 208 relevant records. They described randomised controlled trials of mental health interventions (98 studies, n=55 523 participants), non-randomised studies measuring clinical outcomes (22 studies, n=7405), training outcomes (36 studies, n=12 280) and implementation outcomes (21 studies, n=1090), plus descriptive accounts (18 studies, n=2526), baseline surveys and exploratory studies (6 studies, n=17 093) and commentaries (7 studies). Most (40%) were conducted in the African region, region of the Americas (16%), and South-East Asia (13%). Randomised and non-randomised studies reported improved symptoms, substance use, functioning, parenting and child outcomes. Non-randomised studies reported improved clinical knowledge, confidence and skills following training.

**Conclusions:**

The literature beyond the mhGAP-IG is extensive and shares common findings. Future priorities are less-studied regions, interventions for severe mental illness, exploring ways that mhGAP-IG and alternative approaches complement each other in different contexts and scaling-up mental health integration.

PROSPERO registration number

CRD42017068459.

**Key messages**

**What is already known on this topic**

- The WHO’s Mental Health Gap Action Programme Intervention Guide (mhGAP-IG) provides clinical guidelines to enable non-specialist staff to deliver evidence-based mental healthcare in primary healthcare (PHC) and community-based healthcare (CBH) settings.
- Despite widespread implementation, alternative approaches to integrating mental health into PHC and CBH continue to be developed and evaluated.

**What this study adds**

- This review identified and synthesised the evidence base for approaches to integrating mental health into PHC and CBH beyond the mhGAP-IG, worldwide.
- We identified a substantial literature, not only from the African and South-East Asian regions but also from locations in which the mhGAP-IG has been less widely implemented, including the region of the Americas.

**How this study might affect research, practice and/or policy**

- Common findings with the mhGAP-IG evidence base support the need for research and policy addressing barriers to successful integration of mental health into PHC and CBH settings, and the need to prioritise neglected disorders, including severe mental illness.

## Background

The WHO^1^ recommends addressing the gap between the need for and provision of mental, neurological and substance use disorder (MNS) services^2 3^ through detection, diagnosis and management in primary healthcare (PHC) and community-based healthcare (CBH) settings.^2^ WHO^4^ and expert consensus^5^ recommend stepped care, where mental health treatment and its intensity are personalised to meet individuals’ needs. Most stepped care should be delivered through PHC and CBH, because of proximity to people’s homes, delivery by staff who know the community and ease of follow-up. More complex or severe mental health needs requiring specialist care can be supported by secondary healthcare.

During public health emergencies such as the coronavirus pandemic, clinical priorities and care pathways are adjusted, and travel to centralised services may be compromised, increasing the need for stepped care.^6^ WHO and the Organisation for Economic Cooperation and Development identified the importance of strong PHC to the COVID-19 response, for maintaining continuity of care, and managing mental health impacts of the pandemic.^7 8^

The availability of PHC and CBH personnel, trained in mental health, is essential to stepped mental healthcare. However, there is a chronic shortage of non-specialists trained in mental healthcare in low and middle-income countries (LMICs).^3^ WHO^9^ recommends task sharing: the redistribution of tasks to staff with less specialised training, where appropriate. Public health emergencies exacerbate the need for task-shared mental healthcare, given surges in healthcare demand.^7 8^ Integrating psychological interventions into universal health coverage and emergency preparedness plans are key priorities for ‘building back better’ post-COVID-19.^10^

The WHO Mental Health Gap Action Programme (mhGAP) was launched in 2008, to address the gap between the need for and availability of mental healthcare.^11^ The mhGAP Intervention Guide (IG),^12 13^ humanitarian IG (HIG)^14^ and app (e-mhGAP)^15^ provide clinical guidelines to help non-specialists offer evidence-based mental healthcare in PHC and CBH. Two systematic reviews^16 17^ identified widespread mhGAP-IG implementation. They identified 195 peer-reviewed studies and protocols, which used the mhGAP-IG, HIG or app, reflecting adoption by clinicians, governments, non-governmental organisations and researchers in diverse settings.

In the most recent review,^17^ the most common applications of mhGAP-IG were training courses (58 studies), clinical guidelines (n=46) and research (n=25), such as the ‘enhanced usual care’ arm in randomised controlled trials (RCTs). The remaining applications comprised local, contextual adaptations (n=12), economic analyses (n=7) and other educational applications (n=7). The most common settings were the (WHO-designated) African region (n=62) and South-East Asian region (n=38). In addition to peer-reviewed literature, a review of ‘grey literature’ identified 151 documents describing mhGAP-IG implementation and use.^18^ Most grey literature came from the region of the Americas and African region.^18^

While the reach of the mhGAP-IG, HIG and app in LMICs is impressive, extensive literature on integrating mental health into PHC and CBH predates their inception or employs alternative approaches.^11^ Limited access to support from and collaboration with Ministries of Health, WHO and other institutions^18^ could contribute to lower mhGAP-IG implementation in some regions. Other reasons for variable adoption could include criticisms that it sets ‘the epistemological parameters of its own critique’ through algorithmic understandings of mental ill-health,^19^ which may be culture and/or context-dependent, rather than a ‘universal tool’.^20^ The mhGAP-IG is positioned as a guide requiring local adaptation and includes an essential care and practice module, focused on transdiagnostic principles of quality care. However, mhGAP-IG has been accused of limiting the ability of ‘local contextual epistemologies of distress… to interrupt or resist’ its conceptualisation of mental ill-health.^20^ For these and logistical reasons (such as lack of locally translated manuals and training packages), alternative models of PHC and CBH provision continue to be developed, implemented, and evaluated. Focusing restrictedly on mhGAP-IG implementation risks neglecting insights and experiential learning from alternative approaches to integrating mental health into PHC and CBH.

A recently-updated^21^ Cochrane systematic review^22^ meta-analysed quantitative evidence of non-specialist health worker interventions for MNS disorders in LMICs. The updated review^21^ identified 95 RCTs of PHC interventions for people with mental disorders and distress, or their carers, in LMICs (compared with 38 studies of any design in 2013). Meta-analyses led the authors to conclude that PHC interventions showed promise for common mental disorders (CMDs), postnatal depression, post-traumatic stress, harmful substance use and distress among dementia carers. A systematic review of 24 qualitative studies of PHC mental health programmes in LMICs^23^ found that PHC investment, health worker capacity building and addressing service users’ social needs were key priorities.

Other reviews meta-analysed LMIC RCTs of psychological treatments for depression and anxiety disorders (n=17 interventions),^24^ CMDs (n=27),^25^ perinatal depression (n=9),^26^ perinatal CMDs (n=10)^27^ and adult (n=36)^28^ and child (n=11) mental health in humanitarian settings.^29^ Systematic reviews have also synthesised evidence on psychological interventions for CMDs among people with HIV in LMICs (n=5),^30^ staff mental health training in Africa (n=37)^31^ and WHO mental health training (n=29) worldwide.^32^ Thus, to our knowledge, no reviews have synthesised worldwide evidence (including high-income countries: HICs) for broader mental health integration within PHC and CBH, beyond the mhGAP-IG.

## Objective

We aimed to identify and synthesise evidence for programmes outside the WHO mhGAP-IG, HIG and app, which integrated mental health into PHC and CBH.

### Study selection and analysis

Given prior systematic reviews with a narrower focus, we adopted a pragmatic approach, following principles advocated by WHO.^33^ We reviewed peer-reviewed, published evidence, for mental health integration into PHC and CBH, by first screening all studies excluded from our previous systematic review of mhGAP-IG applications.^17^ This work is registered on the PROSPERO international prospective register.

We originally searched the following databases on 24 November 2020: 3ie, Cochrane Library, CINAHL, EMBASE, LILACS, Medline, PsycInfo, PubMed, SciELO, Scopus, Web of Knowledge and Google Scholar. Search terms were ‘mental health gap action programme’ OR ‘mental health gap action programme’ OR ‘mhGAP’. We searched these databases in English, but results in other languages were eligible. We screened individual articles and the reference lists of relevant systematic reviews, for eligibility.

RCK removed duplicates and then screened titles and abstracts for eligibility. Inclusion criteria were: any study design describing evidence or experience of integrating mental healthcare into PHC and CBH without using the mhGAP-IG, conducted in any country (including HICs), reported in any language. JS screened the list of relevant systematic reviews, before extracting first author, publication year, country of implementation, study design, sample and findings from each eligible study, into a table. RK reviewed the eligibility of studies for which inclusion was uncertain. On 21 February 2022, we searched for published records of included protocol results.

We used the WHO definition of PHC,^34^ which we interpreted to mean healthcare delivered via community encounters (such as clinics and home visits, rather than hospital settings). We categorised included records into types, to organise the results. Where more than one type was relevant (eg, studies evaluating both clinical and training outcomes), we made a judgement about the primary focus. The heterogeneity of models of mental health integration into PHC and CBH precluded meta-analysis. Given the heterogeneity of most included studies employing non-randomised designs, and variable methods used, we did not assess risk of bias.^35^

## Findings

**Figure 1:**
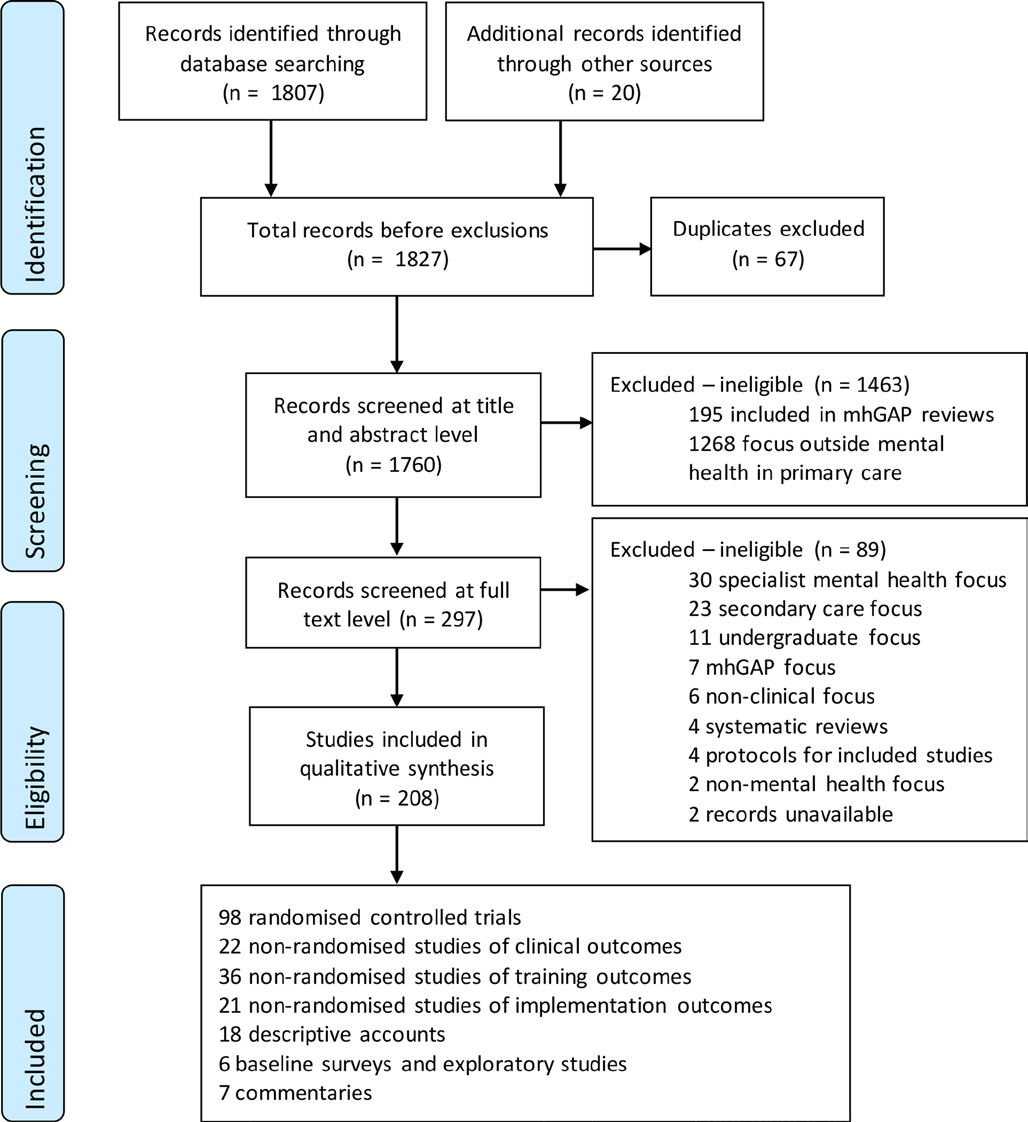
PRISMA flow diagram of study identification, screening, eligibility and inclusion. mhGAP, Mental Health Gap Action Programme; PRISMA, Preferred Reporting Items for Systematic Review and Meta-Analysis.

Figure 1 shows the flow of studies from identification to screening, eligibility and inclusion. Of 1827 records screened, 1619 were excluded, as they did not report evidence or experience of integrating mental health into PHC, outside of the mhGAP-IG and associated tools. Of 297 studies reviewed at full text, 208 were included in this review. Online supplemental tables 1–7 summarise all included studies and extracted data. No records were excluded based on language.

10.1136/ebmental-2021-300401.supp1 Supplementary data

Table 1 shows the distribution of study types across regions. Most (40%, n=83) were conducted in the WHO African region, followed by region of the Americas (16%, n=34) and South-East Asian region (13%, n=27). Eastern Mediterranean and Western Pacific regions contributed a further 9% of studies (n=19 each). The European region contributed 5% (n=12), 3% were conducted across multiple regions (n=7), and 3% (n=7) were non-regional commentaries.

**Table 1:** Studies reporting integration of mental health into PHC and CBH, by type and geographical region

Types →	RCTs	Non-randomised: clinical	Non-randomised: training	Non-randomised: implementation	Descriptive accounts	Baseline surveys, exploratory studies	Commentaries	Total
Regions ↓								
Total participants	55 523	7405	12 280	1090	2526	17 093	N/A	95 647
African region	34	10	15	15	5	4	0	83
Region of the Americas	16	5	5	3	5	0	0	34
South-East Asia	16	2	5	2	1	1	0	27
Eastern Mediterranean	15	2	1	0	1	0	0	19
Western Pacific	9	2	6	1	1	0	0	19
European region	6	1	4	0	1	0	0	12
Multiregional	2	0	0	0	4	1	0	7
Non-regional	0	0	0	0	0	0	7	7
Total	98	22	36	21	18	6	7	208

CBH, community-based healthcare; PHC, primary healthcare; RCTs, randomised controlled trials.

Included studies described RCTs assessing mental health intervention impacts in PHC and CBH (47%, n=97, online supplemental table 1), non-randomised studies measuring clinical outcomes (11%, n=22, online supplemental table 2), training outcomes (17%, n=36, online supplemental table 3) and implementation outcomes (10%, n=21, online supplemental table 4) as well as descriptive accounts (9%, n=18, online supplemental table 5), baseline surveys and exploratory studies (3%, n=6, online supplemental table 6) and commentaries (3%, n=7, online supplemental table 7).

### RCTs of mental health interventions in PHC and CBH

Of 208 included studies, 98 (47%) were RCTs assessing mental health intervention impact in PHC and CBH (online supplemental table 1). Eighteen (18%) were conducted in HICs. Interventions evaluated using RCTs largely targeted disorders featured in the mhGAP-IG. These included CMDs depression, anxiety, post-traumatic stress disorder (PTSD) symptoms, trauma, distress, schizophrenia, dementia, epilepsy and substance use, including during pregnancy and adolescence. Psychological interventions were commonly evaluated, including motivational enhanced therapy, cognitive-behavioural therapy (CBT), behavioural activation, self-help plus, problem management plus, cognitive processing therapy, common elements treatment approach, interpersonal therapy, rational emotive therapy, creative play therapy, narrative exposure therapy, problem-solving therapy, thought field therapy, and testimony therapy. Most clinical interventions were recommended in the mhGAP-IG, often centring on psychoeducation, education support sessions and strength-based case management. Psychosocial support was frequently mentioned, including through motivational interviewing, trauma counselling and after screening for substance use.

Other interventions included home visits, exercise programmes, pharmacotherapy and collaborations between PHC staff to holistically address health and social determinants. A proportion of RCTs also described the training provided to a range of counsellors, nurses, peer and group leaders, community health workers, volunteers, non-specialist refugee or migrant facilitators, therapists, lay facilitators, social workers, PHC staff and research assistants.

RCTs reported depression remission, reduced depressive symptoms and prevalence, CMD symptoms, psychiatric morbidity and severity of mental health conditions. Findings also showed reduced substance use and symptom scores, levels of stress, emotion regulation intensity in children, caregiver burden, caregiver mental health and distress, trauma, anxiety, stress, PTSD symptoms, anger, aggression and intimate partner violence.

Included RCTs reported improved functioning, well-being, quality of life, recovery from CMDs, treatment completion and reach, medication adherence, substance use abstinence, health-related self-efficacy, self-care, nutrition, overall health behaviours, contraception use, breast feeding and service satisfaction.

Studies identified impacts on knowledge about parenting, knowledge and attitudes towards infant development, parenting self-efficacy and competence, home safety attitudes, social support and interpersonal relationships, children’s weight gain or healthy weight, self-rated harsh discipline, positive behavioural management, caregiver–child interactions, initiation of communication by children, mood and mothers’ health and mental health. RCTs also reported reduced separation distress in infants, child internalising behaviours, improved cognitive and receptive language scores, motor skills, adaptive behaviours, emotion regulation and school behaviour.

Studies which also evaluated the training delivered to non-specialist providers reported its acceptability and feasibility, increased confidence in diagnosing or treating depression, improved routine detection of mental health conditions, higher digital training completion, greater objective competence and subjective confidence in applying CBT techniques.

### Non-randomised studies measuring clinical outcomes

Of 208 included studies, 22 (11%) were non-randomised studies measuring clinical outcomes of initiatives integrating mental health into PHC and CBH (online supplemental table 2). Four (18%) were conducted in HICs. Ten studies were non-randomised trials, comparing an intervention to a control group; a further 12 were uncontrolled. Non-randomised clinical interventions targeted CMDs, depression, anxiety, PTSD symptoms, substance use, stress, distress, hopelessness, social support, self-esteem, disability, quality of life, functioning, adolescent emotional and behavioural problems and parental action. Some targeted multiple symptoms and disorders.

Non-randomised clinical interventions included CBT, problem-solving therapy, behavioural activation, interpersonal psychotherapy, trauma narrative and grief-processing, trauma-focused psychosocial cultural treatment, psychoeducation, general mental health training, psychological first aid, peer support, social activities, sociotherapy, social skills training, attachment-focused therapy, parenting skills training for children with behavioural problems, education workshops, structured activities for children, psychiatrist treatment and medication. Some studies trained lay persons, lay health workers, community health workers, medical assistants, nurses, clinical psychologists, counsellors, physicians and volunteers to deliver interventions, over 4–10 days. A small proportion mentioned supervision. Some non-randomised clinical studies used active comparison arms, such as half-day depression training for all PHC and mental health-employed physicians, national clinical guideline implementation, or weekly group meetings to discuss problem-solving therapy materials and telephone support from trained coaches.

Non-randomised clinical interventions targeted adults with mental health conditions, depression, PTSD, schizophrenia and psychological distress. Participants included women, mothers, children and adolescents, migrant farm workers, parents, guardians and caregivers and healthcare staff.

Non-randomised clinical intervention studies reported reduced CMD symptoms, depression, anxiety, PTSD, trauma and shame, alcohol use, stress, distress, hopelessness, anger and disability, post-intervention. They also reported improved treatment adherence, awareness of alcohol risks and consequences, self-esteem, functioning, quality of life and behaviour. Studies reported increased social support, parental support, positive coping skills, HIV knowledge, providers’ understanding of patients’ concerns, attitudes and acceptance of mental ill-health and reduced medication costs, hospital days, stigma and absenteeism.

### Non-randomised studies measuring training outcomes

Of 208 included studies, 36 (17%) were non-randomised studies of mental health integration into PHC and CBH focused on training outcomes (online supplemental table 3). Ten (28%) were conducted in HICs. Most non-randomised training studies were uncontrolled, reporting changes post-training in comparison to pre-training. Four studies compared one or more training interventions to no intervention, or alternative training. Most studies reported quantitative outcomes; a minority conducted mixed methods or qualitative evaluations.

Training content addressed general mental health topics, depression, alcohol use, child and adolescent mental health, HIV-associated neuropsychiatric complications, CBT, motivational interviewing, solution-focused brief therapy, microcounselling skills, mental health service pathways, specific assessment tools, and Mental Health First Aid.

Trainees included nurses, general practitioners, PHC, CBH or mental health staff, patients, non-health professionals, PHC staff tutors and traditional practitioners. Several studies trained multidisciplinary groups together. Findings included improved clinical competence, patient-centred care, self-reported changes in practice and confidence, knowledge and reduced stigma. Training outcomes included higher retention in a small group than self-directed format.

### Non-randomised studies measuring implementation outcomes

Of 208 included studies, 21 (10%) were non-randomised studies of mental health integration into PHC and CBH, focused on implementation outcomes (online supplemental table 4). One (5%) was conducted in a HIC. Most study designs used qualitative interviews or focus groups with intervention recipients, training recipients or stakeholders. Some studies evaluated the feasibility of mental health interventions in PHC and CBH, including one process evaluation. Interventions included therapies, mental health training and service delivery models.

Non-randomised implementation studies reported that PHC and CBH interventions and training programmes were acceptable, perceived as beneficial and associated with clinical or skill improvements. Challenges and barriers included variability of prior mental health training, the need for continued supervision and training, lack of motivation to attend or continue attending sessions, differing cultural conceptions of mental ill-health, stigma, competing demands on staff and patients’ time, low managerial prioritisation and financial and resource constraints. Facilitators included experiential learning using videoconferencing, social networks’ encouragement to attend sessions, coordination of training by community members and long-term training.

### Descriptive accounts

Of 208 included studies, 18 (9%) were descriptive accounts of mental health integration into PHC and CBH (online supplemental table 5). They described community mental health service delivery and training, situational analyses, policy document reviews and health systems strengthening. Three (17%) focused on HICs.

Findings included reduced emergency psychiatric presentations following community mental healthcare, the need for mental health legislation and policy reform, scaling up PHC mental health training and balanced care models integrating PHC and secondary mental healthcare and the potential for humanitarian responses to develop sustainable mental healthcare.

Opportunities included service user, caregiver, traditional healer and religious leader engagement. Challenges included continuity of care, providing services in rural areas, clinical complexity, limited mental health specialists, staff and supervisor attrition, limited provision for psychiatric emergencies, competing priorities, sporadic medication supply, constrained budgets and stigma.

### Baseline surveys and exploratory studies

Of 208 included studies, six (3%) were baseline surveys and exploratory studies of mental health integration into PHC and community settings (online supplemental table 6); all were conducted in LMICs. Participants included doctors, nurses, midwives and other community or PHC staff. Non-survey studies comprised a census of community psychiatric consultations and a qualitative document review of resources for integrating mental health into PHC.

### Commentaries

Of 208 included studies, seven (3%) were commentaries on mental health integration into PHC and CBH (online supplemental table 7). Commentaries advocated scaling-up mental health services, more task-shared and community services, caregiver skills training, training adaptation for the context, improved intervention study reporting, greater focus on comorbid physical illnesses, integrating digital clinical records, rights-based approaches to mental healthcare, investment in research and innovation, mental health policymaking, community awareness-raising and increased mental health budgets.

## Conclusions and clinical implications

This review of non-mhGAP initiatives integrating mental health into PHC and CBH worldwide identified a range of literature describing RCTs of primary and community mental health interventions, non-randomised studies measuring clinical outcomes, training outcomes and implementation outcomes, descriptive accounts, baseline surveys and exploratory studies and commentaries, of which between 72% and 100% were conducted in LMICs. Targeted disorders largely corresponded to those addressed in the mhGAP-IG, while clinical interventions were almost exclusively those recommended by the mhGAP-IG, bar a small number of trauma-focused studies.^36–38^ Randomised and non-randomised studies reported improvements in clinical outcomes, including symptoms, substance use, functioning, parenting and child outcomes. Non-randomised studies reported improved clinical knowledge, competence, confidence and skills following mental health training, alongside barriers to implementation. Descriptive accounts, baseline surveys and commentaries identified challenges, opportunities and future priorities.

In comparison to our previous systematic reviews of mhGAP-IG evidence,^17 39^ we identified a higher proportion of RCTs evaluating clinical interventions, perhaps reflecting the unlimited timeframe, relative to the 11 years since the mhGAP-IG was first launched. Smaller proportions evaluated mental health training and contextual adaptations than in our mhGAP-IG reviews, but a greater proportion employed implementation science methods. Although this review included studies from HICs, 40% were from the African region (similar to our mhGAP-IG review). A higher proportion was from the region of the Americas (16% compared with 4% of mhGAP-IG studies) and a lower proportion from South-East Asia (13% compared with 25% of mhGAP-IG studies). Evidence from the Americas is particularly valuable, since our mhGAP-IG systematic reviews identified no evidence from this region.

Clinical interventions focused on psychoeducation, psychosocial support and brief psychological therapies (including recent innovations, such as self-help plus^40 41^ and problem-management plus),^42–46^ all of which are recommended by the mhGAP-IG and associated resources. Similarly, interventions targeted depression, substance use and child and adolescent mental health conditions: all mhGAP-IG modules. Notably, psychotic disorders and dementia were less frequently addressed than CMDs. Despite their inclusion in the mhGAP-IG, our other reviews also found a paucity of research on severe mental illness (SMI) and dementia.^17 39^ There is, therefore, a need for research on integrating SMI and dementia into PHC and CBH.

Many included interventions did, however, target anxiety symptoms, which lack a dedicated mhGAP-IG module, and PTSD symptoms, which are addressed in a separate mhGAP-IG module^47^ and the HIG.^14^ We identified evidence from several regions, where mhGAP-IG and alternative approaches have been implemented, suggesting future research should explore how these approaches complement each other.

The depth with which interventions were described and evaluated varied considerably, perhaps indicating the benefits of standardised programmes, which can be adapted to the context. However, stigma, cultural differences and differing perspective on treatments can be barriers to mhGAP-IG implementation and use.^17 39^ Following the recommendation to locally adapt the mhGAP-IG is not always described; a recent framework has been published, which guides mhGAP-IG cultural and contextual adaptation.^48^ While we previously identified 15 studies reporting local mhGAP-IG adaptation or contextualisation,^17 39^ only three non-mhGAP-IG studies identified by this review discussed intervention adaptation. This may suggest that non-mhGAP-IG interventions may have been developed or chosen specifically to meet the realities of PHC and CBH settings in which they were implemented, being judged more appropriate.

The literature on mental health integration into PHC and CBH beyond the mhGAP-IG demonstrates substantial commonality. Barriers to implementation, challenges and opportunities in the non-mhGAP-IG literature had much in common with the mhGAP-IG literature. Insufficient basic mental health training meant that PHC staff members’ interest in and understanding of mental health was often low. Competing priorities and lack of prioritisation by managers limited staff motivation for training, acquiring and implementing new mental health skills. The need for ongoing supervision and refresher training within a task-sharing model was often under-resourced. Resource constraints were a common barrier. Stakeholder support for mental healthcare, novel technologies and longer term approaches to mental health integration was facilitators also found by mhGAP-IG reviews.

Our search strategy enabled us to compare learning from mhGAP-IG literature with other relevant studies, using efficient and transparent methods to avoid duplicating existing systematic reviews. A limitation is that we did not conduct a dedicated systematic review of all published studies in this field, raising the risk of missing some relevant evidence. Our study complements the existing literature, however, by synthesising a variety of study designs, capturing the diversity of global evidence for integrating mental health into PHC and CBH, beyond the WHO mhGAP-IG.

Future priorities are less-studied regions, interventions for SMI, understanding how mhGAP-IG and alternative approaches can complement each other and scaling-up mental health integration beyond brief research studies and grant-funded projects.^49 50^

## Data Availability

Data sharing not applicable as no datasets generated and/or analysed for this study.
