# Supplementary material for: Mental healthcare in primary and community-based settings: evidence beyond the WHO Mental Health Gap Action Programme (mhGAP) Intervention Guide
Source: Evid Based Ment Health. 2022 Apr 26;25(e1):e1–7. doi: 10.1136/ebmental-2021-300401 (PMC9811100; doi:10.1136/ebmental-2021-300401)
Supplement: Supplementary data [file ebmental-2021-300401supp001.pdf]

Table of Contents

Supplementary Online Table 1: Randomised Controlled Trials 2
Supplementary Online Table 2: Non-randomised trials for clinical outcomes 25
Supplementary Online Table 3: Non-randomised trials for training outcomes 30
Supplementary Online Table 4: Non-randomised studies for implementation outcomes 37
Supplementary Online Table 5: Descriptive accounts 42
Supplementary Online Table 6: Baseline surveys and exploratory studies 48
Supplementary Online Table 7: Commentaries 50
References 51

Supplementary Online Table 1: Randomised Controlled Trials

**Authors**	**Country**	**Study design**	**Sample**	**Summary of findings**	**mhGAP-IG recommended intervention or approach?**
Araya et al., 2003 (1)	Chile	RCT assessing the effectiveness of a stepped-care programme on depressive symptoms among low-income women.<br>Intervention: 7 weekly sessions of structured group psychoeducation (+2 optional booster sessions), information manuals, clinical progress monitoring and structured pharmacotherapy for severe/persistent depression. Psychoeducation groups included 20 participants. Topics included information about depression symptoms, causes, treatment, scheduling positive activities, problem-solving, cognitive and relapse-prevention techniques. Group leaders monitored medication adherence and attended follow-up sessions.<br>Training: Group leaders received 12 hours' training and 8 hours' supervision. Physicians received 4 hours' training on pharmacotherapy.<br>Control: usual PHC + guidelines on depression in PHC.	240 female PHC patients with major depression: 120 randomised to intervention group; 120 randomised to the control group.	Between-group differences in all outcomes (depression, functional impairment) in favour of the intervention, maintained at 6 months' follow-up.	Yes
Langer et al., 1996 (2)	Argentina, Brazil, Cuba, Mexico	RCT assessing a psychosocial support intervention, which aimed to improve perinatal health and women's psychosocial conditions.<br>Intervention: 4-6 home visits by female social workers/obstetric nurses, counselling and emotional support, information about pregnancy and delivery, reinforcement of health service utilisation, social network strengthening. Home visits were offered around 22nd, 26th, 30th and 34th weeks of gestation.<br>Control: routine antenatal care as usual.	2236 women at high risk of low birthweight: 1110 randomised to intervention group; 1125 randomised to control group.	No difference between groups in perceived social support, satisfaction with reproductive experience, maternal or newborn healthcare, post-participation.	Yes
Robledo-Colonia et al., 2012 (3)	Colombia	RCT assessing a supervised exercise programme for antenatal depression.<br>Intervention: 3x physiotherapist and physician-supervised 60-minute exercise classes per week in groups of 3-5 women. Sessions began between weeks 16 and 20 of gestation and continued for 3 months, Exercise included walking, aerobic exercise, stretching and relaxation. The physiotherapist encouraged adherence.<br>Control: no exercise classes.<br>Both groups received usual prenatal care (1 session per week for 3 months) and continued their usual physical activity.	80 pregnant women attending ANC.<br>40 participants assigned to intervention group; 40 participants assigned to control group.	Women in the intervention group showed reduced CES-D depressive symptoms in comparison to control group women, post-participation.	Yes

Baker-Henning-ham et al., 2005 (4)	Jamaica	Cluster RCT assessing the impact of early childhood stimulation on maternal depression and child development.<br>Intervention: women with undernourished children received weekly home visits by community health aides for 1 year. Visits lasted ~ 30 minutes. Women were taught play activities with homemade materials. Parenting issues were discussed and other family members present were encouraged to participate. Intervention participants also received standard health and nutrition care.<br>Training: community health aides received 4 weeks' training on health and nutrition and 2 weeks' training on child development, parenting and intervention conduct.<br>Control: standard health and nutrition care only.	18 government health centres: 11 randomised to intervention group (n=76 participants); 7 randomised to control group (n=70).	Intervention group women reported a significant reduction in the frequency of CES-D depressive symptoms, post-participation, relative to the control group. Women receiving 25-39 or >40 visits benefited significantly. Women receiving <25 visits did not benefit from the intervention.<br>Post-intervention, maternal depression was significantly negatively correlated with male children's developmental quotient, only (Griffiths Mental Development Scales).	Yes
Babor and Grant, 1992 (5)	Costa Rica, Mexico, USA Norway, UK, Bulgaria, Russia, Kenya, Zimbabwe, Australia	RCT assessing a brief intervention for hazardous alcohol use.<br>Interventions: 1) simple advice group using General Health Interview + 5 minutes' advice on 'sensible' drinking. 2) Brief counselling group: same 5-minutes' advice + additional 15 minutes' counselling. Participants received self-help manual: encouraged to use to develop plan to break drinking habit.<br>Control: 20 minute health interview.	1655 non-alcoholic heavy drinkers (1356 males, 299 females).	Both interventions associated with significant reductions in mean alcohol consumption and intensity of drinking in males. Both interventions also associated with significant reductions in alcohol use among females.	Yes
Ernst et al., 1999 (6)	USA	Partially randomised controlled trial assessing the Seattle model of paraprofessional advocacy, for substance use, changes in life circumstances, family planning, maternal and child health and use of community services over 36 months.<br>Intervention aims: To provide children with comprehensive services. To assist mothers to obtain treatment, maintain recovery and resolve challenges associated with substance use, holistically, while recognising their complex difficulties.<br>Intervention: Seattle model.<br>Control: contact by telephone or letter every 6 months for 3 years but no advocacy.	Community referred or hospitalised high risk women using substances, allocated to intervention (n=65) or control (n=31) group.	Engagement with advocacy was associated with inpatient treatment completion, abstinence from drugs or alcohol, regular contraception use relative to disengaged intervention group women, and satisfactory receipt of services relative to control group women.	Yes

Rosenberg et al., 2002 (7)	USA	RCT assessing supportive paraprofessional home visiting for women with children at risk of developmental disabilities, deemed to be receiving inadequate parenting. <u>Intervention:</u> services from 3 paraprofessionals + 1 nurse supervisor. Paraprofessionals were women parents of children with special care needs, visiting the homes of ~30 families, for 12 months each. Worked with families to develop plans to achieve their goals. <u>Training:</u> 3 months on family-centred, culturally sensitive services, service coordination, child development and safety. <u>Control:</u> 1-2 home visits by registered nurse with experience working with families and children with special needs, serving 70 families. Thereafter, nurse participation in care conferences, clinic appointments or monthly phone calls.	88 families randomised to intervention group, 71 randomised to control group.	Intervention group women rated services more helpful and had moderately better mental health (problem severity profile scale) than control. Children of intervention group women more likely to receive Individualised Family Service Plan than control group women's children. Groups did not differ on quality of home environments, service utilisation or child outcomes like child maltreatment or out-of-home placements.	Yes
Roman et al., 2007 (8)	USA	RCT comparing a nurse-CHW team visiting intervention with standard community care, for Medicaid-eligible pregnant women. <u>Intervention:</u> teams of 1 nurse + 2 CHWs, following visit guidelines for expected care for 50-60 families. Initial team visit followed by individual visits unless necessary for crises. Nurses led multidisciplinary assessment, provided crisis intervention and case management, assessed and managed health issues and met prenatal providers. CHWs visited families fortnightly during pregnancy (weekly if necessary after birth). At 6-months, visits reduced to monthly or maintained fortnightly. CHWs delivered manualised activity modules adapted from Building Strong Families programme modules. <u>Aim</u>: to reduce stress, reduce depression symptoms and increase protective psychosocial resources. <u>Control:</u> usual community care including enhanced Medicaid. Key differences included direct contact, less intensive contact, lack of basic needs attention.	Intervention group (n=226) Control group (n=264).	Significantly higher contact rate in intervention group than control group. More high-risk women reached by intervention group team than control group team, except for women with drug and alcohol problems.	Yes
Roman et al., 2009 (9)	USA	RCT assessing the impact of a nurse-CHW home visiting team within Medicaid enhanced perinatal services on pregnant women's depression, stress, psychosocial resources. <u>Intervention:</u> As above. <u>Control:</u> usual community care including enhanced Medicaid with fewer face-to-face contact visits.	Intervention group (n=307) Control group (n=306)	Intervention group women had significantly fewer depressive symptoms than control group women, post-intervention. Women with lower psychosocial resources, higher stress, or both improved most. Intervention group reported less stress and greater mastery than control group women. There were no differences between groups in self-esteem or social support.	Yes

Walkup et al., 2009 (10)	USA	RCT assessing the impact of a paraprofessional-delivered, home-visiting intervention among young Native American mothers on parenting knowledge, involvement, maternal and infant outcomes.<br>Intervention: Family Spirit: 25x 1 hour visits by 2 paraprofessionals following American Academy of Paediatrics recommendations (Caring for Your Baby and Child: Birth to Age 5.<br>Active control: 23x 1 hour breast-feeding/nutrition education visits.<br>Both interventions began during pregnancy and continued until 6 months postpartum. Interventionists were local community members who received 500 hours' training in home-visiting methods and curricular content.	Intervention group (n=81)<br>Active control group (n=86).	Intervention group women gained more parenting knowledge (multiple choice test) at 6 and 12 months postpartum than active control group women. Intervention group women reported significantly fewer externalising symptoms and separation distress (internalising at 12 months postpartum, (Infant Toddler Social Emotional Assessment). No differences between groups in maternal involvement (SAMHSA), home environment (HOME), women's stress (PSI), social support (Social Support Scale), depression (CES-D) or substance use (SAMHSA).	Yes
Waitzkin et al., 2011 (11)	USA	Cluster RCT + ethnographic techniques assessing the impact of briefly trained CHWs (*promotoras*) on depression care in community health centres.<br>Intervention: PHC practitioners + promotoras collaborated, following a clinical algorithm. PHC practitioners prescribed medication or arranged mental health consultations based on PHQ-9 score. Promotoras referred patients for employment, housing, food or violence-related support and tracked progress monthly.<br>Control: screening for depression with PHQ-9 and informing PHC practitioners.	120 people with depression participated in the study across 2 CHCs	Unemployment, housing insecurity, food insecurity and violence were strong predictors of depression. The intervention was not associated with statistically significant improvements in depression. Ethnographic methods identified positive responses to the intervention.	Yes
Ginsburg et al. 2012 (12)	USA	RCT assessing the feasibility of depression prevention programme for pregnant Native American adolescents and young adults. Both arms: 8x 30-60 minute sessions at home or in a clinic, initiated before 29 weeks' gestation with 3x booster sessions.<br>Intervention 1: Living in Harmony programme: CBT-based.<br>Intervention 2: educational support sessions on pregnancy and parenting.	Pregnant adolescents and young adults:<br>Intervention 1 (n=22)<br>Intervention 2 (n=25)	Women in both arms showed similar reductions in depression symptoms (CES-D), major depressive disorder (computerised diagnostic interview, EPDS), global functioning at all assessments (4, 12, 24 weeks post-intervention). No differences between arms in social support.	Yes

Barlow et al., 2013 (13)	USA	RCT assessing a Native American paraprofessional-delivered intervention. <u>Intervention:</u> Family Spirit + optimised standard care: 43x 1 hour one-to-one at-home structured lessons using table-top flipcharts, summary handouts. Included parenting skills, drug use prevention, life skills, positive psychosocial development. Biweekly until 4 months post-partum, monthly until 1 year, bimonthly until 3 years. <u>Control:</u> Transport to clinic visits, leaflets on services, referrals when needed.	Pregnant Native American adoles-cents from Arizona. Intervention group (n=159), control group (n=163)	Intervention group women had significantly greater parenting knowledge, self-efficacy, home safety attitudes, fewer externalising behaviours than control at 12 months post-partum. Children had fewer externalising problems than control. No difference in stimulation, support (HOME).	Yes
Barlow et al., 2015 (14)	USA	36 months post-partum follow-up of Family Spirit (Barlow et al., 2013; see above).	Pregnant Native American adoles-cents from Arizona. Intervention group (n=159), control group (n=163)	Intervention group women had greater parenting knowledge, locus of control (self-efficacy), less depression and externalising problems than control women at 36 months. They also had lower past month drug use. Intervention group children had fewer externalising, internalising, dysregulation problems than control children.	Yes
Williamson et al., 2014 (15)	USA	RCT assessing Madres a Madres parent training programme for immigrant Latina mothers and their children. <u>Intervention</u>: 4x 2 hour individual at-home sessions by promotoras (non-specialist coaches). Included normative child development, social competencies, positive parent-child interaction techniques, positive behavioural management strategies, service navigation. <u>Control</u>: waiting list.	Intervention group (n=113 mother-child dyads), Control group (n=81 mother-child dyads).	Intervention group women self-reported improved parenting skills, family support, family organisation, reduced child internalising behaviours (SCBE sub-scales) in comparison to control group women.	Yes
Magaña et al., 2015 (16)	USA	RCT assessing a culturally sensitive health education intervention for Latina women caring for youth and adults with intellectual and developmental disabilities. <u>Intervention:</u> 8x 1-2 hour at-home weekly sessions of manualised health education intervention (‘By Caring for Myself, I Can Care Better for My Family’) including taking care of oneself, healthcare, well-being activities, exercise, reducing stress and anxiety, personal growth. Delivered by trained peer leader. <u>Control:</u> participant manuals with intervention content, without home visits.	Intervention group (n=50), Control group (n=50).	Intervention group women had greater increases in health-related self-efficacy (CDSES), self-care, nutrition, overall health behaviours (FHS) than control group women. Both groups showed reduced depression symptoms (CES-D) and caregiver burden (caregiver appraisal measure).	Yes

Moore et al., 2016 (17)	USA	Pilot RCT assessing motivational enhanced therapy and strengths-based case management to reduce heavy drinking among Latino male day labourers.<br>Intervention: 3x weekly or fortnightly 45-55 minute sessions. Used goal setting and referral to medical, mental and social services. Manual was culturally adapted with input from volunteer peer leaders (promotoras), trained by psychologists.<br>Control: 1x customised brief feedback by trained research assistant immediately after baseline assessment with information about accessing services, if needed.	29 participants, most being from Mexico, and working as a day labourer	There were no statistically significant differences in alcohol use 6, 8 or 12 weeks after baseline. Comparative effect size of the intervention on weekly drinking was larger at 6 than 12 weeks. Statistically significant reduction in number of drinks over time for intervention group but not control participants.	Yes
Ekers et al., 2013 (18)	UK	RCT assessing 12 session behavioural activation (BA) therapy training on primary care mental health workers' knowledge and clinical skills.<br>Intervention: 5 day structured training course, including principles and evidence underpinning BA. Training used presentations, small and large groupwork, role play.<br>Control: usual care for adults with depression.	10 mental health professionals.	Training was highly acceptable (TARS).<br>No change in effect size of BA when adding this trial's results to a meta-analysis of studies using specialist therapists.	Yes
Purgato et al., 2021 (19)<br>Acarturk et al., 2022 (20)	Austria, Finland, Germany, Italy, UK Turkey	Two multicentre RCTs.<br>Intervention: Self-help Plus (SH+) group self-help based on acceptance and commitment therapy, delivered by briefly trained, non-specialist refugee/migrant facilitators, with a pre-recorded course and illustrated self-help book.<br>Control: routine social support and/or care following local regulations, information about health and social services and access to community networks supporting refugees and asylum seekers.	Purgato et al.: 459 refugee and asylum seekers with distress in Austria, Finland, Germany, Italy, UK.<br>Acarturk et al.: 642 Syrian refugees with psychological distress (GHQ) in Turkey.	Purgato et al.: No difference in mental disorder frequency on MINI at 6 months but significant reduction in mental disorders two weeks post-SH+.<br><br>Acarturk et al.: Significantly lower proportion receiving SH+ had MINI mental disorder at 6 months (21.7%) than EUC (40.7%) but not immediately post-SH+.	Yes
Knefel et al., (2020) (21)	Austria	Protocol: individual RCT.<br>Intervention: Problem Management Plus (PM+): transdiagnostic, low-intensity manualised psychological treatment for CMDs in communities exposed to adversity. Core strategies of 'managing stress', 'managing problems', 'get going, keep doing', 'strengthening social support'. Additional session on anger regulation/self-efficacy.<br>Control: waiting list for specialist mental health treatment + treatment as usual, e.g. contact with medical and mental health professionals, pharmacotherapy	120 Dari-speaking adult Afghan refugees or asylum seekers (60 per arm)	At baseline, 7 weeks and 6 months' follow-up:<br>Primary outcome: mental health (GHQ-28). Secondary outcomes: recent adverse life experiences (PMLDC), distress (ITQ), quality of life (WHOQoL-BREF), patient-centred therapy outcomes (PSYCHLOPS), integration (IPL-12), service use (CSRI).	Yes

Dybdahl, 2001 (22)	Bosnia and Herzegovina	RCT assessing psychosocial intervention for mental health, psychosocial functioning, intellectual abilities and physical health in Bosnia and Herzegovina post-war. Intervention: medical care + weekly 1 hour at-home intervention for 5 months, promoting development and well-being of young children through parental involvement and support. Included therapeutic discussion groups for trauma. Preschool teachers trained to facilitate group discussions to support mothers, increase their well-being. Group leaders met weekly; offered supervision, support. Control: monthly medical care only + scheduled evaluations.	Internally displaced mother-child dyads: Intervention group (n=42), Control group (n=45).	Intervention had positive effect on mothers' mental health, children's weight gain, children's psychosocial functioning, mental health (e.g. child's cognitive performance). However, no difference between intervention and control groups.	Yes
Punamäki et al., 2014 (23)	Gaza	RCT assessing psychosocial intervention in conflict-affected children. Intervention: Teaching Recovery Techniques, including CBT-based tools based, by 4 trained counsellors as 2x weekly 2 hour sessions over 4 weeks. Counsellors offered supervision. Sessions included navigating distress, unpleasant/numbed feelings, problem solving techniques, storytelling role plays. Control: waiting list.	Intervention group (n=242), Control group (n=240).	Intervention not effective at changing emotion regulation (ERQC) but general decrease in emotion regulation intensity, associated with reduced PTSD symptoms (CIES), depression symptoms (DSRSC), distress (SDS) and increased psychosocial well-being (MHCSF).	Yes
Gavrilova et al., 2009 (24)	Russia	RCT assessing caregiver intervention for people with dementia and their carers. Intervention: '10/66' 30 minute caregiver intervention, delivered over 5 weeks. Addressed assessment, basic dementia education, training on problem behaviours. Control: usual care from Alzheimer's disease and related disorders clinic.	Intervention group (n=30), Control group (n=30).	Intervention group caregivers had significantly improved burden (ZCBI) at 6 months, compared to control group. No differences in distress (SRQ-20), quality of life (DEMQOL and WHOQIL-BREF).	Yes
Bolton et al., 2014 (25)	Iraq	RCT assessing 2x culturally adapted interventions by trained, supervised CMHWs. Intervention 1: 12x BATD to plan and engage in positive activities daily. Engagement with activities chosen by participants is included in a structured programme and reinforced by the mental health professional. Intervention 2: 12x CPT, including cognitive restructuring, emotional processing of traumatic events. 20 CMHWs randomly assigned to intervention 1 or 2. Control: waiting list, advice to contact CMHWs if needed. Intervention by 5 months.	Survivors of systematic violence with depression symptoms: BATD (n=114), CPT (n=101), Control arm (n=66).	Estimated effect sizes for depression and dysfunction: 0.60 and 0.55, comparing BATD with all controls, 0.70 and 0.90 comparing CPT with all controls. BATD had significant effects on depression and dysfunction, CPT had only reduced dysfunction.	Yes
Weiss et al., 2015 (26)	Iraq	2x 2 arm RCTs of PHC interventions for survivors of systematic violence. Intervention 1: 8-12x weekly 50-60 minute CETA transdiagnostic sessions: psycho-education, relaxation, BA, cognitive coping, restructuring, imaginal and in vivo exposure, safety. CMHWs had 10-day training from 2 local supervisors + access to weekly Skype supervision. Control: waiting list. Intervention 2: CPT: cognitive restructuring, emotional processing of trauma-related content. CMHWs had 7 days' training, supervision. Control: waiting list.	RCT 1: CETA (n=99) Control (n=50)<br><br>RCT2: CPT (n=129) Control (n=64)	Larger effect sizes for CETA on all outcomes (trauma, depression, anxiety, dysfunction). CPT showed moderate effects sizes for trauma (HTQ) and depression, with small to no effect for anxiety (HSCD) or dysfunction (locally adapted scales).	Yes

Authors	Country	Study design	Sample	Findings	mhGAP-IG recommended intervention or approach?
Bass et al., 2016 (27)	Iraq (Kurdistan)	RCT assessing trauma-informed support, skills and psychoeducation by CMHWs.<br>Intervention: 6-12x time-limited trauma-informed support, skills, psychoeducation. CMHWs trained in 9 techniques with 4-6 activities + refresher training on clinical skills, emphatic reflection, building trust. Monthly on-site psychiatrist-led group supervision including notes monitoring + weekly phone check-ins.<br>Control: waiting list + monthly checks.	Intervention group (n=159), control group (n=50).	Post-intervention, statistically and clinically significant impact on depression (HSC-25) and dysfunction (Likert scale of tasks and activities), and significant but smaller impacts on anxiety (adapted HSC-25), vs. control.	Yes
Milani et al., 2013 (28)	Iran	RCT assessing telephone support for mild and moderate postnatal depression.<br>Intervention: 2-3x per week telephone support by health volunteers trained to facilitate communication about women's difficulties, until 6 weeks post-partum. Calls included health, newborn condition, relationship with newborn and partner.<br>Control: routine post-partum care.	Intervention group (n=22), Control group (n=24)	Depression scores of the intervention and control groups showed a significant difference post-intervention (measured using the Edinburg Postnatal Depression Scale), and these decreased significantly in both.	Yes
Meffert et al., 2014 (29)	Egypt	RCT assessing interpersonal psychotherapy for Sudanese refugees living in Cairo.<br>Intervention: 6x bi-weekly IPT delivered by Sudanese therapists, focused on improving relationships. Therapists received 1 week's training, offered formal group supervision bi-weekly and informal group supervision near-daily. Control: wait list.	Intervention group (n=13), Control group (n=9).	Significant decrease in PTSD symptoms (HTQ), state anger (STAI), depression (BDI-II) in intervention group.	Yes
Puffer et al., 2015 (30)	Liberia	RCT assessing Parents Make the Difference intervention for parents of 3-7 year olds.<br>Intervention: 10-session parent training + 1 home visit, on positive parenting, building cognitive and educational skills and malaria prevention. Delivered by pair of lay facilitators trained to lead weekly 2 hour sessions with 20-35 caregivers.<br>Control: waiting list.	Intervention group (n=135), Control group (n=135).	Significantly less self-rated harsh discipline, increased positive behaviour management, improved carer-child interactions, in intervention group. Child wellbeing, cognition, malaria unchanged.	Yes
Betancourt et al., 2014 (31)	Sierra Leone	RCT assessing CBT-based group intervention for symptomatic youth affected by war.<br>Intervention: 10-12x Youth Readiness Intervention, based on CBT and IPT. Included community/family meeting. Included: group cohesion, trauma psychoeducation, links between beliefs, bodies and behaviours, problem solving, relaxation, emotion regulation, BA, interpersonal skills, coping, self-perception, communication, choices, goals. Counsellors had 2 weeks' training, weekly supervision.<br>Post-intervention, education experience (EducAid) immediately or after waiting list. Comprised alternative school with funded textbooks and tuition.	Youth Readiness Intervention (n=222), Control group (n=214).<br><br>EducAid (n=220), Control group (n=216)	Intervention group participants: improved emotion regulation (DERS), prosociality (OMPA), social support (ISSB), functioning (WHODAS 2.0), school enrolment, attendance, classroom behaviour relative to control group. EducAid associated with better attendance but did not affect mental health, functioning, school retention, behaviour.	Yes

Omeje et al., 2018 (32)	Nigeria	RCT assessing rational emotive health therapy for alcohol use in HIV positive people.<br>Intervention: based on rational emotive and CBT.<br>Control: waiting list	Intervention group (n=61), Control group (n=63).	Significant reduction in alcohol use disorder symptoms (AUDS, AIBS) in intervention versus control group, maintained at 6 month follow-up.	Yes
Bass et al., 2013 (33)	Democratic Republic of Congo	Cluster RCT assessing CPT for female sexual violence survivors.<br>Intervention: 11x 2 hour group CPT + 1x 1 hour individual session, adapted to accommodate non-literate participants. Staff had 2 weeks' training, supervision.<br>Control: individual support from psychosocial assistants including financial, medical and legal referrals. Service monitored by supervisors reviewing monitoring forms.	Intervention group (7 villages, n=157), Control group (8 villages, n=248).	Improved depression, anxiety (HSC), PTSD (HTQ) and functional impairment in intervention versus control group. At 6 month follow-up, significantly fewer intervention women met criteria for probable depression, anxiety PTSD vs. control.	Yes
O'Callaghan et al., 2013 (34)	Democratic Republic of Congo	RCT of trauma-focused CBT for war-affected, sexually exploited 12-17 year-olds.<br>Intervention: 15x culturally modified, group trauma-focused CBT including trauma psychoeducation, stress management, coping, unhelpful cognitions. Separate parent/guardian sessions included trauma, children's rights.<br>Control: waiting list.	Intervention group (n=24), Control group (n=28).	Intervention participants showed significantly greater reductions in trauma (UPRR), depression, anxiety, conduct problems and increased prosocial behaviour (AYPAI) than control.	Yes
O'Callaghan et al., 2014 (35)	Democratic Republic of Congo	RCT assessing community-participative psychosocial intervention for war-affected.<br>Intervention: 8 session, thrice weekly 2 hour intervention for youth with 1 chosen caregiver. Included life skills leadership programme, mobile cinema clips for psychoeducation, relaxation technique scripts informed by trauma-focused CBT.<br>Control: waiting list.	Intervention group (n=79), Control group (n=80).	Intervention group: greater reductions in PTSD (IES) than control group. Large improvements in internalising symptoms, moderate improvements in prosocial scores (AYPAI) at 3 months. Caregivers reported conduct improvement.	Yes
Papas et al. 2011 (36)	Kenya	RCT of culturally adapted group paraprofessional CBT for HIV-infected outpatients.<br>Intervention: 6x 90 minute manualised gender-stratified group CBT. Treatment by 2 trained counsellors. Group sessions videotaped and monitored.<br>Control: routine medical care provided in clinic.	Antiretroviral-initiated/eligible outpatients with hazardous or binge drinking (n=75)	Large effect sizes of alcohol use reduction since baseline at 30 day follow-up in intervention versus control group. Abstinence at 90 days: 69% in intervention group, 38% in control group.	Yes
Alexander et al., 2013 (37)	Kenya	RCT assessing private practitioner training to recognise depression.<br>Intervention: 1 hour presentation on National Institute of Mental Health publication on recognising depression + demonstration of PHQ-9 + PHQ-2.<br>Control: waiting list.	Intervention group (n=22), Control group (n=22)	Intervention group more comfortable diagnosing depression (KPCPQ), favoured medication. Barriers: resources, resistance/cooperation, training, communication, cultural beliefs.	N/A (training)

Jenkins et al., 2013 (38)	Kenya	Pragmatic cluster RCT assessing training + national health sector reforms for mental disorder detection and recovery.<br>Intervention: 40 hour 5 module course in 30-60 minute sessions, including overview of core mental health concepts, skills, symptoms, assessment, diagnosis, management, through theory, discussion and role play.<br>Control: routine care.	33 dispensaries + 16 health centres per arm. 98 staff trained (2 per intervention clinic). 468 + 478 had 3 month follow-up.	Accurate routine detection of mental disorder greater in intervention than control group at 3 months. SMD in client improvement using intention-to-treat analysis: GHQ: 0.34, EQ5D: 0.39, WHODAS: 0.49.	N/A (training)
L'Engle et al., 2014 (39)	Kenya	RCT assessing impact of brief intervention on alcohol use, sexually transmitted infection incidence, sexual risk behaviours of moderate drinking female sex workers.<br>Intervention: 6x 20 minute monthly counselling sessions from nurses trained in motivational interviewing. Women received AUDIT results, discussed alcohol risks and consequences, set goals for reducing consumption, breaking habits, coping.<br>Control: 6x monthly sessions about nutrition and HIV. Nutritional assessment, nutritional needs addressed, nutrition care plan for women and their children.	Intervention group (n=410), Control group (n=408)	Significant reduction in alcohol use and binge drinking at 6 and 12 months. Intervention did not impact STI incidence, self-reported condom use or intimate partner sexual violence. Sexual violence from clients significantly lower among intervention women at 6 and 12 months in comparison to control group.	Yes
Bolton et al., 2003 (40)	Uganda	Cluster RCT of group interpersonal psychotherapy (IPT).<br>Intervention: 16x weekly 90 minute group sessions (5-8 participants) led by same sex trainers who received 2 weeks' training from study authors. Group leader reviewed depression symptoms and encouraged participants to share events from the previous week that may have influenced mood.<br>Control: treatment as usual.	248 participants with depression (HSCL) & dysfunction. Inter-vention group (n=15 villages), Control group (n=15).	Significantly greater mean reduction in depression severity and dysfunction in intervention relative to control group. Post-participation, 7% of intervention and 55% control participants were still depressed.	Yes
Bass et al., 2006 (41)	Uganda	RCT of group IPT at 6 months' follow-up.<br>Intervention: as above. Control: as above.	Intervention (n=103), Control (n=113)	Intervention: lower depression symptoms and diagnoses, functional impairment at 6 months.	Yes
Bolton et al., 2007 (42)	Uganda	RCT for depression, anxiety, conduct problems of adolescent survivors of war.<br>Intervention 1: 16x weekly 1.5-2 hour manualised IPT adapted for youths, delivered in groups of 6-8 boys or girls. Facilitators received weekly supervision.<br>Intervention 2: 16x weekly 1.5-2 hour creative play therapy to strengthen resilience through creative expression (art, role play, music, sport) in groups of 25-30.<br>Control: waiting list.	Intervention 1 (n=105 in 12 groups), Intervention 2 (n=105 in 4 groups), Control group (n=105)	Girls but not boys receiving group IPT showed significant improvement in depression symptoms compared with control. Creative play showed no effect on depression. Neither intervention affected anxiety, conduct or functioning.	Yes

Neuner et al., 2008 (43)	Uganda	RCT training 9 lay counsellors in refugee settlement for 6 weeks in general counselling skills including active listening, empathy and specific interventions.<br>Intervention 1: 6x up to 2 hour, biweekly strict manualized narrative exposure therapy (NET): participant constructs detailed chronological autobiography with counsellor, transforming fragmented traumatic experiences into coherent narrative.<br>Intervention 2: 6x up to 2 hour, biweekly flexible trauma counselling.<br>Control: no treatment.	Rwandan and Somalian refugees diagnosed with PTSD. NET (n=111), Trauma counselling (n=111), Control group (n=55).	Fewer participants dropped out of NET than trauma counselling. Both active groups statistically, clinically superior to control for PTSD (PTSDS), physical symptoms (6 illness checklist) but not from each other. At follow-up, PTSD no longer diagnosed in 70% of NET and 65% trauma counselling but only 37% of control participants.	Yes
Ertl et al., 2011 (44)	Uganda	RCT of community intervention for PTSD in former child soldiers.<br>Intervention 1: 8 individual at-home sessions of NET (as above) in internally displaced persons camps.<br>Intervention 2: Academic catch-up programme with supportive counselling, including intensive English catch-up course.<br>Control: waiting list.	85 former child soldiers with PSTD from population survey. NET (n=29), Catch-up (n=28), Control (n=28).	PTSD symptom severity (PTSDS) significantly improved in NET versus academic catch-up and waiting list groups. NET superior to academic catch-up and waiting list for PTSD symptom change over time. Larger within-treatment effect of NET than academic catch-up and waiting list.	In part
Wagner et al., 2017 (45)	Uganda	Cluster RCT of task-shifted, protocolised antidepressant use in HIV clinics.<br>Protocolised model: algorithm-based, nurse-driven approach to diagnosing depression and prescribing antidepressants (staff received 1 day intensive training).<br>Clinical model: brief routine depression screening at each clinic visit by PHC staff followed by response at staff discretion (staff received 1 day's intensive training).	People screening as depressed across 10 HIV clinics (n=1028), followed for 1 year. Staff: 2 years.	Depression alleviation was associated with better antiretroviral therapy (ART) adherence and clinic attendance at 12 months' follow-up. The association was mediated by self-efficacy at month 12.	Yes
Richards et al., 2014 (46)	Uganda	RCT assessing sport-for-development programme for fitness and mental health.<br>Intervention: 11 week football league, joined voluntarily by 11-14 year olds.<br>Control: waiting list (boys only: all girls allocated to intervention due to low numbers<br>Comparison group: Non-subscribers formed a non-registered comparison group.	Intervention group (n=74 boys + 81 girls), Control (72 boys), Comparison (472 boys, 763 girls)	Lower depression-like and anxiety-like symptoms but no significant effect on cardiorespiratory fitness, muscular power, body composition of boys at 4 months compared to either control. No significant effect on girls' physical/mental health.	Yes

Singla et al., 2015 (47)	Uganda	Cluster RCT of parenting intervention for child development, maternal wellbeing. <u>Intervention:</u> 60-90 minute manualised parenting intervention for mothers and fathers, supplemented with 40-50 minute home visits by volunteers who had received 14 days' training, to review content. 5 messages on childcare (play, talk, diet, hygiene, love, respect), cognitive, behavioural, interpersonal, stimulation interventions and increasing father involvement. Volunteers were supervised. <u>Control:</u> Waiting list + brief messages on nutrition (e.g. coloured poster).	Intervention group (13 communities in 6 parishes (n=194 child-mother dyads), Control (12 in 6 parishes (154 dyads).	Intervention group children showed significantly higher cognitive scores and receptive language scores (BSID-3) than control group children. Intervention group mothers showed significantly lower depressive symptoms (CES-D) than control group mothers. No differences in child growth.	Yes
Kaaya et al., 2013 (48)	Tanzania	RCT of group problem-solving therapy for HIV positive pregnant women. <u>Intervention:</u> 6x weekly group facilitated by trained social worker or psychiatric nurse on challenges, whom to disclose to, prevention, transmission, home and community support, future planning, accessing services, psychosocial support. <u>Control:</u> standard care (voluntary counselling and testing for HIV) + information on services (did not typically offer social support group counselling).	HIV positive pregnant women attending antenatal clinics: Intervention group (n=168), Control group (n=163)	Intervention group women had lower depressive symptoms than control group women. Intervention did not impact disclosure rates but personal satisfaction with disclosure was higher in the intervention than the control group.	Yes
Connolly & Sakai, 2011 (49)	Rwanda	RCT of Thought Field Therapy for PTSD in survivors of the Rwandan genocide. <u>Intervention:</u> self-help: participant rates emotional intensity from 0 to 10, supervised Rwandan therapist trained for 2 days selects acupoint on skin to tap while mentally activating the memory/trigger. Also, eye movements, bilateral stimulation, counting. Repeated until emotional intensity lowest possible. <u>Control:</u> waiting list.	Intervention group (n=71), Control group (n=74).	Intervention associated with significant reductions in 9 out of 10 TSI subscales, severity and frequency on modified PTSDSS. Reduced trauma symptoms maintained 2 later.	In part
Connolly et al., 2013 (50)	Rwanda	RCT of Thought Field Therapy for PTSD in survivors of the Rwandan genocide. <u>Intervention:</u> see above. <u>Control: waiting list.</u>	Intervention group (n=85), Control group (n=79).	Significant difference in trauma (TSI) and PTSD symptom severity and frequency (modified PTSDSS) between groups. Control showed significant reductions in trauma.	In part
Yeomans et al., 2010 (51)	Burundi	RCT of trauma healing and reconciliation intervention for PTSD. <u>Intervention 1:</u> 3 day workshop + follow-up session 1 month later: discussion and exercises for interpersonal exchange, games exploring trauma, loss, anger, trust, roots of violence. Psychoeducation: 90 minute presentation on 17 symptoms of PTSD. Relaxation exercises and emphasis on repairing relationships. <u>Intervention 2</u>: workshop without psychoeducation. Pair activity on trust, safety. <u>Control:</u> waiting list.	Intervention 1 (n=41) Intervention 2 (n=41) Control group (n=40).	Both interventions reduced PTSD symptoms (HSCL-25) compared to control. Intervention 2 participants showed greater PTSD symptom reduction than Intervention 1.	Yes

Kauye et al., 2014 (52)	Malawi	Cluster RCT of mental health toolkit for PHC staff.<br>Intervention: 5 day training in 30 minute sessions, including mental health and illness, basic psychosocial skills, common neurological disorders, psychiatric disorders, health and other sector policy issues, legislation, links between mental and child health, reproductive health, HIV and malaria, roles and responsibilities, working with CHWs and traditional healers, integrating mental health into annual operational plans. Sessions included theory, group discussion and role-play.<br>Control: 3 day existing mental health training. Content included psychiatric illness, including secondary to physical health problems.	Intervention group (n=1373 over 9 practices), Control group (n=1356 over 9 practices).	Intervention associated with increased diagnosis of depression and anxiety, control group associated with increased diagnosis of malaria.	N/A (training)
Murray et al., 2015 (53)	Zambia	RCT of supervised lay counsellor trauma-focused CBT for vulnerable children.<br>Intervention: 10-16 weekly 60-90 minute sessions for youths and caregivers: psychoeducation, parenting, relaxation, affective modulation, coping, trauma narrative, exposure, joint session, safety skills. Lay counsellors had 10 day training.<br>Control: treatment as usual, including psychosocial counselling, home-based care. Telephoned weekly, visited monthly to review safety, assess need for referral.	5-18 year-olds who had experienced trauma with trauma-related symptoms. Intervention (n=131), Control (n=126).	Intervention group had greater mean item change in trauma symptom score (PTSDRI) than control group at follow-up. Intervention group: greater mean item change in functioning (locally developed scales) than control at follow-up.	Yes
Chibanda et al., 2014 (54)	Zimbabwe	RCT of peer counselling for postpartum mothers.<br>Intervention: 12x 1 hour biweekly group problem-solving therapy. Supervised counsellors attended 2 days' training on depression, treatment, communication skills, suicide risk, involving family, exploring resources, support systems, follow-up.<br>Control: amitriptyline prescribed by PHC nurse with weekly review + information about breastfeeding.	Intervention group (n=30), Control group (n=28)	Greater reduction in mean depression symptoms (EPDS) at 6 week follow-up among intervention than control group women.	Yes
Chibanda et al., 2016 (55)	Zimbabwe	Cluster RCT of culturally adapted psychological intervention by lay workers in PHC.<br>Intervention: 6x individual problem-solving therapy (Friendship Bench) sessions by trained lay health workers supervised by senior health promotion officers. Manualised 9 day training included detailed script. Participants reminded about PST during intervention, reinforced, encouraged. Optional 6-session peer support.<br>Control: standard care plus information, education, support on CMDs.	intervention group (n=286 across 12 clinics), Control group (n=287 across 12 clinics).	Intervention group participants reported fewer CMD symptoms (SSQ-14) and depression symptoms than control group participants.	Yes
Igreja et al., 2004 (56)	Mozam-bique	RCT of testimony method for PTSD symptoms.<br>Intervention: 1x 60 minute session: writing testimony notes, read back by interviewer to produce accurate final version. Topics: 1 major traumatic experience, facts around event; the individual and social aspects, perceptions, feelings, future.<br>Control: no intervention.	Intervention group (n=66), Control group (n=71)	Significant symptom reduction in both groups: no significant differences between intervention and control groups. Follow-up: sustained lower symptoms in both groups, possible positive intervention effect in women.	No

Wainberg et al., 2018 (57)	Mozam-bique	Trial registration: Community I-STAR (Implementation of SBIRT (Screening, Brief Intervention, Referral to Treatment) using Technology for Alcohol use Reduction: intervention adaptation, cluster RCT, scale-up. CHWs in 12 districts randomised to mSBIRT or SBIRT Conventional Training and Supervision strategy (SBIRT-CTS).	540 people diagnosed with hazardous drinking	(1) Formative adaptation of mobile mSBIRT app. (2) 2-year cluster-randomised, hybrid effectiveness-implementation trial in 12 districts. (3) More cost-effective arm scaled up.	Yes
Cooper et al., 2009 (58)	South Africa	RCT of lay community worker home visits for mother-infant attachment/bonding. Intervention: 16x 1 hour home sessions: 2x antenatal, weekly for 8 weeks postpartum, fortnightly for 2 months then monthly for 2 months. 'The Social Baby' adaptation. Delivered by 4 women trained over 4 months in basic parenting and counselling skills + specific mother-infant intervention. Supervised by psychologist. Control: local infant clinic usual care including fortnightly mother-child CHW visits.	Intervention group (n=22), Control group (n=220).	Intervention group women significantly more sensitive and less intrusive infant interactions at 6 and 12 months than control group + more secure infant attachment (strange situation procedure) at 18 months. Intervention associated with improved mood (EPDS) at 6 months.	Yes
Rendall-Mkosi et al., 2013 (59)	South Africa	RCT of motivational interviewing to reduce alcohol-exposed pregnancies. Intervention: 5x motivational interviewing sessions: 1) building rapport and setting future agenda, 2) assessing readiness to change and confidence in behaviour change 3) behavioural change plan, 4) implementing plan, assessing challenge/problem-solving, 5) reviewing counselling experience, reinforcing follow-up/after care plan. Control: health handbook (also provided to women in the intervention group).	Intervention group (n=82), Control group (n=83) + life skills component only (n=31).	Intervention group: significant reduction in proportion of women at risk of alcohol-exposed pregnancy at 3 months, maintained at 12 months, compared to control group.	Yes
Peltzer et al., 2013 (60)	South Africa	Cluster RCT of brief interventions to reduce alcohol intake in patients with active TB. Intervention: brief counselling for alcohol reduction: risk information, motivation to change assessment, behavioural skills (adopting preventative). Counsellors trained to give brief counselling alongside short-course medication and supervised at start. Control: health education leaflet on responsible drinking.	PHC centres with highest TB caseload: intervention group (n=7), control group (n=7).	No statistically significant effect (AUDIT), including for hazardous/harmful, dependent or heavy episodic drinking (AUDIT). There was a significant improvement in hazardous drinking in the control group.	Yes
Le Roux et al., 2014 (61)	South Africa	Cluster RCT of CHW home visits for infant well-being from pregnancy. Intervention: Philani Intervention Programme + usual care + postnatal CHW home visits. CHWs trained for 1 month using manual, role-play, challenging situations on home visits videos. Antenatal messages on regular attendance, danger signs, maternal nutrition, preparing to breastfeed, HIV testing, stopping alcohol. Postnatal messages on breastfeeding, growth monitoring, medical adherence, bonding, securing child grants. Control: standard care/government clinic/hospital care, rapid HIV test and results.	Intervention group (n=500 women across 12 neighbourhoods), Control group (n=644 in 12 neighbourhoods)	Intervention group women: significant overall benefits compared to control. More vertical transmission prevention, exclusive breast feeding for 6 months, birth complication prevention, higher height-for-age than control group.	Yes

Mertens et al., 2014 (62)	South Africa	RCT of brief motivational intervention for alcohol and drug use in young adults.<br>Intervention: Brief Motivational Interviewing by PHC nurses trained for 3 days then regularly supervised by trainer who provided feedback on audio recordings to maintain fidelity with the model. Recordings scored using BCCI. Participants received resource list for substance use problems.<br>Control: usual care, a handout and resource list for substance use problems.	Intervention group (n=206), Control group (n=197).	No difference in at-risk alcohol and drug use. Intervention group participants had significantly lower ASSIST alcohol scores.	Yes
Mutyambizi-Mafunda et al., 2019 (63)	South Africa	Protocol: 3 arm cluster RCT of task-sharing to integrate a 3-4 session motivational interviewing/problem solving intervention into PHC for HIV and diabetes:<br>1) Intervention 1 (dedicated approach): dedicated CHWs whose sole task is to delivering a new counselling service, with supervision.<br>2) Intervention 2 (designated approach): training existing CHWs to deliver counselling alongside chronic disease care, with supervision.<br>2) Control group: typical PHC CMD treatment – mostly referral to other services.	1200 patients (600 with HIV and 600 with diabetes) across 24 public PHC facilities.	Outcomes at baseline, 6, 12 months' follow-up:<br>Primary: depression (CES-D), alcohol use (AUDIT).<br>Secondary: HbA1c if diabetes, HIV-1 RNA viral load, adherence, quality of life (EQ-5D).<br>Cost-effectiveness: costs per unit reduction of AUDIT, CES-D, HIV viral load, HbA1c per year.<br>Cost utility analysis: cost per QALY gained.<br>Budget impact analysis: business case for scaling up investment in mental health services.	Yes
Ali et al., 2003 (64)	Pakistan	RCT of counselling by 21 women who received 11 sessions of training.<br>Intervention: 8x weekly counselling sessions. Counsellors trained on anxiety/ depression, stress/anger management, communication/counselling skills. Women received supportive, cognitive, problem-solving counselling at home.<br>Control: No intervention.	366 anxious and/or depressed women	Intervention group women had significantly reduced mean anxiety and depression scores (AKUADS) compared to control group.	Yes
Gul & Ali, 2004 (65)	Pakistan	RCT of counselling by community counsellors for anxiety and depression in women.<br>Intervention: 8x 1 hour weekly counselling. Community women trained 3x a week for 4 weeks, on mental health, communication, counselling, simple CBT techniques.<br>Control: was offered counselling after trial.	Intervention group (n= 82), Control group (n=91).	Both groups showed significant reduction in mean anxiety and depression scores after 4 weeks, further improved at 8 weeks.	Yes
Rahman et al., 2009 (66)	Pakistan	Cluster RCT of parent programme for infant development, maternal mental distress.<br>Intervention: Learning Through Play programme to help women read infant cues and develop sensitive responsiveness through play. Women received calendar at half-day workshop in second month of pregnancy, then fortnightly home visits. 1 of 24 lay health workers discussed child's development for 15-20 minutes.<br>Control: routine follow-up visits. Both groups reassessed postnatally.	Intervention group (n=163 women across 24 villages), Control group (n=146 across 24 villages).	Intervention group women showed significant increases in knowledge and attitudes to infant development compared to control group. No difference in mental distress (measured with the (SRQ-20) between groups.	Yes

Rahman et al., 2008 (67)	Pakistan	Cluster RCT of CBT-based intervention on maternal depression and infant outcomes. <u>Intervention:</u> PHC staff trained to deliver Thinking Healthy Programme: active listening, family collaboration, guided discovery, homework. Integrated into routine maternal and child education. <u>Control:</u> untrained health workers made equal number of visits to women.	Intervention group (n=463 women across 20 Union council clusters), Control group (n=440 women across 20 clusters).	Lower depression prevalence, depression scores, disability + higher functioning, social support, contraception, breastfeeding in intervention than control group at 6 + 12 months. Intervention group infants less past-two weeks diarrhoea, more likely to be vaccinated. Intervention parents played with infants longer than control.	Yes
Hirani et al., 2010 (68)	Pakistan	3 arm RCT of Economic Skill Building or counselling by CHWs trained for 21 hours. <u>Intervention 1:</u> 8x weekly programme developed using participatory methods: employment skills including communication, work-life balance, time management, conflict resolution, dealing with abuse, self-efficacy, parenting, personal hygiene. <u>Intervention 2</u>: Group counselling on stress and anger management, effective communication, active listening, supportive problem-solving. <u>Control:</u> no intervention.	24 economically disadvantaged women. Intervention 1 (n=9), Intervention 2 (n=7), Control group (n=8).	Depression (BDI-II) and abuse (WHO instrument) lowest among women who received Intervention 1 but difference not statistically significant. Intervention 1 women reported significantly higher self-efficacy (GSE), more employment than Intervention 2 and control.	In part
Rahman et al., 2016 (69)	Pakistan	Randomised feasibility study of PM+ brief transdiagnostic psychological intervention using problem-solving, behavioural activation, social support, stress management. <u>Intervention:</u> PM+ adapted for conflict-affected Peshawar. 5 sessions by supervised non-specialists trained over 6 days by a master trainer. Staff practiced for 4 weeks under supervision. <u>Control:</u> enhanced treatment as usual: 1 day CMD treatment training for PHC Drs.	People referred by PHC physician due to distress, impairment. PM+ (n=30) Control (n=30).	Intervention improved functioning (WHODAS 2.0 scores reduced by 11.1 vs 5.7 in control arm) and PTSD symptoms (PCL-5 reduced by 24.4 vs 12.8 in control arm). No GHQ-12 difference.	Yes
Rahman et al., 2016 (70)	Pakistan	RCT of multicomponent behavioural intervention by lay health workers in PHC. <u>Intervention:</u> 5x weekly 90 minute PM+, focused on supported problem-solving, behavioural activation, strengthening social support, stress management. Lay health workers received 8-days' training from trained master trainers, on CMDs, basic counselling, intervention strategies, self-care. Lay health workers received weekly group supervision including fidelity check, from supervised in-country supervisors. <u>Control:</u> enhanced usual care: at least 1 contact with PHC physician who attended 5 days' training + 1 day anxiety, depression, psychoeducation, counselling refresher.	Intervention group (n=177), Control group (n=174)	Intervention group had significantly lower HADS anxiety and depression, PCL-5, functional impairment (WHODAS 2.0), depression symptoms (PHQ-9) and problems for which they sought (Psychological Outcome Profiles) than control group at 3-months post-intervention.	Yes

Rahman et al., 2019 (71)	Pakistan	Cluster RCT of brief group psychological intervention for conflict-affected women.<br>Intervention 5x weekly group PM+ facilitated by local women in the homes of LHWs: psychoeducation, goal setting, brief motivational interviewing, stress management, problem solving, behavioural activation, strengthening social support. Locally relevant pictorial materials about women's case narratives for non-literate participants. Facilitators received 7 days' training by master trainer.<br>Control: feedback on assessment results, psychoeducation for participants, family members, chance to talk to LHW who received half day training on psychoeducation supportive communication, options for seeking care for distress. PHC providers received half day training in the detection and management of mental health problems and referral pathways.	Intervention group (n=306 across 17 community clusters), Control group (n=306 across 17 community clusters)	Intervention group women had significantly lower mean total, anxiety and depression scores on HADS than control group women at 3 months follow-up. They also had significantly improved functional impairment (WHODAS), problems for which they sought help (PSYCHLOPS) and depressive disorder symptoms (PHQ-9).	Yes
Hamdani et al. 2021 (72)	Pakistan	RCT of effectiveness and cost-effectiveness of PM+ for CMDs.<br>Intervention: 5x weekly sessions by trained therapists who received 8 days' training from master training, plus TAU.<br>Control: TAU: initial evaluation by trainee psychologists and psychiatrists followed by expert consultation for pharmacotherapy.	Adults: psychological distress, impaired functioning .Intervention (n=96), Control (n=96)	Intervention group participants showed significant improvement in HADS anxiety and depression and functioning than the control group at 20 weeks' follow-up. 67% of participants were followed up.	Yes
Rahman et al., 2016 (73)	India, Pakistan	Randomised feasibility study of parent-mediated intervention.<br>Intervention: individual home sessions between health worker and parent, with child with autism spectrum disorder (ASD) present + TAU. Aim: increase parental response, reduce over-directive parental responses, develop child's communication.<br>Control: TAU from local facilities.	Intervention group (n=32 children with ASD), Control group (n=33)	Intervention group showed improved parent-child interaction and initiation of communication by child, relative to control group. No improvement in shared attention. No difference between arms in child adaptation and language, child language use, social communication.	Yes
Patel et al., 2003 (74)	India	RCT of antidepressant and psychological treatment for CMDs in PHC settings.<br>Intervention 1: antidepressant treatment (fluoxetine) for up to 6 months.<br>Intervention 2: up to 6x psychological intervention sessions by trained therapists, covering treatment explanation and reassurance, relaxation (breathing exercises), treatment for specific symptoms, problem-solving.<br>Control: placebo for up to 6 months.	Intervention 1 (n= 150), Intervention 2 (n=150), Control group (n=150).	Psychiatric morbidity (RCIS) significantly better with antidepressant than placebo at 2 months follow-up but not maintained between 2-12 months. Antidepressants significantly more cost effective than placebo in short and long-term. Psychological treatment not more effective than placebo for morbidity, disability (BDQ), costs.	Yes

Pal et al., 2007 (75)	India	RCT of brief intervention for alcohol use based on FRAMES motivational interview<br>Intervention: 2x 45 minute sessions. Session 1: feedback on harmful consequences of drinking, alternatives. Session 2: alternative activities/highs, evaluating risks.<br>Control: 5 minute session with empathic expression of concern from facilitator + advice to cut down/stop drinking alcohol.	Intervention group (n=45), Control group (n=45).	Intervention group showed less severe past-30 day alcohol use, composite ASI scores, improved quality of life (WHOQoL) than control group. Motivation toward action (Hindi RCQ) at 1 month follow-up not sustained in any group.	Yes
Dias et al., 2008 (76)	India	RCT of home intervention for caregivers of elderly people with dementia.<br>Intervention: 2x full-time home care advisors + 1 part-time local psychiatrist from public health service + 1 part-time lay counsellor, supervised by specialist. Team received 1 week role play-based, interactive training on listening, counselling, bereavement counselling, stress management, common health problem advice. Aim: support caregiver through information on dementia, provide guidance on behavioural management + medication if needed, following psychiatric assessment.<br>Control: waiting list + education and information about dementia.	Intervention group (n=41 families), control group (n=40).	Intervention associated with significantly better mental health (GHQ) and distress due to behavioural disturbance (NPI-D) than control at follow-up. No significant reductions in caregiver burden (ZCBI), activities of daily living (EASI) and behavioural problems (NPI-S) in person with dementia. Non-significant reduction in total deaths in intervention arm.	Yes
Tripathy et al., 2010 (77)	India	Cluster RCT of community mobilisation through participatory women's groups.<br>Intervention: 20x women's group meetings facilitated by local women who attended a residential training course on intervention content and participatory communication. Meetings covered clean delivery practices, help-seeking through stories, role-plays, games. Intervention group also had access to control.<br>Control: health committees on local services. ~10 village representatives met every 2 months. 7 clusters per district allocated workshops with government health staff.	Intervention group (n=9770 births across 18 clusters), Control group (n=9260 births across 18 clusters).	Neonatal morality rate lower over 3 years' follow-up in intervention clusters. No difference in depression (Kessler-10) between intervention and control but reduced moderate depression in intervention year 3.	In part
Patel et al., 2010 (78)	India	Cluster RCT of lay health counsellor PHC intervention for depression and anxiety.<br>Intervention: 6-12 sessions of psychoeducation, interpersonal psychotherapy in a stepped care model. Lay health counsellors attended 2 month training then case-managed all participants screening (ICD-10) positive for CMDs, delivering all non-drug treatments in partnership with PHC physician and visiting psychiatrist.<br>Control: Physicians, patients received screening results and treatment manual.	Intervention group (n=1360 across 12 clusters), Control group (n=1436 across 12 clusters	Intervention group participants more likely to recover from CMDs at 6 months than control group. Strong intervention effect for participants from public facilities but not private facilities. 3 deaths, 4 sucide attempts in intervention group, 6 deaths, 6 suicide attempts in control group.	Yes

Authors	Country	Study design	Sample	Findings	mhGAP-IG recommended intervention or approach?
Chowdhary et al., 2016 (79)	India	Pilot randomised feasibility study of brief psychological treatment for severe depression delivered by lay counsellors in PHC. Supervisors (n=12) and patients (n=30) interviewed afterwards.<br>Intervention: 6-8x Healthy Activity Programme (HAP) sessions comprising behavioural activation, problem-solving, activation of social networks.	PHC attendees scoring > 14 on PHQ-9. Intervention group (n=24), Control group (n=31).	Depression prevalence (BDI-II) lower in HAP than the control arm at 2 month follow-up. HAP was modified to enhance contextual acceptability and feasibility for delivery by lay counsellors (e.g. home-based delivery, involving significant others in sessions, appropriate language and metaphors checklists for counsellors, simplifying content).	Yes
Fuhr et al., 2019 (80)	India	RCT of peer-delivered Thinking Health Programme (THP).<br>Intervention: 6-14x individual peer-delivered simple CBT at convenient location.<br>Control: Enhanced usual care: communicating screening results to women via information sheet on self-care for mental health and to obstetrician, with mhGAP depression guideline and referral guidance.	Intervention group (n=140), Control group (n=140)	Higher depression remission at 6 months post-natal in intervention group compared with control group but no difference in symptom severity between groups. No significant group differences in serious adverse events.	Yes
Muke et al., 2020 (81)	India	3 arm RCT of digital training for non-specialist health workers to deliver brief psychological treatment for depression. All arms trained staff to deliver HAP brief psychological treatment for depression in PHC. Comprises 2 manuals on general counselling skills and treatment-specific skills.<br>Intervention 1: digital training in 16 modules via Moodle learning management system, accessible through a smartphone app, alone, with technical support.<br>Intervention 2: digital training with remote support (weekly phone call).<br>Control: conventional 6 day classroom training: 16 modules by 2 master trainers.	42 staff randomised, 36 started training: Intervention 1 (n=12), Intervention 2 (n=14), Control (n=10) Focus groups (n=28).	Remote support improved digital training completion rates. No between-group differences in post-training competence assessed with short clinical vignettes and multiple-choice questions. However, greater improvement in intervention 2 and control than intervention 1. Focus groups identified issues e.g. poor connectivity, the app not loading, difficulty navigating course content.	Yes

Wijesinghe et al., 2015 (82)	Sri Lanka	RCT of brief intervention to reduce post-snakebite psychological morbidity in men. Intervention 1: 15 minute structured discussion with doctor about causes, consequences and options post-snakebite at hospital discharge, based on PFA. Intervention 2: intervention 1 plus 20 minute trauma-informed CBT session 6 months post-discharge. Doctors followed checklist to assess cognitions on health, personal life, functional abilities, future expectations, maladaptive behaviours. Control: no intervention.	Intervention 1 (n=65), Intervention 2 (n=69), Control group (n=68).	No difference between groups in proportion of patients diagnosed with depression at 6 months. Decreasing trend in proportion with depression and anxiety symptoms (HSCL-25, modified Sinhala BDI, SDI, PTSSS) in intervention compared to control groups. Decreasing trend in overall disability between intervention + control groups.	Yes
Puvimanasinghe & Price, 2016 (83)	Sri Lanka	RCT of testimonial therapy for Sri Lanka survivors of torture and ill-treatment. Intervention: 60-90 minute sharing of distressing event: emotions, cognitions, bodily sensations, short mindfulness session. Testimonials recorded and read aloud, for participant editing. Final testimonial presented at ceremony 4-6 weeks later. Control: waiting list.	Survivors of torture and ill-treatment. Intervention (n=13), Control (n=13).	Psychosocial functioning improved significantly in the 2-3 months post-intervention, compared to control group.	No
Sangraula et al., 2020 (84)	Nepal	Cluster randomised feasibility trial of locally adapted group PM+. Intervention: 5x 2.5-3 hour group sessions delivered by community psychosocial workers. Topics included problem and stress management, behavioural activation, strengthening social support. Volunteer local helpers organised logistics. Control: follow-up, psychoeducation, focus on support, mental health service details from staff trained for 10 days to identify, assess, treat CMDs using mhGAP-HIG.	Screening positive for distress: Intervention group (n=61), Control group (n=60). Interviews with key informants (n=25)	Non-specialist-delivered group PM+ was acceptable and feasible. Although the study was not powered to assess effectiveness, WHODAS, GHQ-12, PCL-5, RTC and PHQ-9 scores all improved more in the intervention arm than the control arm.	Yes
Li et al., 1989 (85)	China	Feasibility study of WHO community epilepsy care in rural Beijing and Sichuan PHC. Intervention: treatment with phenobarbital by 3 days' trained PHC staff. Control: treatment by neurologists.	Intervention (n=20), Control group (n=20)	No difference in adherence or treatment response between groups. PHC management was feasible.	Yes
Gao et al., 2010 (86)	China	RCT of IPT-orientated childbirth psychoeducation for pregnant women. Intervention: 2x 2 hour IPT-orientated group childbirth psychoeducation conducted by midwives + usual antenatal care (ANC). Aimed to address concerns and interpersonal problems post-partum. Telephone follow-up post-partum. Included information, clarification, communication analysis, role play, brainstorming. Control: 2x 90 minute sessions of labour and childcare education from midwives.	Intervention group (n=96), Control group (n=98).	Intervention group women had significantly fewer depressive symptoms (EPDS), better psychological well-being (GHQ), interpersonal relationships (SIRS) than control group women 6 weeks post-partum.	Yes

Gao et al., 2012 (87)	China	Follow-up RCT of IPT-orientated childbirth education 3 months post-partum.<br>Intervention: See Gao et al., 2010, above.<br>Control: See Gao et al., 2010, above.	Intervention group (n=96), Control group (n=98).	Intervention group women: significantly more improved perceived social support (PSSS), maternal role competence (PSCS efficacy subscale), postpartum depression (EPDS) + psychological well-being (GHQ) than control at 3 months post-partum.	Yes
Tiwari et al., 2010 (88)	China	RCT of advocacy intervention for women survivors of intimate partner violence (IPV)<br>Intervention: advocacy intervention and telephone social support, over 12 weeks by 5 day-trained research assistants. Aim: to empower women, link them with community services, support, informal counselling. Also access to usual care services from the community centre/outreach sites.<br>Control: usual community services (childcare, healthcare and promotion, recreational programmes) not designed specifically for women experiencing IPV.	Intervention group (n=100), Control group (n=100).	No clinically significant difference in depressive symptoms (Chinese BDI II) at 3 and 9 months. Significant improvement in partner psychological aggression (CTS-R), social support (ISEL) at 3 and 9 months but not physical assault, sexual coercion or health-related quality of life (SF-12). Women found intervention useful for improving intimate relationships, resolving conflicts.	Yes
Xu et al., 2019 (89)	China	RCT of mobile texting for schizophrenia care versus free medicines programme.<br>Intervention: lay health supporter + text message medication reminders, health education, monitoring for early relapse signs, facilitated linkage to PHC + control.<br>Control: nationwide community mental health programme of free antipsychotics.	Intervention group (n=139), Control group (n=139).	Medication adherence higher in intervention than control group. Reduced relapse risk in intervention (26/120) versus control (40/117) group and re-hospitalisation: (9/123 versus 25/122. No statistical difference in other outcomes (functioning, symptom severity).	Yes
Schwank et al., 2020 (90)	China	Protocol: multicentre RCT of web-based perinatal psychosocial peer support.<br>Intervention: THP for identification and management of perinatal distress, depression, anxiety symptoms in non-specialised settings + standard perinatal care. Trained peers will apply strategies during work, via WhatsApp and WeChat apps.<br>Control: standard perinatal care at maternity clinics.	Women living in large metropolitan cities between 12-18 weeks of pregnancy gestation.	Aims: to investigate impact on:<br>1) EPDS in pregnancy.<br>2) Maternal anxiety (GAD-7),<br>3) maternal stress (IES-R) during COVID-19.<br>4) mental health, pregnancy outcomes<br>5) elective caesarean at woman's request rates.	Yes

Chen et al., 2000 (91)	Taiwan	RCT of weekly supportive group meetings for women with postnatal distress.<br>Intervention: 4x weekly 1.5-2 hour support groups of 5-6 mothers with 6-10 week-old infants, facilitated by registered nurse (2 groups had additional 5 sessions). Aim to connect women with similar experiences, foster sharing, solutions. Sessions included motherhood transition, postnatal stress management, life planning.<br>Control: no intervention; completing period assessments.	Intervention group (n=30), Control group (n=30).	Intervention group women had significantly decreased depression (BDI) and stress (PSS), significantly increased support (ISEL), compared to control group.	Yes
Sherman et al., 2009 (92)	Thailand	RCT of peer network + life skills intervention by 1 week-trained facilitators.<br>Intervention 1: 7x 2 hour biweekly peer education in groups of 8-12 on sexual risks and methamphetamine. Session 1 built group cohesion. Subsequent sessions enabled participants to share how drug use affected them, their social network and family, discuss sexual risk reduction, STIs, sexual communication, stigma. Interactive games, problem-solving, homework. Optional 2-4 hour community service projects.<br>Intervention 2: 7x 2 hour biweekly skills building based on cognitive behavioural psychology, in groups of 8-12. Sessions focused on causes and consequences of methamphetamine use, the role of stress, STIs, sexual risks. Focus not placed on communicating session content to social network. Content included examining the role played by methamphetamine and its effects, problem solving tools, friendship, STIs, safer sex, stress, coping, managing emotions, self-worth.	18-25 year-old young people. Intervention 1 (n=495), Intervention 2 (n=488)	Significantly improved self-reported methamphetamine use and consistent condom use at 12 months' follow-up in both arms. Incident STIs common but did not differ between arms.	Yes
Noknoy et al. 2010 (93)	Thailand	RCT of Motivational Enhancement Therapy for hazardous alcohol use.<br>Intervention: 3x 15 minute individual nurse-led counselling. Nurses trained for 6 hours to assess severity of alcohol use and effect on health, family, society. Plan and goals agreed during sessions to change drinking and prevent relapse.<br>Control: assessment without counselling.	Intervention group (n=59), Control group (n=58).	Intervention group participants had reduced self-reported drinks per drinking day, frequency of daily or weekly hazardous drinking and binge drinking compared to control group after 3 and 6 months.	Yes
Bolton et al., 2014 (94)	Thailand	RCT of Common Elements Treatment Approach (CETA) for trauma survivors.<br>Intervention: weekly 1 hour flexible treatment tailored to needs, delivered by lay workers for CMDs including depression, PTSD, anxiety, aggression, alcohol, life problem stress. Counsellors and supervisors trained for 10 days; weekly supervision.<br>Control: waiting list.	Burmese survivors of imprisonment, torture, trauma Intervention (n=182), Control (n=165).	Greater reduction in mean baseline depression, anxiety, PTSD, functional impairment, aggression scores in intervention group versus control group participants.	Yes
Assanangkornchai et al., 2015 (95)	Thailand	RCT of ASSIST-verified substance misuse screen + brief intervention.<br>Intervention: 5-13 minute sessions of 10-step ASSIST-linked WHO Brief Intervention for Alcohol Use procedure, to discuss scores, strategies to reduce substance use.<br>Control: 3-6 minute feedback on ASSIST score, meaning, advice to stop/reduce use.	Intervention group (n=120), Control group (n=116)	Significant reductions in substance use but no significant difference between groups. No difference between groups in proportion classified as 'low risk'.	Yes

Shin et al., 2009 (96)	Vietnam	RCT of intervention for parents of children with intellectual disabilities.<br>Intervention: 1 hour weekly teacher-hosted sessions including coaching desired behaviours, with daily homework. Teachers trained for 3 months in early child development, developmental/intellectual disabilities, teaching objectives, task-analysis, feedback on practice with children, families. Supervisors randomly visited children's homes to supervise teachers and provided telephone support.<br>Control: Waiting list.	Preschool-aged children with intellectual disabilities. Intervention group (n=16), Control group (n=14).	Intervention group children improved significantly in most domains of adaptive behaviour (VABS), personal care, motor skills than control group children.	Yes
Bass et al., 2012 (97)	Indonesia	RCT of non-specific mental health counselling program for conflict-affected adults.<br>Intervention: 8x group Problem-Solving Counselling Programme, including qualities of effective helpers, confidentiality, empathy, listening and responding, questioning, problem management skills, stress and coping, consequences and needs of torture survivors. Counsellors received 5 days' training, regular supervision.<br>Control: waiting list.	Intervention group (n=214 across 3 villages), Control group (n=206 across 3 villages).	No difference in depression/anxiety symptom reduction (HSCL-25 or 12 locally identified signs of distress) in intervention versus control group. Mixed impact on functioning (WHODAS 2.0). No increase in positive coping strategy use.	Yes
Armstrong et al., 2010 (98)	Australia	RCT of brief educational intervention training social workers to apply CBT strategies.<br>Intervention: 15 hour in-person course (5 hours over 3 weeks) focused on context and theory around focused psychological strategies, mental health social work in PHC, cognitive behavioural strategies for brief psychological interventions with people with CMDs. Training: presentations, case videos, written material, role plays.<br>Control: Not described.	40 social workers randomised to both arms (proportion not specified).	Intervention group social workers showed significantly more improved objective competence (Cognitive Therapy Scale) and subjective confidence (5-point Likert scale) than control group.	Yes

Supplementary Online Table 2: Non-randomised trials for clinical outcomes

**Authors**	**Country**	**Study design**	**Sample**	**Summary of findings**	**mhGAP-IG recommended intervention or approach?**
Lyketsos et al., 1999 (99)	Argentina	Non-randomised trial of PHC detection and treatment of depression.<br>Intervention: sertraline 50-100 mg daily for 8 weeks in PHC.<br>Control: detection and treatment in psychiatric care.<br>All physicians (PHC and psychiatry) had half-day training on major depressive disorder diagnosis, Hamilton Depression Rating Scale.	Patients with major depressive disorder in primary care: Intervention (n=469), Control group (n=299)	Depression severity (Hamilton Depression Rating Scale) reduced and adherence to treatment high in both groups.	Yes
Edelblute et al., 2019 (100)	Mexico	Uncontrolled study of Promotora support in migration sending area.<br>Intervention: 5x weekly group sessions: 'Women in Solidarity and Support': combination of CBT techniques, psychoeducation, social support activities for depression among women. Lay persons trained for 5 days in social support, mental health education, coping techniques. Facilitators trained to use activities, goal setting, active listening, identifying negative automatic thoughts and role play.	65 women (70% depressed) recruited.<br>39 women attended at least 4 sessions – included in the follow-up data analysis.	Mean CES-D score decreased by 2.4 points. Participants with moderate or high depressive symptoms showed decreased CES-D scores from baseline and non-significant increases in social support. Having a current migrant spouse was the only demographic factor associated with lower odds of improvement in depression. Post- intervention, participants reported increased social support..	Yes
Han et al., 2012 (101)	USA	Uncontrolled study of group therapy for Cambodian refugees.<br>Intervention: 12x weekly trauma-focused psychosocial cultural treatment group (PSCTG) delivered by 2x bilingual Cambodian mental health rehabilitation staff + Buddhist monk paraprofessional.	Intervention group (n=9 out of 57 potentially eligible patients diagnosed with PTSD)	All participants showed significant reductions in total PTSD symptoms post-intervention (HTQ).	No
Ayón et al., 2014 (102)	USA	Uncontrolled study of *promotoras* alcohol-prevention workshops.<br>Intervention: 1 hour educational workshop for adult parents at community centres, housing developments and schools. Workshop included re-enacting lived experience, problem posing + discussion.	85 parents attended the workshop, 71 completed pre- and post-intervention surveys.	Significant improvement in parents' awareness of rates, risks and consequences of alcohol use post-intervention. Information, presentations, promotoras were valued.	Yes
Hovey et al., 2014 (103)	USA	Uncontrolled study of culturally responsive cognitive-behavioural support group for migrant farmworker women of Mexican descent.<br>Intervention: 6x 1.5 hour weekly Spanish cognitive-behavioural groups by clinical psychologist + lay health worker trained in mental and physical health education. Session 1: agenda setting. Sessions 2-6: stress, anxiety, depression, hope, self-esteem, empowering individuation and assertiveness, family issues related to nurturance, childbearing, discipline, family functioning, communication, recovering from domestic violence and other trauma.	6x migrant farm worker women of Mexican descent with elevated levels of depression and stress.	Significant reductions in depression (CES-D), anxiety (PAI anxiety scale), stress (MFWSI), hopelessness (BHS), increased self-esteem (RSEI) scores, immediately post-intervention and at 6 month follow-up. 83% achieved clinically significant reductions in depression immediately post-intervention. All achieved clinically significant reductions in depression at 6 months' follow-up.	Yes

Zámbori et al., 2002 (104)	Hungary	Non-randomised study of mental health treatment delivered in PHC<br>Intervention: 1 year's PHC treatment by 5 qualified psychiatrists, aiming to improve healthcare utilisation and indirect costs of anxiety and affective disorders.<br>Control: treatment as usual, that is by their primary care physicians.	1,815 patients of 12 practices consented. 51/151 agreed to treatment (intervention group). Next 75 eligible patients (control)	The total prescribing cost increased in the intervention group due to psychiatric drug treatment, increasing the overall direct costs of care, partially offset by a 37% decrease in non-psychiatric prescribing. Hospital days decreased in both groups. Absenteeism fell in intervention group. Sick leave days increased in the control group.	Yes
Loughry et al., 2006 (105)	Palestinian territories	Non-randomised trial of child-focused interventions.<br>Intervention: structured activities for children delivered by trained volunteers: cultural, recreational, educational, computer activities as week-long holiday camps. Parents invited to join child activities + local-led parenting activities.<br>Control: No intervention.	250 children from West Bank + 150 from Gaza. Of these, intervention group (n=300), control group (n=100).	Intervention group children had lower Child Behaviour Checklist total problem, externalising problem, internalising problem scores post-intervention. West bank intervention group children reported improved parental support post-intervention (Parental Support Scale).	Yes
Fayyad et al., 2010 (106)	Lebanon	Uncontrolled study of parenting skills for behavioural problems.<br>Intervention: 8x 60-90 minute individual or group sessions for parents from Arabic-adapted Helping Challenging Children manual developed by WPA. Facilitators trained in 4x half days, including recognising, understanding behavioural disorders in 6-12 year-olds, instrument application, intervention delivery. Supervision provided.	20 staff trained across 17 centres.<br>Intervention group (n=87).	Proportion of children scoring 'abnormal' on the SDQ and proportion of women using severe corporal punishment decreased post-training. Majority of mothers reported that the programme helped them to develop new parenting skills.	Yes
Waterman et al., 2018 (107)	Sierra Leone	Non-randomised trial of group therapy for ex-Ebola treatment staff.<br>Intervention: Phase 1: 2 hour workshop including PFA to help staff discuss work challenges + coping. Phase 2: 2 hour workshop on 6 mental health conditions, psychoeducation for specific problems, cognitive behavioural self-help. Phase 3: weekly small-group meetings: low-intensity CBT including behavioural activation, minimising avoidance, problem-solving, coping with anxiety.	Intervention group (n=3,273 ex-Ebola treatment centre (ETC) staff).	Staff mental health (WSM) improved between phases 1 and 3. Clinical measures improved between phases 2 and 3 and pre- and post-phase 3 (WSM, PTSD, stress, sleep, anxiety, depression, anger, relationship difficulties, behavioural problems, alcohol use).	Yes
Sow et al., 2020 (108)	Guinea	Non-randomised study of mental healthcare integrated into PHC.<br>Intervention: workshops and seminars on mental health.to improve staff approaches, attitudes and patient care.<br>Control: no intervention. Patients interviewed post-consultation about perceived involvement (Patient Participation Scale). Providers completed questionnaire on perceived patient involvement.	12x health centres/450 consultations: Intervention (n=175 cons by 7 staff in 4 health centres), Control (n=275 by 11 staff in 8 centres).	Mean Patient Participation Scale higher in intervention than control centres. Participation scores varied by care provider and health centre. All care providers felt they understood patients' concerns; patients agreed. All patients wanted to be involved in decision-making about their treatment; providers were reluctant to do so.	N/A (training)

Souza et al., 2009 (109)	Sudan	Uncontrolled study of intervention for adults with mental disorders. Intervention: Mental health resources provided within PHC: training by psychiatrist for CHWs, physicians, health counsellors on child and adult mental disorders, substance use, epilepsy + basic medications, 1 additional counsellor, MSF treatment guidelines, books, videos.	1st 114 patients (of whom, 81 adults) seen in 1st 6 months' implementation were evaluated.	Commonest diagnoses were epilepsy (47%) and psychosis (31%). Most had never received treatment. Follow-up attendance rates were 58% (1 month) and 48% (3 months). Mean disability score decreased at 1 and 3 months compared to baseline.	N/A (training)
Mutamba et al., 2018 (110)	Uganda	Non-randomised study of lay community health worker group IPT. Intervention: 12x weekly group attachment-focused therapy for depressive symptoms by improving interpersonal functioning and support + usual care. Control: national guidelines for nodding syndrome in children.	Intervention group (n=73 caregivers across 7 villages), Control group (n=69 caregivers across 6 villages).	Depression, distress, stigma, social support improved significantly among intervention group caregivers at 1 and 6 month follow-up compared with control caregivers. Intervention group children had significantly greater depression symptom reductions than control group children at 1 and 6 months.	Yes
O'Donnell et al., 2014 (111)	Tanzania	Uncontrolled study of group trauma-focused CBT by lay counsellors. Intervention: 12x weekly 1 hour separate group sessions for children and guardians with joint activities + 3 individual visits with child and guardian. Sessions included trauma narrative, processing, grief-specific elements. Counsellors had 10 day training + supervision.	Intervention group (n=64 children with at least mild symptoms of grief and/or traumatic stress and their guardians)	Symptoms of grief (Grief Screening) post-traumatic stress (PTSD Reaction Index), depression (Short Mood And Feelings Questionnaire), behavioural adjustment (SDQ) improved post-treatment and sustained at 3 and 12 months.	Yes
Scholte et al., 2011 (112)	Rwanda	Non-randomised study of sociotherapy in post-genocide Rwanda. Intervention: 15x 1 hour weekly groups of 10-15 using interaction to reassess, redefine values, norms, relations, collaborations. Advice, psychoeducation for trauma symptoms. Used debates, experience sharing, coping strategies, games, exercises, mutual support. Local people received 3 months' training + supervision to lead groups. Control: no intervention.	Intervention group (n= 100), Control group (n=100)	Self-reported CMDs (SRQ) reduced significantly in intervention group participants compared to control group.	In part
Murray et al., 2013 (113)	Zambia	Uncontrolled study of trauma focused-CBT for vulnerable children. Intervention: 11x weekly 1-2 hour sessions with children alone, with caregivers, or with caregivers alone. 18 local people trained as counsellors by three local supervisors, attended 2-4 hours' weekly group supervision + 2 hours' weekly individual supervision.	Intervention group (n=58 5-18 year-olds who scored >38 on PTSD-RI)	Significant reductions in severity of trauma symptoms and shame symptoms (SHAME measure) post-treatment.	Yes

Chibanda et al., 2011 (114)	Zimbabwe	Uncontrolled study of locally adapted problem-solving therapy (PST)<br>Intervention: 6x Friendship Bench sessions by trained, supervised, female lay workers. Trainees administered locally developed 7 step plan, including activity scheduling. Session 2 and sometimes other sessions delivered at home. 2 clinical psychologists, a general nurse trained in counselling and a psychiatrist trained 20 staff over 8 days. Sessions included CMDs and identifying them, psychoeducation, PST	Intervention group (n=355 scored above threshold for probable CMD and consented to participate).<br>6 staff participated in a focus group discussion.	Mean CMD score (SSQ) fell after 3-6 sessions, in proportion to the number of sessions attended. Most staff rated themselves as very able to deliver Friendship Bench and rated clients as benefitting. Facilitators included: staff position of trust in community, option of home visits, stigma minimisation, structured intervention enabling progress monitoring and breaking problems down into manageable steps, ability to provide participants with feedback.	Yes
Futterman et al., 2010 (115)	South Africa	Non-randomised trial of Mamekhaya programme for adherence to prevention of mother-to-child transmission of HIV guidelines.<br>Intervention: 8x adapted CBT sessions + mothers2mothers peer mentoring. Sessions covered healthy living, feeling happy and strong, partnering and preventing transmission, parenting.<br>Control: standard services provided by midwives and counsellors.	Intervention group (n=83 HIV-positive pregnant women),<br>Control group (n=77).	Intervention group women showed significantly greater improvement in establishing social support (Medical Outcomes Study social support survey) and depression (CES-D) than control group women. Intervention participants had better attendance at follow-up medical visits, greater improvements in positive coping (brief COPE scale) and increased HIV knowledge.	Yes
Van't Hof et al., 2011 (116)	South Africa	Uncontrolled feasibility study of brief problem-solving therapy.<br>Individual condition: participants completed self-help booklet alone for 5 weeks, weekly brief phone support by coaches.<br>Group condition: weekly 45-60 minute meetings over 5 weeks, discussing materials + phone support from trained coaches.	Volunteers experiencing psychological distress from 4 different communities (n=103).	73 participants completed 5 week intervention. Psychological distress (K-10, SRQ) fell significantly, programme positively rated: most found sessions helpful, booklet easy to read, language comprehensible, homework useful, would recommend the course. Significantly more dropouts from individual than group condition.	Yes
Nimgaonkar et al., 2015 (117)	India	Uncontrolled study of mental health task-shifting.<br>Intervention: CHWs trained in supportive counselling, education, pharmacotherapy, identification and referral to community hospital. Training based on manualised curriculum: lectures, review sessions, patient video clips, brief skits, role plays. Evaluation 3 years later.	Intervention group (n= 184 + 508 staff filled 2 surveys). Medical records of 268 patients in 118 villages reviewed.	Treated patients showed significant improvements in daily functioning. Mean treatment adherence scores remained stable during treatment. Proportion of self-referrals increased by 30% over three years. Surveys found improved knowledge, attitudes, acceptance of mental illness by community.	Yes

Kaufman et al., 2013 (118)	China	Uncontrolled study of child group therapy by 8 lay counsellors.<br>Intervention: 4-6x 90 minute community-based group counselling sessions (2 with caregivers). Focused on self-awareness, communication. Based on curriculum for family survivors of suicide + group IPT model used in Uganda. Staff trained by psychiatric nurses. Several school principals also participated for their learning.	Intervention group (n=39 children screened as meeting diagnostic criteria for anxiety and depression)	Significant improvement in anxiety but not depression scores immediately post-intervention, less marked at second follow-up.	Yes
Parantha-man et al., 2010 (119)	Malaysia	Non-randomised psychoeducation for caregivers by health workers.<br>Intervention: 5x 1 hour structured psychoeducation over 2 weeks. Covered schizophrenia, treatment, relapse prevention, crises, healthy lifestyle. Lectures with audio-visual aids, charts, booklets. Nurses/medical assistants trained by psychiatrist, psycho-educators.<br>Control: no intervention.	Intervention group (n=54),<br>Control group (n=55).	Intervention group caregivers showed significant improvements in knowledge and perceived burden (Family Burden Interview Schedule) relative to the control group, post-intervention. Intervention group patients had improved follow-up relative to the control group, post-intervention.	Yes
Puspitosari et al., 2019 (120)	Indonesia	Non-randomised study of community-based rehabilitation for people with schizophrenia.<br>Intervention: 12 weeks' psychoeducation + social skills training for patients. Psychoeducation only for caregivers.<br>Control: routine outpatient care through PHC centre.	Intervention group (n=50), Control group (n=50)	Higher proportion of intervention group participants had increased quality of life post-intervention than control group. Quality of life improvements in the intervention group were significantly higher than those of the control group.	Yes

Supplementary Online Table 3: Non-randomised trials for training outcomes

Authors	Country	Study design	Sample	Summary of findings
Goncalves et al., 2013 (121)	Brazil	Uncontrolled study of training to enhance mental health and PHC collaboration to improve care.<br>Intervention: 6x seminars + 8x practical clinical rounds in PHC setting. Training lasted 96 hours over 10-12 weeks.	240 professionals trained.	Recognition increased significantly among nurses but decreased significantly among GPs at 6-8 month follow-up. Significantly increased patient-centred care but no change in psychosocial interventions, shared care, secondary care referrals.
Anderson et al., 2017 (122)	Colombia, Mexico, Peru	Protocol: Non-randomised study of PHC-based prevention and management strategies to address heavy drinking in 3x cities in Colombia, Mexico and Peru, compared with 3x comparator cities.<br>Intervention: training and supportive municipal action in 18 months.<br>Control: Comparator cities' PHC units will proceed with usual care.<br>Primary outcome: AUDIT-C positive patients receiving verbal advice/ referral for advice in or outside PHC units. Provider attitudes and alcohol literacy measured by SAAPPQ.	10x PHC units per city (60 in total). Intervention delivered to patients identified in PHC as being at risk of heaving drinking.	Aims:<br>1) Train staff in prevention, early identification of heavy drinking.<br>2) Set up and implement training with stakeholders in the 3 cities.<br>3) Test impact of package scale up on provider delivery, early identification, management by comparing changes in screening, advice, referral among intervention and control city PHC units.<br>4) Identify facilitators and barriers to full-scale up.<br>5) Present validated framework and strategy for full scale.
Cook et al., 2017 (123)	USA	Uncontrolled study of motivational interviewing (MI) training.<br>Intervention: one-off or divided workshops (1-3 months apart) lasting 4 or 8 hours, with didactic content, clinical examples, role plays. Some participants requested booster 1-6 months later.	394 trainees	In 28 workshops over 10 years, participant diversity and % with prior MI training increased, average age and years in practice decreased. Outcomes varied by discipline: mental health professionals, case managers, health educators, nurses had higher scores on most outcome variables than non-health professionals.
MacCarthy et al, 2013 (124)	Canada	Uncontrolled study of mental health module for PHC physicians.<br>Intervention: 3 days' training over 5 months. Day 1: overview + cognitive behavioural interpersonal skills manual, assessment tools, handouts, accessing Bounce Back telephone coaching. Day 2: shared learning opportunities, challenges, solutions using video and role-play, then Antidepressant Skills Workbook. Day 3: cognitive behavioural interpersonal skills self-assessment questionnaire, medication, community resources, fee code training (for billing).	>1400 family physicians in British Columbia (out of a total 3300) started training.	Participants reported success in implementing self-management tools in their practices, with positive impacts on patients. Improvements were maintained or improved at 3 to 6 months post-training. Stigmatising attitudes towards mental illness decreased post-training (using An Opening Minds Survey for Health Care Providers).

Ravitz et al., 2013 (125)	Canada	Non-randomised study of training for non-medical community staff.<br><u>Intervention:</u> weekly interactive course (total: 5 hours) on fundamentals of CBT, IPT, MI, dialectical behavioural therapy. Course 1 focused on CBT for depression, Course 2 focused on MI for concurrent disorders. 4 community settings assigned Course 1 small group learning (n=35), 3 to Course 1 self-directed learning (n=28).	93 non-medical front-line workers.<br>Course 1 (n=63),<br>Course 2 (n=30)<br>Focus groups with 25 participants.	Knowledge improved significantly, post-course. Counselling self-efficacy improved in Course 1 participants. Dropouts less frequent in peer-led, small-group learning than self-directed format. Focus groups found improved confidence, morale, self-reported practice change, increased comfort working with challenging clients.
Ruud et al., 2016 (126)	Norway	Uncontrolled study of mental health staff training.<br><u>Intervention:</u> 2 year programme for 1 day per month (total: 80 hours) + fortnightly multidisciplinary small-group supervision (total: 80 hours), fortnightly small-group theoretical discussion (total: 40 hours) writing clinical cases (group task). Lectures included service users and caregivers. Content comprised understanding psychosis and treatment, building/maintaining relationships with service users, awareness of interactions, understanding lived experience, multidisciplinary collaboration, supporting caregivers, self-care.	> 100 local training programmes, enrolling > 3500 participants.	Clinical competence, understanding and respect for professional groups and service levels, focus on service user involvement and influence on treatment improved post-intervention. Recruitment, participant, student and service manager ratings were positive.
Bowers et al., 2009 (127)	UK	Uncontrolled study of training for non-medical mental health staff.<br><u>Intervention:</u> 14x educational seminars to prepare for 'New Ways of Working' role changes. Included interactive sessions on clinical skills, confidence, assessment and management of mental illness.	26 members of a community mental health team.	Confidence in assessing mental health problems and diagnosing mental disorders improved. Participants increased their confidence and familiarity with NICE guidelines.
Ferraz et al., 2009 (128)	UK	Uncontrolled study of solution-focused brief therapy training.<br><u>Intervention:</u> 6x 2 day training courses, each regrouping up to 20 trainees with varied professional backgrounds + fortnightly group supervision for 6 months. Aim: to develop confidence, skills, knowledge of solution-focused brief therapy.	66 participants	Trainees' focus on patient strengths and goals increased at 6 month follow-up. Trainees reported using specific techniques (e.g. the 'miracle' or 'preferred future' questions, 'exception' questions, 'coping' questions) significantly more often at follow-up. Trainee use of praise and reward showed a non-significant increase.
Chew-Graham et al., 2014 (129)	UK	Qualitative study evaluating GP practice training in underserved communities, reviewing mental health codes in records.<br><u>Intervention:</u> Up to 6 sessions of Access to Mental Health in Primary care (AMP) course with 3 core components: community engagement, PHC quality, psychosocial interventions. AMP training *plus* offered flexible package tailored to PHC teams' local needs. Included reviewing health system organisation and structure, active linking to local organisations and resources.	13 semi-structured interviews + 2 focus group discussions (n=14) across 6 practices.	Participants reported increased awareness, recognition, respect for needs of patients from under-served communities. Subsequent changes in style and content of interactions; evidence of system change. Community agencies in the practice locality became more aware of mental health.

Sadik et al., 2011 (130)	Iraq	Uncontrolled trial of interactive training for health workers. <u>Intervention:</u> 8x 2 week courses offered for PHC staff adapted for Iraq from Kenyan model. Courses covered mental health using theory, role play, small/large group discussion, video, clinic visits.	Intervention group (n=387 health workers) from 13 health centres.	Test scores increased post-training. Trained staff deemed to be more highly skilled than untrained staff, according to observing research psychiatrists. Exit interviews highlighted improved skills.
De Jong et al., 1996 (131)	Guinea-Bissau	Uncontrolled study of PHC training on mental disorders, epilepsy. <u>Intervention:</u> 12x 5 day courses on 10 mental health problems. Used theory, role play, exercises, sharing stories. Quarterly supervision.	600 staff trained over 3 years, treated 351 adults 100 children.	Staff diagnosis of major mental disorders and epilepsy and knowledge of treatment increased post-training.
Adeyemi & Jegede, 1994 (132)	Nigeria	Uncontrolled study of mental health teaching and feedback for GPs. <u>Intervention:</u> Psychiatrists informed GPs of Present State Examination (PSE) symptoms, taught GPs how to use the PSE interview schedule questions, daily feedback for 3 months.	7 general practitioners treating 399 patients.	3 GPs' capacity to detect mental health problems increased, 2 showed no improvement, 2 showed some deterioration. Years of experience influenced motivation but not improvement.
Odejide et al., 2002 (133)	Nigeria	Uncontrolled study of non-medical PHC staff training on depression. <u>Intervention:</u> . 2 day training: recognising and managing depression in PHC using WPA guidelines. Training developed based on focus group and survey knowledge and needs assessment of 62 staff.	Unclear how many staff trained.	At baseline, staff had no mental health training and could not name an antidepressant. Knowledge and skills improved significantly, post-training, especially of drug management.
Omigbodun et al., 2007 (134)	Nigeria	Uncontrolled study of child mental health training. <u>Intervention:</u> 3 day course on child mental health problems, impact, normal child development, basic child mental health assessment, basic treatment and management, multidisciplinary teamwork. Sessions used theory, pair/group discussion, case demonstrations.	Intervention group (n=38) staff.	Mean mental health knowledge score increased post-training. Most participants were satisfied with the course and thought it would impact their practice, especially collaboration.
Abayomi et al., 2013 (135)	Nigeria	Uncontrolled study of mental health education for volunteers. <u>Intervention:</u> 6x 1 hour sessions over 6 weeks by 3 psychiatrists + 1 psychologist. Content included manualised mental health literacy for village health workers, aetiology, clinical features, care, treatment.	Intervention group (n=31 community volunteers).	Perceived dangerousness and attitudes toward people with mental illness significantly improved post-training on the Familiarity with Mental Illness Questionnaire and a modified Bogardus Social Distance Scale.
Makanjuola et al., 2012 (136)	Nigeria	Uncontrolled study of mental health training for CHW tutors. <u>Intervention:</u> 5 day workshop in 5 units, followed by supervision. 1) core mental health concepts, 2) core skills (communication, assessment, mental state examination, diagnosis, management), 3) common neurological disorders, 4) WHO mental health PHC guidelines, 5) policy, legislation, links between mental and physical health, collaborative working integrating mental health into plans.	Intervention group (n=24 community health office trainers).	Overall mental health knowledge increased after training. Trainees showed fewer negative attitudes (e.g. perceived dangerousness) towards people with mental illness reduced post-training.

Tilahun et al., 2019 (137)	Ethiopia	Non-randomised study of training for rural health extension workers<br>Intervention 1: 10x session basic training on mental health assessment, management, prevention. 1 session on child development, child mental health, developmental disorders.<br>Intervention 2: enhanced training: basic + video training on developmental disorders. 5x short videos modelled interviewing mothers of children with autism/intellectual disability: early detection, supportive counselling, problem solving. Pocket guide: detection, mental health first aid, supporting families.<br>Control: no mental health training.	Intervention 1 (n=104 health extension workers),<br>Intervention 2 (n=97),<br>Control group (n=108).	Fewer negative beliefs and reduced social distance towards children with autism in both intervention groups compared to control group post-intervention. Improvements were greater for intervention 2. However, intervention 2 health extension workers less likely to have positive expectations of children with autism than control group.
Kabura et al., 2005 (138)	Uganda	Uncontrolled study of training for informal helpers.<br>Intervention: 5 module micro counselling skills training for informal helpers to facilitate helping relationships. Modules covered distinct behavioural skills of therapeutic communication. Training comprised theory, practice exercises, role plays, video demonstrations.	Intervention group (n=44 informal professional helpers).	Trainees assessed as proficient in basic counselling skills (Attending Behaviour Rating Scale Plus) and gained knowledge (Basic Skills Knowledge Test) of these skills, post-training.
Abbo et al., 2013 (139)	Uganda	Uncontrolled study of Global Assessment of Functioning (GAF) tool.<br>Intervention: Video of psychiatrist using GAF to assess patient with schizophrenia shown to psychiatric clinical officers who made their own assessment. 1 hour video on GAF concepts and categories, with case vignette examples, then repeated the first exercise.	Intervention group: (n=5 psychiatrists + 5 psychiatric clinical officers).	Intra-class correlation coefficient between psychiatrists and psychiatric clinical officers pre-training improved post-training.
Jenkins et al., 2010 (140)	Kenya	Mixed methods study evaluating PHC staff training.<br>Intervention: 5 day course included in national training delivery system with supervision. Included impact of mental health on physical health, economic/social outcomes, communication skills, assessment, diagnosis, management, neurological disorders, mental disorders, policy, legislation, recording systems, collaboration.	41 trainers trained 1671 PHC staff.<br>200 supervisors were also trained.	Mean PHC staff knowledge increased post-training. Clinical observations noted improved assessment, diagnosis, management, record keeping, medicine supply, intersectoral liaison, communication skills, public education, therapeutic relationships, know-ledge of links between mental and physical health.
Cettomai et al., 2011 (141)	Kenya	Uncontrolled study of non-physician health worker training.<br>Intervention: 1 day module for non-physician health workers on neurological examination, localisation, diagnosis, treatment of HIV neurological complications, HIV dementia, peripheral neuropathy. Sessions used theory, small group activities, case studies.	Intervention group (n=71 health workers).	Of the 55 staff completing pre- and post-intervention surveys, participants identified more aetiologies of neuropathy post-training. Challenges persisted in identifying HIV dementia diagnostic criteria.
Kauye et al., 2011 (142)	Malawi	Uncontrolled study of health surveillance assistant (HSA) training.<br>Intervention: 3 days' training in 1) mental health and illness, 2) Mental Health First Aid, 3) promoting mental health, combatting stigma. 2 days covered 1) and 2), 3) after 6 months. Sessions used theory, discussion, role play. Trainees supervised monthly.	Intervention group (n=271 staff).	Programme well-received by trainees. Most thought course was clinically useful, content at the right level of difficulty.

Wright et al., 2014 (143)	Malawi	12 month follow-up of uncontrolled study of HSA training. <u>Intervention:</u> As above. Training delivered by psychiatric clinical officers, psychiatric nurses, a psychologist and a service user.	Intervention group (n=271 staff).	HSAs' knowledge and confidence in providing care improved significantly. HSAs initiated dialogue on mental health promotion and human rights (patient records review).
Byrne et al., 2004 (144)	South Africa	Uncontrolled study of child and adolescent mental health training. <u>Intervention:</u> 2 days' training (~3.5 months apart) for primary care nurses on basic assessment and intervention skills. Day 1: role of health service, assessment process with clinical examples, adapting model, observed diagnostic interview, discussion. Day 2: seminars on group-identified priorities, e.g. enuresis, diagnostic interview.	Intervention group (n=144 nurses attended Day 1, of whom 68 attended Day 2).	Training rated positively by nurses, who recommended additional training in future. Nurses reported applying counselling skills in practice after Day 1. Unmet training, consultation and supervision needs identified.
Bradshaw et al., 2006 (145)	South Africa	Uncontrolled study of training for volunteer health workers. <u>Intervention:</u> 3 day course: understanding mental illness, drug treatment, responding to people with mental health problems, helping families cope, community and social interventions.	Intervention group (n=21 volunteers) participated in post-course focus group.	Improved understanding of aetiological contributors to mental health problems, identification of symptoms and strategies, awareness of impact of interpersonal behaviours on people with mental health problems.
Armstrong et al., 2011 (146)	India	Uncontrolled study: CHW mental health training in rural Bangalore. <u>Intervention:</u> 4 day manualised course developed for CHWs in India. Training included introduction to mental disorders, Mental Health First Aid, practice-based skills, mental health promotion.	Intervention group (n=70 CHWs).	Training associated with improved recognition of mental disorders from a vignette, reduced endorsement of unhelpful/potentially harmful beliefs about medication, small reduction in stigmatising attitudes. Unclear impact on belief in recovery.
Paudel et al., 2014 (147)	India	Qualitative study of village health worker mental health training. <u>Intervention:</u> comprehensive rural health project provided training on mental health, anxiety, depression, risk factors, symptoms, management. Used theory, skits, songs, discussions.	Trained village health workers in 5 focus groups (n=24 out of 36 eligible staff) of 4-6.	Most groups correctly diagnosed depression from a vignette, post-training. Staff could identify symptoms and suggest depression management options.
Hofmann-Broussard, 2017 (148)	India	Non-randomised study of mental health training for CHWs. <u>Intervention:</u> 4 day 12 module manualised training. <u>Control:</u> no intervention.	56 community health workers (intervention = 34; control = 22)	Statistically significant increase in mental disorder recognition from vignettes by intervention group staff, decreased stigmatizing opinions, increased perceived mental health competence.
Malla et al., 2019 (149)	India	Uncontrolled study of lay health worker treatment in conflict setting <u>Intervention:</u> 40 rural lay health workers trained to provide basic mental healthcare, supported by low-cost technology + specialist (psychiatrist, social worker, psychologist) consultation, supervision.	Intervention group (n=262 14-30 year-olds meeting criteria for major mental disorder.	Significantly more patients identified during 14 month project than among all age groups in preceding 2 years. At 12 months, 78% of patients still engaged with the service. Significantly improved functioning (GAF), quality of life (WHOQOL-BREF), disability.

Jordans et al., 2012 (150)	Nepal	Uncontrolled study of brief training for front-line staff. Intervention: 2 day (14 hour) training following IASC guidelines on mental health and psychosocial support in emergencies. Included understanding mental health and psychosocial concepts, impact of emergencies, humanitarian operations on mental health, guideline applications, support options, emergency responses, facilitating collaboration among partners. Used theory, discussion, exercises.	Intervention group (n=109 trainees).	Knowledge scores increased by 21% post-training and by a further 25% at 2 month follow-up.
Li et al., 2014 (151)	China	Uncontrolled study of community mental health staff training. Intervention: 1 day course: Part 1: introduction to mental disorder symptoms, diagnosis, treatment. Part 2: mental health stigma.	Intervention group (n=99 staff).	Mental health knowledge improved significantly. Overall stigma (MICA) decreased post-training and RIBS score increased.
Lam et al., 2016 (152)	China	Uncontrolled study of training for traditional Chinese medicine staff. Intervention: 10x interactive seminars on common psychological problems and psychotherapy, to increase recognition, diagnosis, management, referral for mental health problems in PHC.	Intervention group (n=158 Traditional Chinese Medicine practitioners).	Significantly increased confidence in recognising symptoms, diagnosing CMDs and managing them. Qualitative results: perceived increased awareness of symptoms, diagnosis and management. Barriers: difficulty understanding English terms, time constraints, lack of formal psychiatric referral.
Yang et al., 2018 (153)	China	Uncontrolled study of interdisciplinary community training. Intervention: 3 day training based on Chinese Basic Public Health Services requirements, WHO Mental Health Action Plan. 13x sessions on community mental health concepts (recovery model, mental health law, case management, referral, risk assessment, emergencies). Included problem-solving exercises, handouts, audio-visual presentations, discussions, role plays, simulated scenarios.	Intervention group (n=48 community mental health professionals from colleges, hospitals and community health centres.	Improved knowledge post-intervention. Most participants very satisfied.
Graham et al., 2010 (154)	Australia	Uncontrolled study: Mental Health Aptitudes into Practice training. Intervention: core modules: introduction to mental health and mental illness, depression and treatment, anxiety and treatment.	Intervention group (n=1126)	Participants increased confidence in ability to work with people with mental health problems and less desire for social distance from them. Participants' mental health knowledge and skills increased. Changes immediately post-training and appeared to be maintained 6 and 12 months later (limited evidence).
Hossain et al., 2010 (155)	Australia	Uncontrolled study of Mental Health First Aid training to address mental ill-health in rural areas, including among farmers. Intervention:12 hours' training over 2 days. Content included overview of major mental health problems in Australia, 5 steps of Mental Health First Aid, applied steps to address problems related to depression, anxiety disorders, psychosis, substance use disorders.	Intervention group (n=32 advisory extension agents). 21 interviews + 15 surveys. 6 supervisors interviewed. 2 staff/ farmer focus groups	Participants' confidence and knowledge about mental health, empathy towards people with mental health problems increased 1 year post-intervention. Perceived usefulness of mental health training for advisory and extension agents confirmed among farmers and agents.

Morawska et al., 2013 (156)	Australia	Uncontrolled study: Mental Health First Aid training for PHC doctors. Intervention: 2-day manualised workshop. Content included ways of assisting people in mental health crisis or with early mental health problems. Participants learned about symptoms, risk factors and how to help. Training offered to 27 groups by 7 trainers.	Intervention (n=458).	Participant recognition of mental health problems, concordance with PHC physicians about treatment (assessed using 2 vignettes), confidence in providing first aid, help provided to others increased post-participation. Stigmatising attitudes (Personal and Perceived Stigma Scale) reduced post-participation. Results maintained at 6 months.

Supplementary Online Table 4: Non-randomised studies for implementation outcomes

Authors	Country	Study design	Sample	Summary of findings
Agrest et al., 2019 (157)	Chile	Qualitative study of task shared psychosocial intervention. Intervention: Critical Time Intervention-Task Shifting (CTI-TS) project combined task-shifting strategies to deliver psychosocial interventions by CHWs and peer support workers in the person's home or other community setting.	Interviews with intervention service users (n=15)	1) intervention structural characteristics beneficial, especially timing, duration, phasic nature, 2) community-based approach acceptable: allowed staff to observe and understand contexts, 3) peer support workers and CMHWs appreciated by participants.
Zeligman et al., 2019 (158)	Mexico	Qualitative study exploring Mental Health Facilitator programme trainees and service recipients. Intervention: 30 hours' training on recognising stress/distress, making referrals, drawing on community services, culture and value-relevant helping responses. Same programme also trained mental health facilitator trainers and master trainers.	12 mental health facilitators, 9 people who received services from mental health facilitators.	Barriers and facilitators to integrating the programme into the community discussed alongside generalisability, impact on family dynamics, roles, relationships, programme strengths, including how personal characteristics influenced their perceptions and implementation, and improvements.
Church et al., 2010 (159)	Canada	Mixed methods study evaluating interprofessional education programme for mental health professionals. Intervention: 10x video-conference sessions of Rural Mental Health Interprofessional Training Programme. Content included MI, interprofessional team development, relationship building, stages of change model, solution-focused therapy, CBT approaches, assertive community treatment, crisis intervention. Included small group practice, discussions, interactive exercises.	125 professionals from 15 professions were trained. 8 focus groups with 3-6 participants each were conducted. 12 participants were interviewed. Focus groups and interviews included representatives of 10 of the 15 professions.	High programme satisfaction, especially interaction with other professionals, but issues with videoconferencing. Professionals' confidence significantly increased for most topics. Facilitators: scheduling over extended period, positive relationship with facilitator, experiential learning format, community coordinators. Challenges: professionals' varied mental health experience, work demands.
Fendt-Newlin, 2018 (160)	Sierra Leone	Mixed methods study exploring feasibility and acceptability of co-producing a culturally relevant social intervention for mental healthcare delivery by practice of nurses who received mhGAP training in 2013, over 9 months. Intervention: 5 day *Sababu* training: psychological first aid, bio-psychosocial model, psychosocial skills, training of trainers.	20 nurses trained across 14 districts. 31 individual interviews with nurses and supervisors at 3 and 9 months + 16 focus group discussions with 36 participants in the UK and Sierra Leone, including 2 with newly trained mental health nurses.	Several iterations of intervention due to Ebola outbreak. Pilot showed post-training improvements in communication skills, building relationships with service users, identifying assets, linking to community resources. Barriers to embedding into nurse practice were stigma, district manager and policy maker reluctance to change, financial and time constraints.

Waterman et al., 2019 (161)	Sierra Leone	Feasibility study of stepped-care peer-delivered group CBT. Intervention; 6 week group CBT for depression/anxiety. 12 ex-Ebola treatment centre (ETC) staff trained to facilitate intervention; weekly support and coaching from UK specialists.	Intervention group (n=253). 157 participants participated in a 45-60-minute interview.	Barriers: lack of motivation to attend, low literacy, differing cultural conceptualisations of mental health problems, resource constraints. Facilitators: novelty of CBT for some, encouragement by social networks.
Adams et al., 2020 (162)	Sierra Leone	Qualitative study evaluating perceptions of Community Mental Health Forums (CMHFs), aiming to promote mental wellness and prevent human rights abuse and negative outcomes. CMHFs included formal and informal care providers and were designed to facilitate open dialogue through participatory workshops.	10 mental health nurses and 52 CMHF participants participated in interviews	CMHF implementation affected by: traditional beliefs and culture, health system, inclusive approaches. Participants thought CMHFs improved awareness, beliefs and behaviours towards people in psychological distress and increased cooperation between practitioners.
Makan-juola et al., 2012 (163)	Nigeria	Qualitative study of training of 24 PHC tutors and their students. Intervention: 5 day workshop for college teachers, who taught mental health to community health officers. Training aimed to reinforce the relationship between mental and physical health, and social issues in PHC, via theory, role play, discussions, videos.	In-depth interviews (n=3 tutors). 2 focus group discussions (n=8 students/ group). Lecture observations (n=4 tutors selected at random).	Tutors reported positive impact of training on their mental health teaching. Direct observation showed that 3 exhibited high fidelity to course material. Students reported improved quality of mental health classes.
Mutamba et al., 2018 (164)	Uganda	Mixed methods study adapting group IPT by lay CHWs for caregivers and children affected by nodding syndrome. Evaluation: document reviews, district consultation meetings, stakeholder workshops, qualitative assessment using Replicating Effective Programmes framework to guide contextualisation.	Document reviews: 34 publications 63 caregivers attended groups and completed post-assessment measures.	>90% of beneficiaries completed treatment. Training, logistics, technical assistance strategy adaptations included content modification based on qualitative findings. Training shortened for feasibility. Peer-to-peer supervision considered sustainable quality assurance method.
Papas et al., 2010 (165)	Kenya	Mixed methods adaptation and feasibility study of group CBT by paraprofessionals to reduce alcohol among HIV-infected people. Intervention: 6 session manualised CBT in 5 groups (total n=27) with paraprofessional manual + training workbook, developed with panel + research team input. Training comprised role play, videotaped feedback, ethics, basic health education. Counsellors supervised using Yale Adherence and Competence Scale.	Clinical (8 hours' alcohol support group observations), patient interview (n=14), focus group discussion + lay panel discussion qualitative data used to adapt intervention to be culturally relevant, assess compatibility with conceptual model of alcohol use.	Adaptations to concepts, language, therapists, metaphors, content, length. Socioeconomic, legal, psychosocial contextual issues considered. 77% attendance. Days abstinent from alcohol pre- intervention were 52-100% (women)/21-36% (men). Post-intervention days abstinent from alcohol: 96-100% (women)/89-100% (men). Participants satisfied.
Jenkins et al., 2013 (166)	Kenya	Qualitative evaluation following cluster RCT of PHC mental health training, to understand results and staff experiences. Intervention: WHO PHC guidelines accompanied by supervision and availability of medicines within participating clinics.	Interviews (n=14 intervention group PHC staff + 11 control group PHC staff). 2x focus group discussions.	Intervention group PHC staff identified improved communication, diagnostic and counselling skills, which they thought patients noticed and appreciated. Control group PHC staff noted a lack of these skills.

Othieno et al., 2013 (167)	Kenya	Qualitative evaluation following cluster RCT of PHC mental health training supplemented with supervision.<br>Intervention: 5 day, 40 hour course using theory, discussion, role play, video. 5 modules covered skills, neurological and mental disorders, health system issues, policy, legislation, links with wider health, roles, responsibilities, record systems, working with CHWs and traditional healers, disaster planning, integrating mental health into operational plans.	Interviews (n=10 intervention group patients + 10 control group patients).<br>2x focus group discussions.	Intervention group patients noticed and appreciated enhanced communication, diagnostic and counselling skills employed by their health workers. Control group patients identified their health workers' lack of these skills.
Petersen et al., 2011 (168)	Uganda, South Africa	Qualitative study exploring how a common implementation framework could aid integration of mental health into PHC, to address service gaps for CMDs (South Africa) and SMI (Uganda).	South Africa: 15 CHWs trained to implement framework participated in 4 focus group discussions.<br>Uganda: service users, carers, forum members, CHWs who received mental health training participated in focus groups. Health managers, state health service psychiatric nurse, 2 general nurses, 2 medical officers, 2 carers interviewed.	Political support for mental health strengthening improved by sensitising district managers, establishing collaborative multi-sectoral community forums. Task-shifting CHWs to improve help-seeking and service access. In Uganda, a referral bottleneck arose from limited application of task-shifting and limited availability of medication and specialists. Community self-help groups showed potential to empower service users and carers, promote social inclusion, address stigma, discrimination, human rights in both countries.
Petersen et al., 2012 (169)	South Africa	Non-randomised feasibility study and process evaluation of manualised group IPT by supervised CHWs.<br>Intervention: 12 week manualised group IPT by supervised CHWs trained over 2x 4 day courses by 1 psychologist + 1 trainee.<br>Control: EUC. All staff trained in detection, counselling.	Moderate-severe depression (BDI): Intervention (n=30), control (n=30). Interview (n=9 patients + 2 CHWs).	Good retention of intervention group participants, significant reduction in depressive symptoms post-intervention and 24 weeks post-baseline, compared to the control group. Process evaluation: participants thought that social support, coping skills, improved personal agency could have reduced depression further.
Sorsdahl et al., 2015 (170)	South Africa	Uncontrolled feasibility study of blended (supervised) peer MI + PST.<br>Intervention: Session 1: ASSIST-linked brief MI by peer counsellor. Other sessions: problem-solving skills, booklet, homework.<br>Training: MI (18 hours) + MI booster (3 days) + PST (12 hours) + drugs, alcohol, risk, ethics, protocol, confidentiality, referral.<br>Pre/post-questionnaires: substance use, problem-solving styles, depression. Interviews on barriers and facilitators.	People screened as 'at risk' on ASSIST (n=20).	15 participants completed intervention + 3 month follow-up. Substance use (ASSIST) significantly reduced at follow-up. Participants reported that intervention helped them identify negative effects of substance use. Most participants requested more sessions. Participants highlighted booklet user-friendliness and usefulness.

Myers et al., 2019 (171)	South Africa	Uncontrolled feasibility study comparing dedicated (new CHWs with sole responsibility for counselling) and designated (additional responsibility for existing CHWs) delivery models.<br>Intervention: structured mental health counselling.	Patients diagnosed with chronic diseases (HIV: n=20 and diabetes: n=20) reporting hazardous use of alcohol or depression.	67% of eligible patients willing to receive counselling (higher interest for depression than alcohol use). 85% retention in counselling and 90% in the study. CHWs considered dedicated and designated approaches acceptable but requested more training and support.
Spedding et al., 2020 (172)	South Africa	Uncontrolled study of task-shared PST for antenatal CMD symptoms.<br>Intervention: 3x PST sessions by a dedicated counsellor. Session 1: identifying priorities, skills for problem-solving what is important. Session 2: adaptive coping strategies for problems that cannot be solved. Session 3: review of problem-solving and coping skills.	Intervention group (n=38 pregnant women scoring >14 on EPDS).	Significant reduction in EPDS and psychological distress (SRQ-20). Acceptable due to opportunity for confidential disclosure of problems, relieved staff from managing distress (lightening their workloads). Barriers included lack of transport, women's other commitments and demands of an already burdened system.
Abas et al., 2016 (173)	Zimbabwe	Mixed methods study investigating the acceptability and implementation of brief PST, 4-8 years after the pilot study.<br>Intervention: Friendship Bench comprised PST delivered by older female lay health workers in Harare. Participants were identified through PHC, community agency or self-referrals. Therapists performed structured psychosocial assessments, gave advice, delivered PST or discharged participants. Volunteer counsellors provided supervision during the second implementation.	5 focus group discussions with lay health workers (n=8-12 per group).<br>12 in-depth interviews (n=6 patients and n=6 staff).<br>Routine attendance data reviewed from 2010-2014.	Intervention acceptable: consistent attendance, staff recruitment, perceived benefits. Lay health workers felt satisfied. Facilitators: therapist characteristics (status, maturity, trust, continuity), intervention (validated screen, perceived relevance). Challenges: weekly supervision of therapists and supervisors required despite years of experience, need for training to support certain needs, poor documentation; lack of depression follow-up, low antidepressant access.
Nyama-yaro et al., 2020 (174)	Zimbabwe	Case series evaluating culturally adapted, integrated PST (*Nzira Itsva*) for antiretroviral therapy adherence and depression.<br>Intervention: 6x adherence counsellor-led sessions. #1: PST for adherence + 5 sessions: PST for depression + adherence review.	Detailed case histories (n=3 out of 9 participants).	Intervention acceptable: attended all 6 sessions. Mean CMD symptom scores based (SRQ-8).

Pereira et al., 2011 (175)	India	Qualitative study exploring experiences of integrating a stepped care lay health counsellor intervention into PHC for a cluster RCT. Intervention: trained lay health counsellor collaborated with GP, visiting mental health specialist in public and private healthcare. Control: no intervention.	Post-trial interviews with key members of intervention arm PHC teams (n=119) Interviews with control clinic staff (n=48).	Facilitators: screened CMDs, assessed severity, visiting psychiatrist, managed adherence, provided support. Challenges: 1) non-adherence (symptoms improved/ doubted interventions could affect life difficulties. 2) Planned in-person IPT rarely delivered (travel costs, work commitments). 3) Struggled to support extreme social difficulties, alcoholism, elderly. Control arm GPs adopted intervention-like practices: routine CMD diagnosis, psychoeducation, antidepressants, referral.
Muke et al., 2019 (176)	India	Qualitative study of digital training platform for lay counsellors. Intervention: Prototype digital modules based on Healthy Activity Program training introduced depression, screening, counselling. Results informed modifications.	CHWs participated in 3 focus group discussions (n=32).	1) Training considered important for 'stress'/'tension', 2) digital platform useful, convenient despite limited digital experience, 3) simpler language, interactive content, images could increase interest, engagement.
Fisher et al., 2014 (177)	Vietnam	Follow-up study evaluating translated, culturally adapted version of Thinking Healthy Program (CBT) for pregnant women who were not required to score in the clinical range for depression, anxiety and stress symptoms (DASS-21-V) to participate.	Pregnant women in rural Vietnam who completed baseline and process surveys (n=6). Pregnant women (n=6), commune and women's union representatives (n=2) who answered the exit survey.	Adapted intervention understandable, meaningful, relevant to women, commune health centre and Women's Union staff. Delivered effectively by trained local facilitators. Role-play, brainstorming, small-group discussion to find shared solutions to common problems were helpful learning opportunities. No significant difference in DASS-21-V post-intervention.

Supplementary Online Table 5: Descriptive accounts

Authors	Country	Study design	Sample	Summary of findings
Sepúlveda et al., 2012 (178)	Chile	Descriptive account of community mental healthcare implementation, including PHC team training to treat people with SMI.	Admissions and consultations data showing reductions in emergency department psychiatric consultations.	A shift towards community mental healthcare was associated with reduced demand for emergency department psychiatric care. services. The proportion of emergency psychiatric consultations from the allocated catchment area fell consistently, compared with the adjacent catchment area, over 5 years The authors linked this to close working between secondary and PHC in this catchment area. Challenges: continuity of care, transferring skills and responsibilities from secondary care to district-based community mental health teams while developing the skills and expertise to support more complex patients, demonstrating cost-effectiveness through reliable data from different service levels.
Wenceslau & Ortega, 2015 (179)	Brazil	Descriptive account: integration of mental health into PHC.	N/A	Mental healthcare provided by community basic care teams, in 3 levels: 1) 'Matrix' technical support to basic healthcare services, 2) Training as a priority strategy and 3) including mental health in the basic care information system. The Matrix model promotes collaboration between specialists and families. 2011 psychosocial care network reorganised public health resources to further integrate mental health.
Toyama et al., 2017 (180)	Peru	Document review of mental health policy developments, summarising current initiatives and challenges. Documents describing the pathway to reform, plans and scope for scale-up were summarised.	Key documents pertaining to mental healthcare, issued by Peruvian government bodies.	Mental health gained policy and regulatory importance in 2004. Law 29889 (2012) facilitated mental health reform by restructuring mental health service delivery at primary and secondary care levels and introducing support services to aid recovery and societal reintegration. A performance-based budget was approved to guarantee implementation of these changes. Challenges: diversity of implementation settings (e.g. isolated rural areas), limited existing specialist mental health services, difficulty scaling up training and clinical support to every region.
Raviola et al., 2012 (181)	Haiti	Case study of a transnational team to expand mental health and psychosocial service delivery by 2 healthcare organisations: 1 local (*Zanmi Lasante*), 1 international (Partners in Health), collaborating during the 2010 Haiti earthquake emergency response.	N/A	In 18 months post-earthquake, *Zanmi Lasante* and Partners in Health provided 20,000 individual and group mental health and psychosocial appointments. Collaboratively developed a long-term model to expand and scale up community mental health services. The model applied evidence-based algorithms to treat CMDs and implementation rules: 1) assess context first, 2) identify priority care pathways, 3) specify decision-support tools, supervision, triage rules, 4) practice quality improvement and 5) plan for sustainability and capacity-building. The authors discuss wider collaborations and coordination with multiple organisations to plan implementation and delivery of mental healthcare. Strengths: linking research, training, advocacy to Zanmi Lasante and Partners in Health services, with academic centres.

Abel et al., 2012 (182)	English-speaking Caribbean (15 islands + Belize + Guyana)	Data extraction + desk review to assess mental health services + system development in English-speaking Caribbean. Data extracted from WHO-AIMS on mental health policies, services and systems.	Desk review + Medline (PubMed) search to review data on service utilisation.	English-speaking Caribbean countries and territories showed some improvement in mental health policy, service and system development. Proportion of health budgets spent on mental health as a ranged from 1-7%. PHC systems well-developed but mental health legislation required reform in many countries. Some countries developed innovative community secondary care treatment, integrated mental health into PHC and made psychotropic medication widely available in PHC. Greater focus on integrating mental health into PHC needed in some countries.
Semrau et al., 2011 (183)	European Region of WPA Task Force	Descriptive account of community mental healthcare development in Europe. Considers steps, obstacles, mistakes to avoid in implementing community mental healthcare.	53 countries across European region.	Mental health policy and legislation: present in most countries. Specific policies/strategies/plans for community mental health service development, downgrading large hospitals and integrating mental health into PHC, in two thirds of countries. Mental health research: suggests community-based mental healthcare is effective. However, limiting generalisability (most from UK). Overview of mental health services: few successful 'balanced-care models' integrating community and hospital services, especially in less affluent nations. Training for PHC staff only available in two thirds of countries.
Van Ommeren et al., 2015 (184)	Libya, Jordan	Descriptive account of specific actions for mental health and psychosocial support by the health sector in the preparedness, response and recovery phases of emergencies. Case studies on mental health and psychosocial support (Libya) and reorganisation of mental health services (Jordan) in humanitarian emergencies	N/A	Ministry of Health recommendations: 1) embed mental health + psychosocial support in national health and emergency preparedness plans, 2) create guidelines, standards, supporting tools for mental health and psychosocial support provision during emergencies (e.g. interventions in non-specialist health settings, CBT, IPT, stress management), psychological first aid, protection of people with SMI, 3) strengthen health professionals' capacity to identify and manage priority mental disorders during emergencies, 4) use emergency responses to develop sustainable mental healthcare.<br>Libya: A new mental health programme aimed to transform institution-based, centralised system. Goal: make mental healthcare available to everyone through staff capacity-building, including GP diploma in primary mental healthcare, psychologist diploma in psychotherapeutic interventions. Some recently trained professionals redeployed to train in new locations following political/security disruption.<br>Jordan: In addition to training general health workers at 21 PHC centres in 3 governorates using the mhGAP base course, with monthly supervision, psychiatrists, psychologists, social workers, and nurses provided comprehensive community care alongside four model acute inpatient units.<br>The Our Step Association (first service user/carer group) dedicated itself to supporting people through advocacy, awareness, fighting stigma, promoting mental health and human rights in the community. In 2013, they started using WHO QualityRights Toolkit to improve the rights of inpatients.

Chibanda, 2018 (185)	LMICs and HICs	Synthesis of reviews on MNS programmes integrated into PHC and community settings + digital/internet-based MNS interventions.	Studies addressing depression, alcohol use, neurocognitive disorders	Reviewed common features of promising programmes delivering mental healthcare via PHC, communities and digital platforms. Programmes from low-income countries: In addition to mhGAP and mhGAP-IG (PRIME study in Nigeria, Ethiopia, India, Nepal, South Africa, Uganda) implementation, Problem Management Plus (PM+), Friendship Bench (Zimbabwe, Malawi, Zanzibar, USA, Botswana), problem-solving therapy (PST), other forms of CBT, Healthy Activity Programme (HAP), MANAS (collaborative stepped-care intervention offering case management and psychosocial intervention), and group therapy were implemented. Programmes from high-income countries: Improving Access to Psychological Therapies (IAPT), a stepped care approach for depression and anxiety disorders, National Depression Detection and Treatment Programme, Access to Allied Psychological Services for young people.
Epping-Jordan et al., 2015 (186)	9 countries in the European region and South-East Asian region	Descriptive account of mental health system improvement following emergencies.	Descriptions by stakeholders from Kosovo, occupied Palestinian territory, Jordan, Iraq, Somalia, Burundi, Sri Lanka, Indonesia, Timor Leste, Afghanistan.	Cases demonstrated that emergency responses can make substantial, sustainable improvements to mental health systems. 10 common lessons: 1) mental health reform supported by long-term planning, 2) broad mental health needs of emergency-affected populations should be addressed, 3) respect the central role of government, 4) key role for national professionals, 5) imperative to coordinate across agencies, 6) mental health reform requires review and revision of national policies and plans, 7) consider and strengthen the mental health system as a whole, 8) reorganise and train health workers, 9) demonstration projects can show proof of concept, attracting support and funds for reform, 10) advocacy maintains momentum.
Esan et al. 2014 (187)	Gambia, Sierra Leone, Ghana, Nigeria, Liberia	Situational analysis of mental healthcare. Domains: mental health services, policies, mental disorder prevalence, rights legislation, financing, human resources, specialist services, medication, NGOs.	Sources: 1) stake-holders, 2) govt and epidemiological data, statistics, ‘grey’ data.	Mental health services, in various stages of development, were characterised by inadequate human resources and policy neglect. Despite few specialists, important regional contributions to community service development and global mental health research. Challenges: human resource development, policy and legislation updates, policies and budgeting. Although services grossly inadequate, given human and material constraints, signs of service development.
Weissbecker et al., 2018 (188)	Sierra Leone, Liberia	Case study of integrating mental health, psychosocial support into Ebola treatment units (ETUs). Intervention: 1) training, capacity building, 2) responding to distress, wellbeing, dignity, enhancing social connection integrated into ETUs, 3) psychosocial activities and interventions.	303 patients with Ebola virus disease.	In Sierra Leone, 55 patients reported mental disorder symptoms at least once during admission: low mood (78%), low appetite (69%), anxiety/worry (64%). 1:1 psychosocial support most frequently provided.<br>All patients with available data (n=30) had at least 1 psychosocial support interaction per day in Liberia; 66% (n=273) in Sierra Leone.

Goldsmith et al., 2019 (189)	South Sudan	Descriptive account of the mental health situational analysis between 2013-2016.	2 authors engaged in mental health and psychosocial support in-country.	Overview of mental health/psychosocial support services + research coordination mechanisms. Challenges: decreasing national healthcare budget in conflict zones, limited supervision, irregular pay and limited opportunities for healthcare personnel, concerns about training quality, including mhGAP-IG (e.g. over-prescribing, lack of post-training supervision), availability of psychotropic medication.<br>Recommendations: integrated, community-based model, collaboration between organisations engaged in reconciliation efforts and mental health service provision.
Jenkins et al., 2010 (190)	Kenya	Descriptive account of training PHC and CHWs on mental health. Intervention: mental health integrated into national PHC staff training.	1877 health workers trained.	Policy dialogue on PHC medicine supply, information systems, integrating mental health into services, national health and operational strategies also took place. Psychiatric and public health staff attended capacity-building workshops fostering integration of mental health into PHC under supervision.<br>Trainees from variety of staff cadres shared training feedback, including clinical utility, ability to increase knowledge and improve mental disorder detection, treatment, management.
Hanlon et al. 2010 (191)	African Region of WPA Task Force: 46 countries	Review of implementation of community mental healthcare across African countries, challenges and lessons learned by key regional experts.	Published service evaluations and regional experts' experience.	Despite recommendations, few examples of specialists mobilised to support mental healthcare in PHC. Requirements: Outreach, community sensitisation, engagement needed to detect mental health problems in community, mental healthcare close to homes. Opportunities: Traditional healers, religious leaders' involvement in community mental healthcare discussed but few evaluations. Contributions of service users and caregivers discussed but few evaluations. Challenges: Time pressure, biomedical emphasis, limited community resources lead to focus on medication. Competing priorities, decreasing community engagement, reliance on volunteers, under-funding, limited mental health professionals, concerns about staff skills, negative attitudes, sporadic medication. Lessons learned: need sustainable vision of care and optimise use of existing systems, government commitment, collaboration between stakeholders, local coordination, PHC staff supervision, integrated monitoring and evaluation.
Murray et al., 2011 (192)	12 countries: European, African, South-East Asian regions	Descriptive account based on authors' experience of implementing apprenticeship model of training and supervision of lay counsellors to deliver psychosocial interventions in LMICs.	Experience of training >100 lay counsellors.	Model implemented in Iraq, Sudan, Uganda, Burundi, Tanzania, Zambia, Sri Lanka, Pakistan, Nepal, Thailand, Cambodia, Indonesia.<br>Challenges: 1) supervisor attrition, 2) counsellor attrition, 3) limited capacity for handling clinical emergencies (e.g. suicidal or homicidal thoughts, child abuse), 4) time required, 5) need for shared language between supervisors and trainers.

Thornicroft & Semrau, 2019 (193)	Nigeria, Ethiopia, Uganda, South Africa, India, Nepal	Descriptive account of capacity building and evidence generating programme to strengthen health systems for improved mental healthcare.	Experience across six LMICs.	Components included (a) capacity building, (b) mental health financing, (c) integrated care, (d) mental health information systems, (e) knowledge transfer. Capacity building (a) entailed supporting PhD studies and Masters modules for researchers, policy-makers, planners, service users and carers in participating LMICs. Mental health financing (b) involved comprehensive evaluation of the costs associated with scaling up mental health in PHC. Integrated care (c) comprised scaling up mental healthcare in PHC, including examination of the barriers and facilitators. Mental health information systems (d) designed new mental health system indicators and proposed how they could be implemented. Knowledge transfer (e) shared learning about how to communicate the findings to decision-makers. Each component was summarised in one or more associated publications.
Van Ginneken et al., 2017 (194)	India	Case study comparing Indian models of mental healthcare by PHC staff and roles within these models. Aimed to identify feasible and acceptable models with potential for scale-up.	Interviews with PHC staff, coordinators, leaders, specialists and other staff (n=246), to explore programme structures, models of mental healthcare delivery and roles.	Programmes categorised using Bower framework of collaborative and non-collaborative models of primary mental healthcare. Collaborative care models: NGO-led multi-disciplinary teams, shared care between organisations. Non-collaborative programmes: (1) training models, (2) consultation-liaison models, (3) identification, referral and sensitisation models. Observations: Most collaborative and specialist community models used lay health workers rather than doctors. Both used care managers. PHC staff and care managers supported by multiple specialist and non-specialist voluntary sector and governmental organisations. Many training and identification/referral models did not provide supervision. Recommendations: new specialist community model, training PHC staff within specialist programmes to provide community support and treatment for people with SMI.

McGeorge, 2012 (195)	Australasia and Pacific Region of the WPA Task Force	Descriptive account of community mental healthcare in Australasia and the South Pacific, identifying steps, obstacles and mistakes to avoid when implementing community mental healthcare.	Australia, New Zealand, Micronesia, Melanesia, Polynesia.	Mental health services: 1) Similar delivery models in Australia and New Zealand. Pacific nations just beginning to establish mental health services, 2) mixture of local and regional, public and private mental health services in Australia and New Zealand but inadequately integrated. Mental health services in Pacific nations significantly underdeveloped. Mainly delivered through village or community-based PHC clinics; some linked to regional clinics/national hospitals.<br>Policies, plans, programmes: 1) government policy to build recovery-focused, community orientated services in Australia and New Zealand; no overall policy for all Pacific nations, 2) national mental health plans + variety of programmes addressing mental health promotion, anti-discrimination and stigma, prevention, psychiatric treatment, primary mental healthcare, recovery and service integration in Australia and New Zealand, 3) some Pacific Island nations have mental health policy/plans, few resourced and led to ensure implementation.<br>Achieving community-orientated services: 1) need more service-based research, 2) proportion of Australian state and territory community mental health spending increased from 29% to 51% by 2005. In New Zealand, 69% of mental health funding invested in community-based services by 2007, 3) continued difficulty accessing services and disruption to continuity of care 4) in Australia and New Zealand, community-based services supported by acute inpatient services in general and private hospitals and range of specialist community programmes (e.g. early intervention, maternal mental health, peer support, drug and alcohol services) via public and private mental health services and NGOs, 4) in Pacific nations, most mental healthcare delivered by generally trained medical and nursing practitioners. However, many Pacific countries do not provide mental health/addictions training, limited medication supply and considerable stigma about mental disorders.

Supplementary Online Table 6: Baseline surveys and exploratory studies

**Authors**	**Country**	**Study design**	**Sample**	**Summary of findings**
Adewuya et al., 2017 (196)	Nigeria	Survey of PHC staff knowledge/experience/competence in depression assessment/treatment, perceived causes, optimal treatment, staff role in management, attitudes towards people with depression.	Doctors (n=102), Nurses/midwives (n=285), HEWs/ community health officers (n=220)	>50% participants diagnosed depression from clinical vignette. Most thought mental illness causes were psychosocial or spiritual. 86% supported treating depression in PHC with enhanced capacity. Challenges: workload (69%), lack of training (68%). >42% had poor attitudes towards depression. Knowledge associated with training.
Abera et al., 2014 (197)	Ethiopia	Survey of challenges and opportunities for integrating mental health into PHC. Questions on past training, mental health experience, knowledge, attitudes to mental disorders, delivering mental healthcare in PHC.	PHC staff (n=151).	96% PHC staff thought mental healthcare was important. Most were interested in training, delivering mental healthcare. Greater training and exposure to mental healthcare associated with more favourable attitudes. Low knowledge of diagnoses, symptoms, treatments. 45% thought supernatural factors cause mental disorders. Requirements: medication, space, time, supervision, treatment guidelines.
Ahmed et al., 2019 (198)	Ethiopia	Survey of mental health knowledge, attitudes, practice and associated factors among urban health extension workers (HEWs).	Urban HEWs (n=455)	44% had 'adequate' knowledge, 93% had negative, 75% had 'good practice' towards mental ill-health. Older age, diploma education level, personal history of mental disorder negatively associated with knowledge. Job aid (e.g. leaflets, booklets, flipchart) and knowledge of mental disorders associated with increased service provision.
Wagenaar et al., 2015 (199)	Mozam-bique	Time series analyses of community ICD-10 diagnoses over time and by gender and health facility determinants of mental health service utilisation.	Census of community psychiatric consultations conducted at clinics in Sofala province (n=15,856).	Epilepsy: 49% of consultations, psychosis: 22%, stress-related disorders: 9%. Psychotic disorder reduced significantly over time (32% in 2012, 13% in 2014)/ epilepsy proportion increased. Stress-related diagnoses: 13% of women's consultations, 6% of men's. Substance use: 6% of men's, 2% of women's consultations. 2x more psychotic diagnoses in centres with more psychiatric technicians. Rural: fewer organic, substance use, psychosis, affective diagnoses, more epilepsy.

Acharya et al., 2016 (200)	Nepal	Qualitative study exploring PHC staff perspectives on embedding a mental health worker into community district general hospital + providing consultation from off-site psychiatrist. Focus group discussions explored concerns and recommendations for integrated mental healthcare + current psychiatric referral practice.	District general hospital PHC staff (n=24).	Existing system: referral to nearest psychiatrist (14 hours away). Participant concerns about integrated mental healthcare: hierarchies between mental health staff and other clinicians, impact of staff turnover on patients, reliability of off-site psychiatrist, ability of on-site PHC staff to screen patients + follow off-site psychiatrist advice. Participants suggested training existing PHC staff as dedicated mental healthcare workers to screen and refer, involving academics.
Mugisha et al., 2017 (201)	Ethiopia, Nepal, Nigeria, Uganda, South Africa, India	Qualitative document review exploring system level resources for integrating mental health into PHC: legislation, health policies/plans, relevant country health programmes.	Checklist based on WHO-AIMS-identified themes adapted to the country context applied to selected grey literature.	Ethiopia, Nepal, Nigeria, Uganda working towards mental health legislation. South Africa (2004) and India (2016) enacted Mental Health Acts. Only Nepal, Nigeria, South Africa had standalone mental health policies. Lack of policies likely to limit resource mobilisation, integrating mental health into PHC. Most budgets inadequate. Mental health human resources, monitoring and evaluation systems, health facilities inadequate in all countries.

Supplementary Online Table 7: Commentaries

Authors	Summary of findings
Abdulmalik et al., 2016 (202)	Description of a 'balanced model of care' considering available resources and context. Recommendations: task-sharing and greater utilisation of community mental health services. World Psychiatric Association guidance on developing community mental health services, training non-specialists in mental healthcare, and the Fundamental Sustainable Development Group (strengthening mental health in the development agenda) are proposed additional drivers of change.
Barnett et al., 2018 (203)	Description of a conceptual model of lay health worker training to address disparities in the supply of and demand for evidence-based treatments. Recommendations: 1) ensure that models meet local contextual needs 2) determine whether supply or demand determinants are the main drivers of service disparities, 3) ensure that future research addresses under-reporting of selection criteria, training and supervision details.
Dowrick, 2016 (204)	Summary of evidence for non-medical interventions and collaborative care for depression and related disorders as alternatives to antidepressant medication. Recommendations: effective management of severe and enduring illness (SMI) should focus on comorbid physical health problems. They emphasise the need for partnership working between specialist and PHC to monitor the physical health of people with SMI at least annually. The article summarises best practice for PHC assessment and management of dementia, including holistic review of care quality and collaborative care. The article supports integrating mental health into PHC in LMICs.
Farrington et al., 2014 (205)	The authors propose an integrated platform to support engagement with mobile phone technology in global mental healthcare. Recommendations: A platform should: 1) foster interdisciplinary collaboration across psychiatry, psychology, global health, IT, social science, stakeholders such as policymakers, industry and telecommunications, 2) integrate preventative, diagnostic, monitoring, therapeutic and educational functions, 3) employ a range of mobile capabilities, including SMS, voice calls, the latest smartphone sensors, provide a range of accessible and up-to-date resources for staff and patients, 4) consider cost-effectiveness; 5) facilitate cultural and local adaptation of interfaces and programmes. The authors argued that existing and developing technology are under-utilised in the global mental health sector.
Patel et al., 2018 (206)	Lancet Commission on global mental health and sustainable development: reframed agenda for the era of Sustainable Development Goals. 4 foundational pillars: 1) mental health is a global public good, relevant to global sustainable development irrespective of socioeconomic status, 2) a binary approach to mental disorders does not reflect the diversity and complexity of mental health needs, 3) the mental health of each individual is a product of social and environmental influences, interacting with genetic, neurodevelopmental and psychological processes, affecting brain pathways, 4) mental health is a human right for all people that requires a rights-based approach. These pillars require concrete actions: 1) mental health services should be scaled up as an essential component of universal health coverage and should be fully integrated into global responses to other health priorities, 2) barriers to mental health need to be addressed, 3) mental health should be protected by public policies and development efforts, 4) innovative non-specialist training and digital technologies should be considered and embraced, to deliver a range of interventions, 5) mental health should be invested in efficiently, 6) research and innovation should be invested in to harness novel approaches from diverse disciplines.
Salomone et al., 2019 (207)	WHO developed the Caregiver Skills Training (CST) programme for families of children with developmental disorders or delay. CST aimed to address growing evidence that caregivers can learn skills to support children's social communication and adaptive behaviour and reduce challenging behaviour. The authors describe the development process, content and field-testing strategy of WHO CST. The programme was evidence-based and designed for a global audience.
Thornicroft et al., 2010 (208)	A proposal for adult community mental healthcare. Recommendations: coordinated policies, plans and programmes, scaling up services for whole populations, community awareness about mental disorders to increase help-seeking, effective financial and budgetary provisions to directly support services. Lessons learned: solutions should target society, government, local mental health system, professional, practitioner, service user, family, and other advocate needs.

References

1. Araya R, Rojas G, Fritsch R, Gaete J, Rojas M, Simon G, et al. Treating depression in primary care in low-income women in Santiago, Chile: a randomised controlled trial. The Lancet. 2003;361(9362):995-1000.
2. Langer A, Farnot U, Garcia C, Barros F, Victora C, Belizan JM, et al. The Latin American trial of psychosocial support during pregnancy: Effects on mother's wellbeing and satisfaction. Social Science & Medicine. 1996;42(11):1589-97.
3. Robledo-Colonia AF, Sandoval-Restrepo N, Mosquera-Valderrama YF, Escobar-Hurtado C, Ramírez-Vélez R. Aerobic exercise training during pregnancy reduces depressive symptoms in nulliparous women: a randomised trial. Journal of physiotherapy. 2012;58(1):9-15.
4. Baker-Henningham H, Powell C, Walker S, Grantham-McGregor S. The effect of early stimulation on maternal depression: a cluster randomised controlled trial. Archives of disease in childhood. 2005;90(12):1230-4.
5. Babor TF, Grant M, Organization WH. Programme on substance abuse: Project on identification and management of alcohol-related problems. Report on Phase II, an randomized clinical trial of brief interventions in primary health care. World Health Organization; 1992.
6. Ernst CC, Grant TM, Streissguth AP, Sampson PD. Intervention with high-risk alcohol and drug-abusing mothers: II. Three-year findings from the Seattle model of paraprofessional advocacy. Journal of Community Psychology. 1999;27(1):19-38.
7. Rosenberg SA, Robinson C, Fryer GE. Evaluation of paraprofessional home visiting services for children with special needs and their families. Topics in Early Childhood Special Education. 2002;22(3):158-68.
8. Roman LA, Lindsay JK, Moore JS, Duthie PA, Peck C, Barton LR, et al. Addressing mental health and stress in Medicaid-insured pregnant women using a nurse-community health worker home visiting team. Public Health Nursing. 2007;24(3):239-48.
9. Roman LA, Gardiner JC, Lindsay JK, Moore JS, Luo Z, Baer LJ, et al. Alleviating perinatal depressive symptoms and stress: a nurse-community health worker randomized trial. Archives of women's mental health. 2009;12(6):379-91.
10. Walkup JT, Barlow A, Mullany BC, Pan W, Goklish N, Hasting R, et al. Randomized controlled trial of a paraprofessional-delivered in-home intervention for young reservation-based American Indian mothers. Journal of the American Academy of Child & Adolescent Psychiatry. 2009;48(6):591-601.
11. Waitzkin H, Getrich C, Heying S, Rodríguez L, Parmar A, Willging C, et al. Promotoras as mental health practitioners in primary care: a multi-method study of an intervention to address contextual sources of depression. Journal of community health. 2011;36(2):316-31.
12. Ginsburg GS, Barlow A, Goklish N, Hastings R, Baker EV, Mullany B, et al., editors. Postpartum depression prevention for reservation-based American Indians: results from a pilot randomized controlled trial. Child & youth care forum; 2012: Springer.
13. Barlow A, Mullany B, Neault N, Compton S, Carter A, Hastings R, et al. Effect of a paraprofessional home-visiting intervention on American Indian teen mothers’ and infants’ behavioral risks: a randomized controlled trial. American Journal of Psychiatry. 2013;170(1):83-93.
14. Barlow A, Mullany B, Neault N, Goklish N, Billy T, Hastings R, et al. Paraprofessional-delivered home-visiting intervention for American Indian teen mothers and children: 3-year outcomes from a randomized controlled trial. American Journal of Psychiatry. 2015;172(2):154-62.
15. Williamson AA, Knox L, Guerra NG, Williams KR. A pilot randomized trial of community-based parent training for immigrant Latina mothers. American Journal of Community Psychology. 2014;53(1-2):47-59.

16. Magaña S, Li H, Miranda E, Paradiso de Sayu R. Improving health behaviours of L atina mothers of youths and adults with intellectual and developmental disabilities. Journal of Intellectual Disability Research. 2015;59(5):397-410.
17. Moore AA, Karno MP, Ray L, Ramirez K, Barenstein V, Portillo MJ, et al. Development and preliminary testing of a promotora-delivered, Spanish language, counseling intervention for heavy drinking among male, Latino day laborers. Journal of substance abuse treatment. 2016;62:96-101.
18. Ekers D, Dawson M, Bailey E. Dissemination of behavioural activation for depression to mental health nurses: training evaluation and benchmarked clinical outcomes. Journal of psychiatric and mental health nursing. 2013;20(2):186-92.
19. Acarturk C, Uygun E, Ilkkursun Z, Carswell K, Tedeschi F, Batu M, et al. Effectiveness of a WHO self-help psychological intervention for preventing mental disorders among Syrian refugees in Turkey: a randomized controlled trial. World Psychiatry. 2022;21(1):88-95.
20. Purgato M, Carswell K, Tedeschi F, Acarturk C, Anttila M, Au T, et al. Effectiveness of self-help plus in preventing mental disorders in refugees and asylum seekers in Western Europe: a multinational randomized controlled trial. Psychotherapy and Psychosomatics. 2021;90(6):403-14.
21. Knefel M, Kantor V, Nicholson AA, Schiess-Jokanovic J, Weindl D, Schäfer I, et al. A brief transdiagnostic psychological intervention for Afghan asylum seekers and refugees in Austria: A randomized controlled trial. Trials. 2020;21(1).
22. Dybdahl R. Children and mothers in war: an outcome study of a psychosocial intervention program. Child development. 2001;72(4):1214-30.
23. Punamäki R-L, Peltonen K, Diab M, Qouta SR. Psychosocial interventions and emotion regulation among war-affected children: Randomized control trial effects. Traumatology. 2014;20(4):241.
24. Gavrilova SI, Ferri CP, Mikhaylova N, Sokolova O, Banerjee S, Prince M. Helping carers to care—The 10/66 dementia research group's randomized control trial of a caregiver intervention in Russia. International Journal of Geriatric Psychiatry: A journal of the psychiatry of late life and allied sciences. 2009;24(4):347-54.
25. Bolton P, Bass JK, Zangana GAS, Kamal T, Murray SM, Kaysen D, et al. A randomized controlled trial of mental health interventions for survivors of systematic violence in Kurdistan, Northern Iraq. BMC psychiatry. 2014;14(1):1-15.
26. Weiss WM, Murray LK, Zangana GAS, Mahmooth Z, Kaysen D, Dorsey S, et al. Community-based mental health treatments for survivors of torture and militant attacks in Southern Iraq: a randomized control trial. BMC psychiatry. 2015;15(1):1-16.
27. Bass J, Murray SM, Mohammed TA, Bunn M, Gorman W, Ahmed AMA, et al. A randomized controlled trial of a trauma-informed support, skills, and psychoeducation intervention for survivors of torture and related trauma in Kurdistan, Northern Iraq. Global Health Science and Practice. 2016;4(3):452-66.
28. Milani HS, Azargashb E, Beyraghi N, Defaie S, Asbaghi T. Effect of telephone-based support on postpartum depression: a randomized controlled trial. International journal of fertility & sterility. 2015;9(2):247.
29. Meffert SM, Abdo AO, Alla OAA, Elmakki YOM, Omer AA, Yousif S, et al. A pilot randomized controlled trial of interpersonal psychotherapy for Sudanese refugees in Cairo, Egypt. Psychological Trauma: Theory, Research, Practice, and Policy. 2014;6(3):240.
30. Puffer ES, Green E, Chase RM, Sim A, Zayzay J, Friis E, et al. Parents make the difference: a randomized-controlled trial of a parenting intervention in Liberia. Global Mental Health. 2015;2.
31. Betancourt TS, McBain R, Newnham EA, Akinsulure-Smith AM, Brennan RT, Weisz JR, et al. A behavioral intervention for war-affected youth in Sierra Leone: a randomized controlled trial. Journal of the American Academy of Child & Adolescent Psychiatry. 2014;53(12):1288-97.
32. Omeje JC, Otu MS, Aneke AO, Adikwu VO, Nwaubani OO, Chigbu EF, et al. Effect of rational emotive health therapy on alcohol use among community-dwelling, HIV-positive patients. Medicine. 2018;97(35).

33. Bass JK, Annan J, McIvor Murray S, Kaysen D, Griffiths S, Cetinoglu T, et al. Controlled trial of psychotherapy for Congolese survivors of sexual violence. New England Journal of Medicine. 2013;368(23):2182-91.
34. O'Callaghan P, McMullen J, Shannon C, Rafferty H, Black A. A randomized controlled trial of trauma-focused cognitive behavioral therapy for sexually exploited, war-affected Congolese girls. Journal of the American Academy of Child & Adolescent Psychiatry. 2013;52(4):359-69.
35. O'Callaghan P, Branham L, Shannon C, Betancourt TS, Dempster M, McMullen J. A pilot study of a family focused, psychosocial intervention with war-exposed youth at risk of attack and abduction in north-eastern Democratic Republic of Congo. Child Abuse & Neglect. 2014;38(7):1197-207.
36. Papas RK, Sidle JE, Gakinya BN, Baliddawa JB, Martino S, Mwaniki MM, et al. Treatment outcomes of a stage 1 cognitive–behavioral trial to reduce alcohol use among human immunodeficiency virus-infected out-patients in western Kenya. Addiction. 2011;106(12):2156-66.
37. Alexander CL, Arnkoff DB, Glass CR, Kaburu AW. Detecting depression in rural primary care clinics in central Kenya: Impact of a brief training intervention. International Perspectives in Psychology. 2013;2(1):14-28.
38. Jenkins R, Othieno C, Okeyo S, Kaseje D, Aruwa J, Oyugi H, et al. Short structured general mental health in service training programme in Kenya improves patient health and social outcomes but not detection of mental health problems-a pragmatic cluster randomised controlled trial. International journal of mental health systems. 2013;7(1):1-14.
39. L'Engle KL, Mwarogo P, Kingola N, Sinkele W, Weiner DH. A randomized controlled trial of a brief intervention to reduce alcohol use among female sex workers in Mombasa, Kenya. JAIDS Journal of Acquired Immune Deficiency Syndromes. 2014;67(4):446-53.
40. Bolton P, Bass J, Neugebauer R, Verdeli H, Clougherty KF, Wickramaratne P, et al. Group interpersonal psychotherapy for depression in rural Uganda: a randomized controlled trial. Jama. 2003;289(23):3117-24.
41. Bass J, Neugebauer R, Clougherty KF, Verdeli H, Wickramaratne P, Ndogoni L, et al. Group interpersonal psychotherapy for depression in rural Uganda: 6-month outcomes: randomised controlled trial. The British Journal of Psychiatry. 2006;188(6):567-73.
42. Bolton P, Bass J, Betancourt T, Speelman L, Onyango G, Clougherty KF, et al. Interventions for depression symptoms among adolescent survivors of war and displacement in northern Uganda: a randomized controlled trial. Jama. 2007;298(5):519-27.
43. Neuner F, Onyut PL, Ertl V, Odenwald M, Schauer E, Elbert T. Treatment of posttraumatic stress disorder by trained lay counselors in an African refugee settlement: a randomized controlled trial. Journal of consulting and clinical psychology. 2008;76(4):686.
44. Ertl V, Pfeiffer A, Schauer E, Elbert T, Neuner F. Community-implemented trauma therapy for former child soldiers in Northern Uganda: a randomized controlled trial. Jama. 2011;306(5):503-12.
45. Wagner GJ, Ghosh-Dastidar B, Robinson E, Ngo VK, Glick P, Mukasa B, et al. Effects of depression alleviation on ART adherence and HIV clinic attendance in Uganda, and the mediating roles of self-efficacy and motivation. AIDS and Behavior. 2017;21(6):1655-64.
46. Richards J, Foster C, Townsend N, Bauman A. Physical fitness and mental health impact of a sport-for-development intervention in a post-conflict setting: randomised controlled trial nested within an observational study of adolescents in Gulu, Uganda. BMC Public Health. 2014;14(1):1-13.
47. Singla DR, Kumbakumba E, Aboud FE. Effects of a parenting intervention to address maternal psychological wellbeing and child development and growth in rural Uganda: a community-based, cluster-randomised trial. The Lancet Global Health. 2015;3(8):e458-e69.
48. Kaaya SF, Blander J, Antelman G, Cyprian F, Emmons KM, Matsumoto K, et al. Randomized controlled trial evaluating the effect of an interactive group counseling intervention for HIV-positive women on prenatal depression and disclosure of HIV status. AIDS care. 2013;25(7):854-62.
49. Connolly S, Sakai C. Brief trauma intervention with Rwandan genocide-survivors using thought field therapy. International Journal of Emergency Mental Health. 2011;13(3):161.

50. Connolly SM, Roe-Sepowitz D, Sakai C, Edwards J. Utilizing community resources to treat PTSD: A randomized controlled study using Thought Field Therapy. African Journal of Traumatic Stress. 2013;3(1):24-32.
51. Yeomans PD, Forman EM, Herbert JD, Yuen E. A randomized trial of a reconciliation workshop with and without PTSD psychoeducation in Burundian sample. Journal of traumatic stress. 2010;23(3):305-12.
52. Kauye F, Jenkins R, Rahman A. Training primary health care workers in mental health and its impact on diagnoses of common mental disorders in primary care of a developing country, Malawi: a cluster-randomized controlled trial. Psychological medicine. 2014;44(3):657-66.
53. Murray LK, Skavenski S, Kane JC, Mayeya J, Dorsey S, Cohen JA, et al. Effectiveness of trauma-focused cognitive behavioral therapy among trauma-affected children in Lusaka, Zambia: a randomized clinical trial. JAMA pediatrics. 2015;169(8):761-9.
54. Chibanda D, Shetty AK, Tshimanga M, Woelk G, Stranix-Chibanda L, Rusakaniko S. Group problem-solving therapy for postnatal depression among HIV-positive and HIV-negative mothers in Zimbabwe. Journal of the International Association of Providers of AIDS Care (JIAPAC). 2014;13(4):335-41.
55. Chibanda D, Weiss HA, Verhey R, Simms V, Munjoma R, Rusakaniko S, et al. Effect of a primary care–based psychological intervention on symptoms of common mental disorders in Zimbabwe: a randomized clinical trial. JAMA. 2016;316(24):2618-26.
56. Igreja V, Kleijn WC, Schreuder BJ, Van Dijk JA, Verschuur M. Testimony method to ameliorate post-traumatic stress symptoms: Community-based intervention study with Mozambican civil war survivors. The British Journal of Psychiatry. 2004;184(3):251-7.
57. Wainberg ML. Community I-STAR Mozambique: community Implementation of SBIRT Using Technology for Alcohol Use Reduction in Mozambique. ClinicalTrialsgov. 2018.
58. Cooper PJ, Tomlinson M, Swartz L, Landman M, Molteno C, Stein A, et al. Improving quality of mother-infant relationship and infant attachment in socioeconomically deprived community in South Africa: randomised controlled trial. Bmj. 2009;338.
59. Rendall-Mkosi K, Morojele N, London L, Moodley S, Singh C, Girdler-Brown B. A randomized controlled trial of motivational interviewing to prevent risk for an alcohol-exposed pregnancy in the Western Cape, South Africa. Addiction. 2013;108(4):725-32.
60. Peltzer K, Naidoo P, Louw J, Matseke G, Zuma K, Mchunu G, et al. Screening and brief interventions for hazardous and harmful alcohol use among patients with active tuberculosis attending primary public care clinics in South Africa: results from a cluster randomized controlled trial. BMC public health. 2013;13(1):1-12.
61. le Roux IM, Tomlinson M, Harwood JM, O'CONNOR MJ, Worthman CM, Mbewu N, et al. Outcomes of home visits for pregnant mothers and their infants: a cluster randomised controlled trial. AIDS (London, England). 2013;27(9):1461.
62. Mertens JR, Ward CL, Bresick GF, Broder T, Weisner CM. Effectiveness of nurse-practitioner-delivered brief motivational intervention for young adult alcohol and drug use in primary care in South Africa: a randomized clinical trial. Alcohol and Alcoholism. 2014;49(4):430-8.
63. Mutyambizi-Mafunda V, Myers B, Sorsdahl K, Lund C, Naledi T, Cleary S. Integrating a brief mental health intervention into primary care services for patients with HIV and diabetes in South Africa: Study protocol for a trial-based economic evaluation. BMJ Open. 2019;9(5).
64. Ali BS, Rahbar MH, Naeem S, Gul A, Mubeen S, Iqbal A. The effectiveness of counseling on anxiety and depression by minimally trained counselors: a randomized controlled trial. American Journal of Psychotherapy. 2003;57(3):324-36.
65. Gul A, Ali B. The onset and duration of benefit from counselling by minimally trained counsellors on anxiety and depression in women. JPMA The Journal of the Pakistan Medical Association. 2004;54(11):549-52.

66. Rahman A, Iqbal Z, Roberts C, Husain N. Cluster randomized trial of a parent-based intervention to support early development of children in a low-income country. Child: care, health and development. 2009;35(1):56-62.
67. Rahman A, Malik A, Sikander S, Roberts C, Creed F. Cognitive behaviour therapy-based intervention by community health workers for mothers with depression and their infants in rural Pakistan: a cluster-randomised controlled trial. The Lancet. 2008;372(9642):902-9.
68. Hirani SS, Karmaliani R, McFarlane J, Asad N, Madhani F, Shehzad S, et al. Development of an economic skill building intervention to promote women's safety and child development in Karachi, Pakistan. Issues in mental health nursing. 2010;31(2):82-8.
69. Rahman A, Riaz N, Dawson KS, Usman Hamdani S, Chiumento A, Sijbrandij M, et al. Problem Management Plus (PM+): Pilot trial of a WHO transdiagnostic psychological intervention in conflict-affected Pakistan. World Psychiatry. 2016;15(2):182-3.
70. Rahman A, Hamdani SU, Awan NR, Bryant RA, Dawson KS, Khan MF, et al. Effect of a multicomponent behavioral intervention in adults impaired by psychological distress in a conflict-affected area of Pakistan: a randomized clinical trial. Jama. 2016;316(24):2609-17.
71. Rahman A, Khan MN, Hamdani SU, Chiumento A, Akhtar P, Nazir H, et al. Effectiveness of a brief group psychological intervention for women in a post-conflict setting in Pakistan: a single-blind, cluster, randomised controlled trial. The Lancet. 2019;393(10182):1733-44.
72. Hamdani SU, Masood A, Zhou K, Ahmed Z, Nazir H, Amin H, et al. Effect of adding a psychological intervention to routine care of common mental disorders in a specialized mental healthcare facility in Pakistan: a randomized controlled trial. International journal of mental health systems. 2021;15(1):1-12.
73. Rahman A, Divan G, Hamdani SU, Vajaratkar V, Taylor C, Leadbitter K, et al. Effectiveness of the parent-mediated intervention for children with autism spectrum disorder in south Asia in India and Pakistan (PASS): A randomised controlled trial. The Lancet Psychiatry. 2016;3(2):128-36.
74. Patel V, Chisholm D, Rabe-Hesketh S, Dias-Saxena F, Andrew G, Mann A. Efficacy and cost-effectiveness of drug and psychological treatments for common mental disorders in general health care in Goa, India: a randomised, controlled trial. The Lancet. 2003;361(9351):33-9.
75. Pal HR, Yadav D, Mehta S, Mohan I. A comparison of brief intervention versus simple advice for alcohol use disorders in a North India community-based sample followed for 3 months. Alcohol & Alcoholism. 2007;42(4):328-32.
76. Dias A, Dewey ME, D'Souza J, Dhume R, Motghare DD, Shaji K, et al. The effectiveness of a home care program for supporting caregivers of persons with dementia in developing countries: a randomised controlled trial from Goa, India. PloS one. 2008;3(6):e2333.
77. Tripathy P, Nair N, Barnett S, Mahapatra R, Borghi J, Rath S, et al. Effect of a participatory intervention with women's groups on birth outcomes and maternal depression in Jharkhand and Orissa, India: a cluster-randomised controlled trial. The Lancet. 2010;375(9721):1182-92.
78. Patel V, Weiss HA, Chowdhary N, Naik S, Pednekar S, Chatterjee S, et al. Effectiveness of an intervention led by lay health counsellors for depressive and anxiety disorders in primary care in Goa, India (MANAS): a cluster randomised controlled trial. The Lancet. 2010;376(9758):2086-95.
79. Chowdhary N, Anand A, Dimidjian S, Shinde S, Weobong B, Balaji M, et al. The healthy activity program lay counsellor delivered treatment for severe depression in India: Systematic development and randomised evaluation. British Journal of Psychiatry. 2016;208(4):381-8.
80. Fuhr DC, Weobong B, Lazarus A, Vanobberghen F, Weiss HA, Singla DR, et al. Delivering the Thinking Healthy Programme for perinatal depression through peers: an individually randomised controlled trial in India. The Lancet Psychiatry. 2019;6(2):115-27.
81. Muke SS, Tugnawat D, Joshi U, Anand A, Khan A, Shrivastava R, et al. Digital training for non-specialist health workers to deliver a brief psychological treatment for depression in primary care in India: Findings from a randomized pilot study. International Journal of Environmental Research and Public Health. 2020;17(17):1-22.

82. Wijesinghe CA, Williams SS, Kasturiratne A, Dolawaththa N, Wimalaratne P, Wijewickrema B, et al. A randomized controlled trial of a brief intervention for delayed psychological effects in snakebite victims. PLoS Negl Trop Dis. 2015;9(8):e0003989.
83. Puvimanasinghe TS, Price IR. Healing through giving testimony: An empirical study with Sri Lankan torture survivors. Transcultural psychiatry. 2016;53(5):531-50.
84. Sangraula M, Turner EL, Luitel NP, Van'T Hof E, Shrestha P, Ghimire R, et al. Feasibility of Group Problem Management plus (PM+) to improve mental health and functioning of adults in earthquake-affected communities in Nepal. Epidemiology and Psychiatric Sciences. 2020.
85. Li S. A report on a feasibility test of" community control of epilepsy" proposed by WHO. Zhonghua shen jing jing shen ke za zhi= Chinese journal of neurology and psychiatry. 1989;22(3):144-7, 90.
86. Gao L-l, Chan SW-c, Li X, Chen S, Hao Y. Evaluation of an interpersonal-psychotherapy-oriented childbirth education programme for Chinese first-time childbearing women: a randomised controlled trial. International journal of nursing studies. 2010;47(10):1208-16.
87. Gao L-l, Chan SW-c, Sun K. Effects of an interpersonal-psychotherapy-oriented childbirth education programme for Chinese first-time childbearing women at 3-month follow up: randomised controlled trial. International journal of nursing studies. 2012;49(3):274-81.
88. Tiwari A, Fong DYT, Yuen KH, Yuk H, Pang P, Humphreys J, et al. Effect of an advocacy intervention on mental health in Chinese women survivors of intimate partner violence: a randomized controlled trial. Jama. 2010;304(5):536-43.
89. Xu D, Xiao S, He H, Caine ED, Gloyd S, Simoni J, et al. Lay health supporters aided by mobile text messaging to improve adherence, symptoms, and functioning among people with schizophrenia in a resource-poor community in rural China (LEAN): A randomized controlled trial. PLoS Medicine. 2019;16(4).
90. Schwank SE, Chung H-F, Hsu M, Fu S-C, Du L, Zhu L, et al. Mental health of Urban Mothers (MUM) study: a multicentre randomised controlled trial, study protocol. 2020;10(11):e041133.
91. Chen C-H, Tseng Y-F, Chou F-H, Wang S-Y. Effects of support group intervention in postnatally distressed women: A controlled study in Taiwan. Journal of psychosomatic research. 2000;49(6):395-9.
92. Sherman SG, Sutcliffe C, Srirojn B, Latkin CA, Aramratanna A, Celentano DD. Evaluation of a peer network intervention trial among young methamphetamine users in Chiang Mai, Thailand. Social science & medicine. 2009;68(1):69-79.
93. Noknoy S, Rangsin R, Saengcharnchai P, Tantibhaedhyangkul U, McCambridge J. RCT of effectiveness of motivational enhancement therapy delivered by nurses for hazardous drinkers in primary care units in Thailand. Alcohol & Alcoholism. 2010;45(3):263-70.
94. Bolton P, Lee C, Haroz EE, Murray L, Dorsey S, Robinson C, et al. A transdiagnostic community-based mental health treatment for comorbid disorders: development and outcomes of a randomized controlled trial among Burmese refugees in Thailand. PLoS medicine. 2014;11(11):e1001757.
95. Assanangkornchai S, Nima P, McNeil EB, Edwards JG. Comparative trial of the WHO ASSIST-linked brief intervention and simple advice for substance abuse in primary care. Asian journal of psychiatry. 2015;18:75-80.
96. Shin J, Nhan N, Lee SB, Crittenden K, Flory M, Hong H. The effects of a home-based intervention for young children with intellectual disabilities in Vietnam. Journal of Intellectual Disability Research. 2009;53(4):339-52.
97. Bass J, Poudyal B, Tol W, Murray L, Nadison M, Bolton P. A controlled trial of problem-solving counseling for war-affected adults in Aceh, Indonesia. Social psychiatry and psychiatric epidemiology. 2012;47(2):279-91.
98. Armstrong G, Blashki G, Joubert L, Bland R, Moulding R, Gunn J, et al. An evaluation of the effect of an educational intervention for Australian social workers on competence in delivering brief

cognitive behavioural strategies: A randomised controlled trial. BMC Health Services Research. 2010;10(1):1-9.
99. Lyketsos CG, Taragano F, Treisman GJ, Paz J. Major depression and its response to sertraline in primary care vs. psychiatric office practice patients: Results of an open-label trial in Argentina. Psychosomatics. 1999;40(1):70-5.
100. Edelblute HB, Clark S, Mann L, McKenney KM, Bischof JJ, Kistler C. Promotoras across the border: A pilot study addressing depression in Mexican women impacted by migration. Journal of immigrant and minority health. 2014;16(3):492-500.
101. Han M, Valencia M, Lee YS, De Leon J. Development and implementation of the culturally competent program with Cambodians: the pilot psycho-social-cultural treatment group program. Journal of Ethnic And Cultural Diversity in Social Work. 2012;21(3):212-30.
102. Ayón C, Peña V, Naddy MBG. Promotoras' efforts to reduce alcohol use among Latino youths: Engaging Latino parents in prevention efforts. Journal of Ethnic and Cultural Diversity in Social Work. 2014;23(2):129-47.
103. Hovey JD, Hurtado G, Seligman LD. Findings for a CBT support group for Latina migrant farmworkers in Western Colorado. Current Psychology. 2014;33(3):271-81.
104. Zámbori J, Szádóczky E, Rózsa S, Furedi J. Cost-outcome of anxiety treatment intervention in primary care in Hungary. Journal of Mental Health Policy and Economics. 2002;5(3):115-20.
105. Loughry M, Ager A, Flouri E, Khamis V, Afana AH, Qouta S. The impact of structured activities among Palestinian children in a time of conflict. Journal of Child Psychology and Psychiatry. 2006;47(12):1211-8.
106. Fayyad JA, Farah L, Cassir Y, Salamoun MM, Karam EG. Dissemination of an evidence-based intervention to parents of children with behavioral problems in a developing country. European child & adolescent psychiatry. 2010;19(8):629-36.
107. Waterman S, Hunter ECM, Cole CL, Evans LJ, Greenberg N, Rubin GJ, et al. Training peers to treat Ebola centre workers with anxiety and depression in Sierra Leone. International Journal of Social Psychiatry. 2018;64(2):156-65.
108. Sow A, Criel B, Branger B, Roland M, De Spiegelaere M. Study on the integration of mental health support into primary health care in Guinea. Pan African Medical Journal. 2020;37:1-12.
109. Souza R, Yasuda S, Cristofani S. Mental health treatment outcomes in a humanitarian emergency: A pilot model for the integration of mental health into primary care in Habilla, Darfur. International Journal of Mental Health Systems. 2009;3.
110. Mutamba BB, Kane JC, De Jong JTVM, Okello J, Musisi S, Kohrt BA. Psychological treatments delivered by community health workers in low-resource government health systems: Effectiveness of group interpersonal psychotherapy for caregivers of children affected by nodding syndrome in Uganda. Psychological Medicine. 2018;48(15):2562-72.
111. O'Donnell K, Dorsey S, Gong W, Ostermann J, Whetten R, Cohen JA, et al. Treating maladaptive grief and posttraumatic stress symptoms in orphaned children in Tanzania: Group-based trauma-focused cognitive–behavioral therapy. Journal of traumatic stress. 2014;27(6):664-71.
112. Scholte WF, Verduin F, Kamperman AM, Rutayisire T, Zwinderman AH, Stronks K. The effect on mental health of a large scale psychosocial intervention for survivors of mass violence: a quasi-experimental study in Rwanda. PLoS One. 2011;6(8):e21819.
113. Murray LK, Familiar I, Skavenski S, Jere E, Cohen J, Imasiku M, et al. Evaluation of Trauma Focused Cognitive Behavioral Therapy for Children in Zambia. Child Abuse and Neglect. 2013;37(12):1175-85.
114. Chibanda D, Mesu P, Kajawu L, Cowan F, Araya R, Abas MA. Problem-solving therapy for depression and common mental disorders in Zimbabwe: piloting a task-shifting primary mental health care intervention in a population with a high prevalence of people living with HIV. BMC public health. 2011;11(1):1-10.

115. Futterman D, Shea J, Besser M, Stafford S, Desmond K, Comulada WS, et al. Mamekhaya: a pilot study combining a cognitive-behavioral intervention and mentor mothers with PMTCT services in South Africa. AIDS care. 2010;22(9):1093-100.
116. Van't Hof E, Stein DJ, Marks I, Tomlinson M, Cuijpers P. The effectiveness of problem solving therapy in deprived South African communities: Results from a pilot study. BMC Psychiatry. 2011;11.
117. Nimgaonkar AU, Menon SD. A task shifting mental health program for an impoverished rural Indian community. Asian journal of psychiatry. 2015;16:41-7.
118. Kaufman JA, Zeng W, Wang L, Zhang Y. Community-based mental health counseling for children orphaned by AIDS in China. AIDS care. 2013;25(4):430-7.
119. Paranthaman V, Satnam K, Lim J-L, Amar-Singh H, Sararaks S, Nafiza M-N, et al. Effective implementation of a structured psychoeducation programme among caregivers of patients with schizophrenia in the community. Asian journal of psychiatry. 2010;3(4):206-12.
120. Puspitosari WA, Wardaningsih S, Nanwani S. Improving the quality of life of people with schizophrenia through community based rehabilitation in Yogyakarta Province, Indonesia: A quasi experimental study. Asian Journal of Psychiatry. 2019;42:67-73.
121. Goncalves DA, Fortes S, Campos M, Ballester D, Portugal FB, Tófoli LF, et al. Evaluation of a mental health training intervention for multidisciplinary teams in primary care in Brazil: A pre- and posttest study. General Hospital Psychiatry. 2013;35(3):304-8.
122. Anderson P, O'Donnell A, Kaner E, Gual A, Schulte B, Pérez Gómez A, et al. Scaling-up primary health care-based prevention and management of heavy drinking at the municipal level in middle-income countries in Latin America: Background and protocol for a three-country quasi-experimental study. F1000Research. 2017;6.
123. Cook PF, Manzouri S, Aagaard L, O’Connell L, Corwin M, Gance-Cleveland B. Results from 10 years of interprofessional training on motivational interviewing. Evaluation & the health professions. 2017;40(2):159-79.
124. MacCarthy D, Weinerman R, Kallstrom L, Kadlec H, Hollander MJ, Patten S. Mental health practice and attitudes of family physicians can be changed! The Permanente Journal. 2013;17(3):14.
125. Ravitz P, Cooke RG, Mitchell S, Reeves S, Teshima J, Lokuge B, et al. Continuing education to go: capacity building in psychotherapies for front-line mental health workers in underserviced communities. The Canadian Journal of Psychiatry. 2013;58(6):335-43.
126. Ruud T, Flage KB, Kolbjørnsrud O-B, Haugen GB, Sørlie T. A Two-Year Multidisciplinary Training Program for the Frontline Workforce in Community Treatment of Severe Mental Illness. Psychiatric Services. 2016;67(1):7-9.
127. Bowers A, Burnett F. Developing, delivering and evaluating training sessions for primary care mental health link workers. Mental Health Nursing (Online). 2009;29(1):10.
128. Ferraz H, Wellman N. Fostering a culture of engagement: an evaluation of a 2-day training in solution-focused brief therapy for mental health workers. Journal of psychiatric and mental health nursing. 2009;16(4):326-34.
129. Chew-Graham C, Burroughs H, Hibbert D, Gask L, Beatty S, Gravenhorst K, et al. Aiming to improve the quality of primary mental health care: developing an intervention for underserved communities. BMC Family Practice. 2014;15(1):1-9.
130. Sadik S, Abdulrahman S, Bradley M, Jenkins R. Integrating mental health into primary health care in Iraq. Mental Health in Family Medicine. 2011;8(1):39.
131. De Jong JT. A comprehensive public mental health programme in Guinea-Bissau: a useful model for African, Asian and Latin-American countries. Psychological Medicine. 1996;26(1):97-108.
132. Adeyemi J, Jegede R. Integrating psychiatry into primary care: an experimental model. Social psychiatry and psychiatric epidemiology. 1994;29(6):277-81.
133. Odejide AO, Morakinyo JJ, Oshiname FO, Omigbodun O, Ajuwon AJ, Kola L. Integrating mental health into primary health care in Nigeria: management of depression in a local government (district) area as a paradigm. Seishin shinkeigaku zasshi= Psychiatria et neurologia Japonica. 2002;104(10):802-9.

134. Omigbodun O, Bella T, Dogra N, Simoyan O. Training health professionals for child and adolescent mental health care in Nigeria: a qualitative analysis. Child and adolescent mental health. 2007;12(3):132-7.
135. Abayomi O, Adelufosi A, Olajide A. Changing attitude to mental illness among community mental health volunteers in south-western Nigeria. International Journal of Social Psychiatry. 2013;59(6):609-12.
136. Makanjuola V, Doku V, Jenkins R, Gureje O. Impact of a one-week intensive 'training of trainers' workshop for community health workers in south-west Nigeria. Mental health in family medicine. 2012;9(1):33.
137. Tilahun D, Fekadu A, Tekola B, Araya M, Roth I, Davey B, et al. Ethiopian community health workers' beliefs and attitudes towards children with autism: Impact of a brief training intervention. Autism. 2019;23(1):39-49.
138. Kabura P, Fleming LM, Tobin DJ. Microcounseling skills training for informal helpers in Uganda. International Journal of Social Psychiatry. 2005;51(1):63-70.
139. Abbo C, Okello E, Nakku J. Effect of brief training on reliability and applicability of Global Assessment of functioning scale by Psychiatric clinical officers in Uganda. African health sciences. 2013;13(1):78-81.
140. Jenkins R, Kiima D, Okonji M, Njenga F, Kingora J, Lock S. Integration of mental health into primary care and community health working in Kenya: context, rationale, coverage and sustainability. Mental Health in Family Medicine. 2010;7(1):37.
141. Cettomai D, Kwasa J, Birbeck GL, Price RW, Bukusi EA, Meyer A-C. Training needs and evaluation of a neuro-HIV training module for non-physician healthcare workers in western Kenya. Journal of the neurological sciences. 2011;307(1-2):92-6.
142. Kauye F, Chiwandira C, Wright J, Common S, Phiri M, Mafuta C, et al. Increasing the capacity of health surveillance assistants in community mental health care in a developing country, Malawi. Malawi Medical Journal. 2011;23(3):85-8.
143. Wright J, Common S, Kauye F, Chiwandira C. Integrating community mental health within primary care in southern Malawi: A pilot educational intervention to enhance the role of health surveillance assistants. International Journal of Social Psychiatry. 2014;60(2):155-61.
144. Byrne H, Olkers P, Flisher AJ. Training in child and adolescent psychiatry for primary health care nurses in South Africa. Journal of child and adolescent mental health. 2004;16(2):83-6.
145. Bradshaw T, Mairs H, Richards D. Developing mental health education for health volunteers in a township in South Africa. Primary Health Care Research & Development. 2006;7(2):95-105.
146. Armstrong G, Kermode M, Raja S, Suja S, Chandra P, Jorm AF. A mental health training program for community health workers in India: Impact on knowledge and attitudes. International Journal of Mental Health Systems. 2011;5.
147. Paudel S, Gilles N, Hahn S, Hexom B, Premkumar R, Arole S, et al. Impact of mental health training on village health workers regarding clinical depression in rural India. Community mental health journal. 2014;50(4):480-6.
148. Hofmann-Broussard C, Armstrong G, Boschen MJ, Somasundaram KV. A mental health training program for community health workers in India: impact on recognition of mental disorders, stigmatizing attitudes and confidence. International Journal of Culture and Mental Health. 2017;10(1):62-74.
149. Malla A, Margoob M, Iyer S, Majid A, Lal S, Joober R, et al. Testing the Effectiveness of Implementing a Model of Mental Healthcare Involving Trained Lay Health Workers in Treating Major Mental Disorders Among Youth in a Conflict-Ridden, Low-Middle Income Environment: Part II Results. Canadian Journal of Psychiatry. 2019;64(9):630-7.
150. Jordans MJ, Luitel NP, Poudyal B, Tol WA, Komproe IH. Evaluation of a brief training on mental health and psychosocial support in emergencies: a pre-and post-assessment in Nepal. Prehospital and disaster medicine. 2012;27(3):235-8.

151. Li J, Li J, Huang Y, Thornicroft G. Mental health training program for community mental health staff in Guangzhou, China: effects on knowledge of mental illness and stigma. International Journal of Mental Health Systems. 2014;8(1):1-6.
152. Lam TP, Mak KY, Lam KF, Chan HY, Sun KS. Five-year outcomes of western mental health training for Traditional Chinese Medicine practitioners. BMC psychiatry. 2016;16(1):1-8.
153. Yang BX, Stone TE, Davis SA. The effect of a community mental health training program for multidisciplinary staff. Archives of Psychiatric Nursing. 2018;32(3):413-7.
154. Graham AL, Julian J, Meadows G. Improving responses to depression and related disorders: Evaluation of a innovative, general, mental health care workers training program. International Journal of Mental Health Systems. 2010;4.
155. Hossain D, Gorman D, Eley R, Coutts J. Value of mental health first aid training of advisory and extension agents in supporting farmers in rural Queensland. Rural and Remote Health. 2010;10(4):1593-603.
156. Morawska A, Fletcher R, Pope S, Heathwood E, Anderson E, McAuliffe C. Evaluation of mental health first aid training in a diverse community setting. International Journal of Mental Health Nursing. 2013;22(1):85-92.
157. Agrest M, Le PD, Yang LH, Mascayano F, Alves-Nishioka S, Dev S, et al. Implementing a community-based task-shifting psychosocial intervention for individuals with psychosis in Chile: Perspectives from users. International Journal of Social Psychiatry. 2019;65(1):38-45.
158. Zeligman M, Shannonhouse LR, Scherma H, Luke M, Schweiger W, Wanna RS. Mental Health Facilitator (MHF) Implementation in Mexico: Perceptions of Facilitators and Beneficiaries. International Journal for the Advancement of Counselling. 2019;41(3):389-406.
159. Church EA, Heath OJ, Curran VR, Bethune C, Callanan TS, Cornish PA. Rural professionals' perceptions of interprofessional continuing education in mental health. Health & social care in the community. 2010;18(4):433-43.
160. Fendt-Newlin ML. Enhancing mental health practice in Sierra Leone: A social intervention development study: University of York; 2018.
161. Waterman S, Cole CL, Greenberg N, Rubin GJ, Beck A. A qualitative study assessing the feasibility of implementing a group cognitive–behavioural therapy-based intervention in Sierra Leone. BJPsych international. 2019;16(2):31-4.
162. Adams B, Vallières F, Duncan JA, Higgins A, Eaton J. Stakeholder perspectives of Community Mental Health Forums: A qualitative study in Sierra Leone. International Journal of Mental Health Systems. 2020;14(1).
163. Makanjuola V, Doku V, Jenkins R, Gureje O. Monitoring and evaluation of the activities of trainees in the 'training of trainers' workshop at Ibadan, south-west Nigeria. Mental health in family medicine. 2012;9(1):25.
164. Mutamba BB, Kohrt BA, Okello J, Nakigudde J, Opar B, Musisi S, et al. Contextualization of psychological treatments for government health systems in low-resource settings: Group interpersonal psychotherapy for caregivers of children with nodding syndrome in Uganda. Implementation Science. 2018;13(1).
165. Papas RK, Sidle JE, Martino S, Baliddawa JB, Songole R, Omolo OE, et al. Systematic cultural adaptation of cognitive-behavioral therapy to reduce alcohol use among HIV-infected outpatients in western Kenya. AIDS and Behavior. 2010;14(3):669-78.
166. Jenkins R, Othieno C, Okeyo S, Aruwa J, Wallcraft J, Jenkins B. Exploring the perspectives and experiences of health workers at primary health facilities in Kenya following training. International Journal of Mental Health Systems. 2013;7(1):1-8.
167. Othieno C, Jenkins R, Okeyo S, Aruwa J, Wallcraft J, Jenkins B. Perspectives and concerns of clients at primary health care facilities involved in evaluation of a national mental health training programme for primary care in Kenya. International Journal of Mental Health Systems. 2013;7(1):1-7.

168. Petersen I, Ssebunnya J, Bhana A, Baillie K. Lessons from case studies of integrating mental health into primary health care in South Africa and Uganda. International Journal of Mental Health Systems. 2011;5.
169. Petersen I, Bhana A, Baillie K. The feasibility of adapted group-based interpersonal therapy (IPT) for the treatment of depression by community health workers within the context of task shifting in South Africa. Community mental health journal. 2012;48(3):336-41.
170. Sorsdahl K, Myers B, Ward CL, Matzopoulos R, Mtukushe B, Nicol A, et al. Adapting a blended motivational interviewing and problem-solving intervention to address risky substance use amongst South Africans. Psychotherapy research. 2015;25(4):435-44.
171. Myers B, Petersen-Williams P, van der Westhuizen C, Lund C, Lombard C, Joska JA, et al. Community health worker-delivered counselling for common mental disorders among chronic disease patients in South Africa: a feasibility study. BMJ open. 2019;9(1):e024277.
172. Spedding M, Stein DJ, Naledi T, Myers B, Cuijpers P, Sorsdahl KR. A task-sharing intervention for prepartum common mental disorders: Feasibility, acceptability and responses in a South African sample. African Journal of Primary Health Care and Family Medicine. 2020;12(1):2071-928.
173. Abas M, Bowers T, Manda E, Cooper S, Machando D, Verhey R, et al. 'Opening up the mind': problem-solving therapy delivered by female lay health workers to improve access to evidence-based care for depression and other common mental disorders through the Friendship Bench Project in Zimbabwe. International journal of mental health systems. 2016;10(1):1-8.
174. Nyamayaro P, Bere T, Magidson JF, Simms V, O'Cleirigh C, Chibanda D, et al. A Task-Shifting Problem-Solving Therapy Intervention for Depression and Barriers to Antiretroviral Therapy Adherence for People Living With HIV in Zimbabwe: Case Series. Cognitive and Behavioral Practice. 2020;27(1):84-92.
175. Pereira B, Andrew G, Pednekar S, Kirkwood BR, Patel V. The integration of the treatment for common mental disorders in primary care: Experiences of health care providers in the MANAS trial in Goa, India. International Journal of Mental Health Systems. 2011;5.
176. Muke SS, Shrivastava RD, Mitchell L, Khan A, Murhar V, Tugnawat D, et al. Acceptability and feasibility of digital technology for training community health workers to deliver brief psychological treatment for depression in rural India. Asian Journal of Psychiatry. 2019;45:99-106.
177. Fisher J, Nguyen H, Mannava P, Tran H, Dam T, Tran H, et al. Translation, cultural adaptation and field-testing of the thinking healthy program for Vietnam. Globalization and Health. 2014;10(1).
178. Sepúlveda R, Ramírez J, Zitko P, Ortiz A, Norambuena P, Barrera Á, et al. Implementing the community mental health care model in a large latin-american urban area: The experience from Santiago, Chile. International Journal of Mental Health. 2012;41(1):62-72.
179. Wenceslau LD, Ortega F. Mental health within primary health care and Global Mental Health: International perspectives and Brazilian context. Interface: Communication, Health, Education. 2015;19(55):1121-32.
180. Toyama M, Castillo H, Galea JT, Brandt LR, Mendoza M, Herrera V, et al. Peruvian mental health reform: A framework for scaling-up mental health services. International Journal of Health Policy and Management. 2017;6(9):501-8.
181. Raviola G, Eustache E, Oswald C, Belkin GS. Mental health response in Haiti in the aftermath of the 2010 earthquake: A case study for building long-term solutions. Harvard Review of Psychiatry. 2012;20(1):68-77.
182. Abel WD, Kestel D, Eldemire-Shearer D, Sewell C, Whitehorne-Smith P. Mental health policy and service system development in the english-speaking caribbean. West Indian Medical Journal. 2012;61(5):475-82.
183. Semrau M, Barley EA, Law A, Thornicroft G. Lessons learned in developing community mental health care in Europe. World Psychiatry. 2011;10(3):217-25.
184. van Ommeren M, Hanna F, Weissbecker I, Ventevogel P. Mental health and psychosocial support in humanitarian emergencies. Eastern Mediterranean Health Journal. 2015;21(7):498-502.

185. Chibanda D. Programmes that bring mental health services to primary care populations in the international setting. International Review of Psychiatry. 2018;30(6):170-81.
186. Epping-Jordan JAE, van Ommeren M, Ashour HN, Maramis A, Marini A, Mohanraj A, et al. Beyond the crisis: Building back better mental health care in 10 emergency-affected areas using a longer-term perspective. International Journal of Mental Health Systems. 2015;9(1).
187. Esan O, Abdumalik J, Eaton J, Kola L, Fadahunsi W, Gureje O. Mental health care in Anglophone West Africa. Psychiatric Services. 2014;65(9):1084-7.
188. Weissbecker I, Roshania R, Cavallera V, Mallow M, Leichner A, Antigua J, et al. Integrating psychosocial support at Ebola treatment units in Sierra Leone and Liberia. Intervention. 2018;16(2):69-78.
189. Goldsmith A, Cockcroft-McKay C. Mental health in South Sudan: a case for community-based support. Disasters. 2019;43(3):534-54.
190. Jenkins R, Kiima D, Njenga F, Okonji M, Kingora J, Kathuku D, et al. Integration of mental health into primary care in Kenya. World Psychiatry. 2010;9(2):118.
191. Hanlon C, Wondimagegn D, Alem A. Lessons learned in developing community mental health care in Africa. World Psychiatry. 2010;9(3):185-9.
192. Murray LK, Dorsey S, Bolton P, Jordans MJD, Rahman A, Bass J, et al. Building capacity in mental health interventions in low resource countries: An apprenticeship model for training local providers. International Journal of Mental Health Systems. 2011;5.
193. Thornicroft G, Semrau M. Health system strengthening for mental health in low-and middle-income countries: introduction to the Emerald programme. BJPsych Open. 2019;5(5).
194. Van Ginneken N, Maheedhariah MS, Ghani S, Ramakrishna J, Raja A, Patel V. Human resources and models of mental healthcare integration into primary and community care in India: Case studies of 72 programmes. PloS one. 2017;12(6).
195. McGeorge P. Lessons learned in developing community mental health care in Australasia and the South Pacific. World Psychiatry. 2012;11(2):129-32.
196. Adewuya AO, Adewumi T, Ola B, Abosede O, Oyeneyin A, Fasawe A, et al. Primary health care workers' knowledge and attitudes towards depression and its management in the MeHPric-P project, Lagos, Nigeria. General Hospital Psychiatry. 2017;47:1-6.
197. Abera M, Tesfaye M, Belachew T, Hanlon C. Perceived challenges and opportunities arising from integration of mental health into primary care: a cross-sectional survey of primary health care workers in south-west Ethiopia. BMC health services research. 2014;14:113.
198. Ahmed E, Merga H, Alemseged F. Knowledge, attitude, and practice towards mental illness service provision and associated factors among health extension professionals in Addis Ababa, Ethiopia. International Journal of Mental Health Systems. 2019;13(1).
199. Wagenaar BH, Cumbe V, Raunig-Berhó M, Rao D, Napúa M, Hughes JP, et al. Health facility determinants and trends of ICD-10 outpatient psychiatric consultations across Sofala, Mozambique: Time-series analyses from 2012 to 2014. BMC Psychiatry. 2015;15(1).
200. Acharya B, Tenpa J, Thapa P, Gauchan B, Citrin D, Ekstrand M. Recommendations from primary care providers for integrating mental health in a primary care system in rural Nepal. BMC health services research. 2016;16(1):1-8.
201. Mugisha J, Abdulmalik J, Hanlon C, Petersen I, Lund C, Upadhaya N, et al. Health systems context (s) for integrating mental health into primary health care in six Emerald countries: a situation analysis. International Journal of Mental Health Systems. 2017;11(1):1-13.
202. Abdulmalik J, Thornicroft G. Community mental health: a brief, global perspective. Neurology Psychiatry and Brain Research. 2016;22(2):101-4.
203. Barnett ML, Lau AS, Miranda J. Lay Health Worker Involvement in Evidence-Based Treatment Delivery: A Conceptual Model to Address Disparities in Care. Annual Review of Clinical Psychology2018. p. 185-208.
204. Dowrick C. Update on advances in psychiatric treatment in primary care. BJ Psych Advances. 2016;22(2):99-107.

205. Farrington C, Aristidou A, Ruggeri K. MHealth and global mental health: Still waiting for the mH2 wedding? Globalization and Health. 2014;10(1).
206. Patel V, Saxena S, Lund C, Thornicroft G, Baingana F, Bolton P, et al. The Lancet Commission on global mental health and sustainable development. The Lancet. 2018;392(10157):1553-98.
207. Salomone E, Pacione L, Shire S, Brown FL, Reichow B, Servili C. Development of the WHO Caregiver Skills Training Program for Developmental Disorders or Delays. Frontiers in Psychiatry. 2019;10.
208. Thornicroft G, Alem A, Dos Santos RA, Barley E, Drake RE, Gregorio G, et al. WPA guidance on steps, obstacles and mistakes to avoid in the implementation of community mental health care. World Psychiatry. 2010;9(2):67-77.
